# Uricase deficiency in rats results in a variety of metabolic disorders, addition to gouty nephropathy

**DOI:** 10.1371/journal.pone.0330344

**Published:** 2025-08-22

**Authors:** Xulian Wan, Yu Yun, Shixiong Li, Guanyun Luo, Ning Li, Hua Yin, Weigang Duan

**Affiliations:** 1 School of Basic Medicine, Yunnan University of Chinese Medicine, Kunming, Yunnan, China; 2 School of Basic Medicine, Kunming Medical University, Kunming, Yunnan, China; Federal Fluminense University: Universidade Federal Fluminense, BRAZIL

## Abstract

Clinical evidence suggests that hyperuricemia is frequently associated with hyperglycemia (diabetes), hyperlipidemia, and hypertension. However, this relationship has not been fully verified in experimental animals. The present study used uricase-deficient rats (KDY rats, n = 125) with spontaneously elevated levels of serum uric acid (SUA) as the model animals and investigated their metabolic conditions throughout their lifespan (626 days of age). The serum, urine and feces of the rats were collected, histological examination was performed using hematoxylin-eosin or Masson’s staining, and gene expression was determined using transcriptome high-throughput sequencing. Compared with wild type (WT) rats of the same age, the SUA levels in KDY rats were continuously high (approximately 70 μg/mL), and the body weight gain slowed after 45 days of age, followed by increased urine output, diabetes mellitus (hyperglycemia), high low-density lipoprotein, and hypercholesterolemia. Histological examination showed that gouty nephropathy appeared after approximately 45 days of age, before the rats developed medullary injury, medullary interstitial fibrosis, cortical glomerulus injury, and glomerular fibrosis. KDY rats also showed signs of atherosclerosis and hypertension in the late stage of their lifespan. The lifespan of KDY rats was significantly shorter than that of WT rats (more than 626 days). The expected lifespan of KDY rats is approximately 450 days, and the direct cause of the shortened lifespan is renal failure caused by gout nephropathy. The direct mechanisms of the lesions in KDY are related to the upregulation of various of inflammatory (immune) pathways. In conclusion, it demonstrated that hyperuricemia in KDY rats leads to type 2 diabetes mellitus (hyperglycemia), hyperlipidemia, atherosclerosis, and hypertension, in addition to gouty nephropathy.

## Introduction

Following hypertension, hyperglycemia, and hyperlipidemia, hyperuricemia has become the fourth factor seriously threatening people’s health [1]. Moreover, clinical studies have shown that hyperuricemia is closely related to the other three disorders [2–4]. However, because mice [5] and rat [6], commonly used as experimental animals, express uricase which is a crucial factor in preventing the increase of uric acid by degrading uric acid [7], the natural relationship between hyperuricemia and the other three disorders (especially diabetes mellitus [5,6]) not supported by robust experimental evidence, and few experimental studies support the relationship.

Humans are naturally uricase-deficient, which is an important prerequisite for hyperuricemia susceptibility. Hyperuricemia can be diagnosed if the level of serum uric acid (SUA) persists above 70 μg/mL in both sexes [8]. However, because uricase is expressed in wild-type (WT) mice and rats, it is difficult to create a real hyperuricemic animal model (SUA above 70 μg/mL) with similar organ injuries. This is also the case even if they are fed a high-purine diet to supply substrates of uric acid like adenine [9], administered drugs (e.g. potassium oxonate [9,10]) to inhibit uricase, or administered drugs (e.g. ethambutol [11]) to inhibit the excretion of uric acid via urine.

Fortunately, as early as 2018, uricase-deficient animals were obtained based on inbred animals (C57BL/6J mice) using transcription activator-like effector nucleases (TALEN) technology [12], an advanced technique that is better than the homologous recombination technique [13]. Animals can stably generate offspring but with extensive organ damage, especially in the kidneys [12]. However, the animals only exhibit a trend of abnormal levels of blood glucose and lipid when they reach maturity, and the values are still in the reference ranges [12]. In 2019, uricase-deficient rats (named Kunming-DY, [KDY] rats) were obtained based on Sprague-Dawley (SD) rats using a CRISPR/Cas9 technique [14]. The animals were reproductive, and approximately 90% of them survive one-year or more after they are weaned [14]. KDY rats were generated based on closed colony animals rather than inbred animals; thus, they retained a certain degree of genetic diversity. Unlike in uricase-deficient mice [12], the degree of elevated SUA in male KDY rats was stable and more similar to that observed in adult men [15]. That is, approximately half of the male animals had SUA above the level of 70 μg/mL. Thus, KDY rats are convenient for studying the relationship between hyperuricemia and other metabolic disorders.

## Materials and methods

### Materials

KDY rats were generated by our team [14] and allowed to breed. One male KDY rat was mated with one or two female KDY rats to generate offspring of KDY rats. After the female rats became pregnant, they were raised in individually in cages. When the offspring rats were 21 days of age, male and female were weaned and raised separately. When the offspring rats were 45 days old, samples of blood, urine, and feces were collected at the scheduled time points to detect relevant indices. WT rats (SD rats) (Certification No. SCXK [Chuan] 2008–24), used as the control, were provided by Chengdu Dossy Experimental Animals Co. Ltd. (Chengdu, China) and bred according to the specific pathogen free (SPF) standard. The animals were kept in an environment with a 12-h/12-h cycle (12 h mimicking natural light and 12 h of dark), a temperature of 21–23°C, a humidity of 45–75%, and good ventilation. All animals were provided free access to water and food. The sterile food was produced by Suzhou Shuangshi Experimental Animal Food Co., Ltd., according to the Chinese standard for experimental rat food (GB-14924.3–2010).

Uric acid assay kits using the phosphotungstic acid method (Lot: C012-1–1), glucose assay kits using the glucose oxidase method (A154-1–1), low-density lipoprotein (LDL) assay kits (A113-1–1), high-density lipoprotein (HDL) assay kits (A112-1–1), triglyceride (TG) assay kits (A110-1–1), total cholesterol (TC) assay kits (A111-1–1), total amino acid detection kit (A026-1–1), creatinine (Cr) assay kits using the sarcosine oxidase method (C011-2–1), urea assay kits using the urease method (C013-2–1), D-lactate assay kit (A019-3–1), lipopolysaccharide (LPS) ELISA kit, total amino acid (TAA) assay kit (A026-1–1), and hematoxylin-eosin (HE) staining kit were produced by Nanjing Jiancheng Bioengineering Institute (Nanjing, China). Glucagon ELISA kit (Meimian-9058) was produced by Jiangsu Meimian Immunoassay Industry Co. Ltd. (Yancheng, China). Insulin ELISA kit (RX302147R), C-reactive protein (CRP) ELISA kit (RX302991R), and high mobility group box 1 (HMGB1) ELISA kit (RX302701R) were produced by Ruixin Biotech Co. Ltd. (Quanzhou, China). The TRIzol Plus RNA Purification kit was purchased from Invitrogen (Carlsbad, CA, USA).

Ultrapure water was produced using a Milli Q water purification system manufactured by EMD Millipore Group (Darmstadt, Germany). The ultra-micro-spectrophotometer (K5800) and multiple microplate reader (K6600A) used in the experiments were manufactured by Beijing Kai’ao Technology Development Co., Ltd. (Beijing, China). All other reagents or instruments used in this study were made in China, unless otherwise stated.

### Animal experiments

Only male KDY rats and male WT rats were included in the animal experiments. A total of 33 WT rats and 125 KDY rats were included in the experiments. Among them, 10 WT rats were randomly arranged to record lifespan, and 23 WT rats were randomly arranged in five schedule groups (45, 180, 360, 540, and 626 days of age) to observe changes in their serum and organs. Seventy KDY rats were randomly used to record lifespan, and 55 KDY rats were randomly arranged in eight schedule groups (45, 60, 90, 180, 270, 360, 540, and 626 days of age) to observe changes in their serum and organs. Random integers ranging from 1 to 8 or 1–5 are used to determine which group the animals will be placed in. Every schedule group included three or more animals. The KDY and WT rats were fed normally under the same conditions. At the scheduled time points, the body weights of the rats were measured, and blood was collected to obtain serum. The animals were anesthetized with sodium pentobarbital (50 mg/kg) at the scheduled time points. When the animals were euthanized by dislocating their neck joints, the abdominal cavity was opened to obtain organs for the assay. The harvested organs were the kidney, liver, heart, duodenum, ileum, colon, spleen, and lung.

At the end of the experiment, the other animals alive were deeply anesthetized and sacrificed by neck dislocation. The corpses were placed in medical garbage bags and handed over to an environmental protection company for appropriate disposal. Animal experiments were approved by the Animal Care and Use Committee of Kunming Medical University (Approval No. KMMU-2020196) and were conducted following the Guidelines for the Ethical Review of Laboratory Animal Welfare of China (GB/T35, 892–2018). Animals were treated according to the ARRIVE guidelines (https://arriveguidelines.org).

### Blood biochemical assays

The animals were kept in a small box at 30 °C, the tail was exposed, the tip of the tail was cut off, and 200–500 μL of blood was collected. Blood was collected and centrifuged to prepare serum after coagulation by spinning at 5,000 × g for 5 min and at 4 °C. Serum was used for a variety of biochemical assays. The fresh tissue was cut into small pieces, and then was homogenized on ice at 3,000 rpm for 5 min using an electric homogenizer.

Substances in samples, including uric acid, glucose, LDL, HDL, TG, TC, Cr, BUN, D-lactate, LPS, insulin, glucagon, PTH, CRP, TAA, and HMGB1, were assayed using assay kits, the protocols of which can be downloaded at the Nanjing Jiancheng Bioengineering Institute (http://www.njjcbio.com/), Jiangsu Meimian Immunoassay Industry Co. Ltd. (http://www.mmbio.cn/) or Ruixin Biotech Co. Ltd. (http://www.ruixinbio.com) website by searching their name or lot number.

### Assay of uric acid in urine and feces

The urine and feces excreted by individual animals over 24 h were collected using metabolic cages. The volume of urine and the weight of feces were measured. The stirred urine was diluted 20 times with 50 mmol/L Tris solution, and the uric acid in it was assayed according to the kit protocol. The feces were added with three times its weight of 50 mmol/L Tris solution. The mixture was shaken on a shaker (120 rpm) for 2 h to extract the uric acid in the feces and then centrifuged at 10,000 × g for 5 min to obtain supernatant for uric acid assay.

### Tissue sections and staining

Animals were anesthetized, and their abdominal and thoracic cavities were opened. A rapid infusion of normal saline for injection was administered to the left ventricle at a pressure of 100 cmH_2_O. A hole was cut in the lung to release the perfusate. After the color of the discharged solution became light (approximately 50 mL of solution perfused), approximately 200 mL of 4% neutral formaldehyde solution was quickly perfused at the same pressure. Subsequently, the organs were removed and fixed in 4% neutral formaldehyde solution for more than 24 h for paraffin sectioning and staining.

Fixed tissues were dehydrated in ethanol, cleared in xylene to make them transparent, and embedded in paraffin. The paraffin-embedded tissues were sectioned at 5 μm thickness. Then, the sections were routinely stained using the HE staining kit or further stained using Masson’s staining kit to observe fibrosis. The stained sections were visualized and scanned using a fluorescence microscope in a light mode, and the images were read using CaseViewer software (Version: 2.4) (3DHISTECH Ltd, Budapest, Hungary).

### Abundance assay of expressed genes in organs

The experiments were performed by Sangon Biotech Co. Ltd (Shanghai, China, https://www.sangon.com/). Briefly, fresh organs were harvested as quickly as possible when the animals were anesthetized, then frozen with liquid nitrogen, and ground into powders. The total RNA in the powder was extracted and purified on ice using the TRIzol Plus RNA Purification kit. The quantity and quality of the extracted RNA were measured using an ultra-micro-spectrophotometer. The RNA integrity was assessed by its three bands (28S, 18S, and 5S) using formaldehyde denaturing agarose gel electrophoresis as previously described [16].

Similar to the methods of our previous study [17], double-stranded cDNA (ds-cDNA) was reverse transcribed from the total RNA using a SuperScript ds-cDNA synthesis kit (Invitrogen, Carlsbad, USA) in the presence of 100 pmol/L oligo dT primers. The Solexa high-throughput sequencing technique was used to sequence the cDNA. The raw data containing reads of 150 bases of nucleotide in fastq format were transformed to original sequences in fasta format by Seqkit software in the disc operation system (DOS) model. The sequences that map the rat’s reference mRNA sequences (https://www.ncbi.nlm.nih.gov/) were screened out using TBtools software (v0.664445552) [18]. The value of transcripts per kilobase million (TPM) [19,20] was used to normalize the expression level.

The relationship was correlated between ages (45, 185, 367, 549, and 626 days) and gene expression abundances in KDY or WT rats using the function of “CORRL(array1,array2)” of Excel software (19.0). Gene expression patterns were evaluated based on highly correlated (|r| > 0.7) genes expressed in an organ in both WT and KDY rats using Venn’s diagrams (https://bioinfogp.cnb.csic.es/tools/venny/index.html).

The TPM values of genes in every organ were calculated. The TPM value of every gene in the organ in KDY rats was statistically analyzed by comparing with that of WT rats to find the differential genes using Student’s t-test. Based on the differential genes, the Gene Ontology (GO) pathways (https://www.geneontology.org/) and Kyoto Encyclopedia of Genes and Genomes (KEGG) pathways (https://www.kegg.jp/) were enriched using clusterProfiler software (v3.0.5). The P and Q values were also calculated using the software. The common GO and KEGG pathway patterns among the organs of KDY rats were evaluated using Venn’s diagrams to identify significant pathways.

### Statistical analysis

All obtained data were included in the study. Values are expressed as the mean ± standard deviation (SD). The sample size for each group is ≥ 3. Because the weights of animals of the same age varied, the indices referring to total quantity were corrected by their body weight (200 g) to balance the difference caused by body weight. If the values exhibited a normal distribution according to the normality test (Shapiro-Wilk test), Student’s t-test was performed to compare the means between groups using the T-TEST function of Excel software (19.0); otherwise, a chi-test, or a nonparametric test (Mann-Whitney U Test) was applied using SPSS for Windows (version: 16.0). Statistical significance was set at P < 0.05.

## Results

### KDY rats showed retarded growth and shortened lifespan

**Fig 1:**
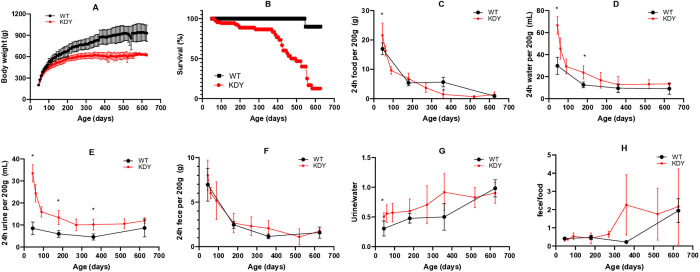
Basic observation of uricase-deficient rats (KDY rats) at 626 days of age (mean ± SD, n = 3–10). A, Body weight variation of male KDY rats; B, Survival rate of male KDY rats; C, Amount of food consumed by KDY rats in 24 h per 200 g body weight; D, Volume of water consumed by KDY rats in 24 h per 200 g body weight; E, Volume of urine excreted by KDY rats in 24 h per 200 g body weight; F, Amount of feces excreted by KDY rats in 24 h per 200 g body weight; G, Rate of urine/water calculated from D and E; H, The rate of feces/food calculated from C and F. WT, wild-type rats (Sprague-Dawley rats); * P < 0.05 vs WT of the same age.

Seventy male KDY rats and ten male WT rats were adopted for long-term observation (Fig 1A and B). The body weights of KDY rats were similar to those of WT rats when they were younger than 45 days of age. However, the body weights of KDY rats increased slowly afterward, and their body weights were significantly lighter than those of WT rats (Fig 1A). Nevertheless, a smaller variation in the body weights of KDY rats is shown Fig 1A. During the 626-day observation, approximately 90% KDY rats survived 360 days, and the survival rate dropped rapidly afterward (Fig 1B). At 626 days of age, only three KDY rats were still alive, and the observation had to be ended. The expected lifespan of KDY rats was approximately 450 days, while that of WT rats was more than 626 days.

At 45 days old or younger, KDY rats ate more food than WT rats, however, later, the amount of food they ate quickly decreased and was similar to that of WT rats (Fig 1C). However, KDY rats drunk more water, especially when they were 185 days old or younger (Fig 1D). At the same time, KDY rats excreted more urine than WT rats (Fig 1E), but a similar amount of feces (Fig 1F). Because the volume of urine is highly related to the water they drink, and the amount of feces is related to the food they eat, the rates of urine/water (mL/mL) and feces/food (g/g) were calculated. The rate of urine/water was higher in KDY rats than in WT rats, except at the end of observation (Fig 1G). At < 360 days of age, the rate of feces/food intake in KDY rats was similar to that in WT rats, and afterwards, the rate increased though without significance (Fig 1H).

In addition, four cases (4/70) of spontaneous osteoarticular gout were observed in KDY rats above 360 days of age. Among them, two cases occurred in the right ankle, one in the left ankle, and one in the caudal root, with swelling and/or ulceration, but no obvious infection.

### Uric acid in KDY rats

**Fig 2:**
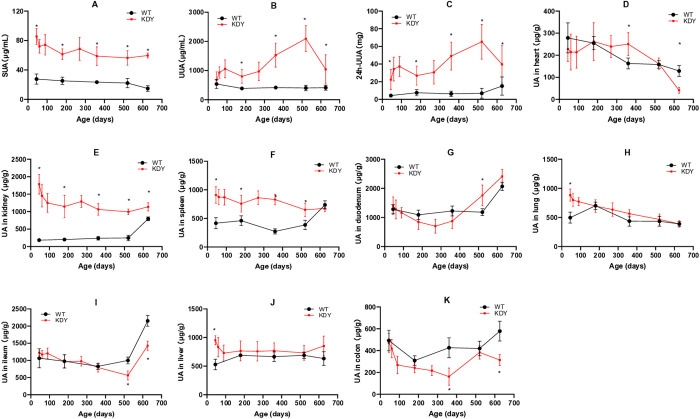
Uric acid in uricase-deficient rats (KDY rats) at 626 days of age (mean ± SD, n = 3–10). A, Serum uric acid (SUA); B, Uric acid in urine (UUA); C, Amount of uric acid in 24-h urine (24h-UUA); D, Uric acid in the heart; E, Uric acid in the kidney; F, Uric acid in the spleen; G, Uric acid in the duodenum; H, Uric acid in the lung; I, Uric acid in the ileum; J, Uric acid in the liver; K, Uric acid in the colon. WT, wild-type rats (Sprague-Dawley rats); *P < 0.05 vs WT of the same age.

The SUA levels in KDY rats were relatively stable and significantly higher than those in WT rats (Fig 2A), and approximately half of the KDY rats exceeded the diagnostic criteria for hyperuricemia (>70 μg/mL). The concentration of urine uric acid in KDY rats was similar to that in WT rats when the rats were 45 days of age, and later, the concentration increased and was significantly higher than that in WT rats (Fig 2B). Because of the increased volume of urine, the amount of 24-h urine uric acid significantly increased in KDY rats (Fig 2C).

Regarding uric acid in organs, the level of uric acid in the kidney in KDY rats was significantly higher than that in WT rats (Fig 2E). The uric acid level in the spleen of KDY rats was also higher than that in WT rats except at the end of the experiment (Fig 2F). The levels of uric acid in the heart (Fig 2D), duodenum (Fig 2G), lung (Fig 2H), ileum (Fig 2I), liver (Fig 2J), and colon (Fig 2K) of KDY rats varied to some extent.

### Glycolipid metabolism disorders in KDY rats

**Fig 3:**
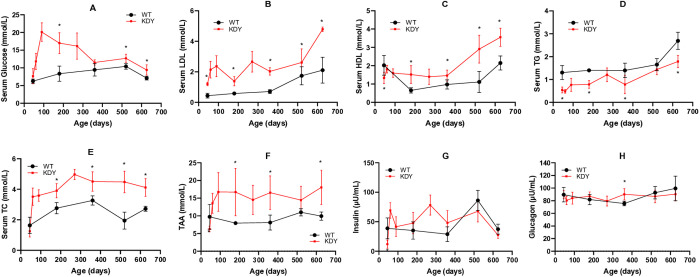
Serum metabolic indices in uricase-deficient rats (KDY rats) at 626 days of age (mean ± SD, n = 3 = 10). A, Serum glucose; B, Serum low density lipoprotein (LDL); C, Serum high-density lipoprotein (HDL); D, Serum triglyceride (TG); E, Serum total cholesterol (TC); F, Serum total amino acid (TAA); G, Serum insulin; H, Serum glucagon. WT, wild-type rats (Sprague-Dawley rats); * P < 0.05 vs WT of the same age.

Glycolipid metabolism disorders were found in KDY Rats. Serum glucose levels were stable in WT rats. In KDY rats, at 45 days of age the serum glucose level tended to increase but remained below the criteria of 11.1 mmol/L. However, the serum glucose level quickly and significantly increased later in KDY rats and could be used to diagnose diabetes mellitus. In KDY rats, the high serum glucose level was maintained until the end of the experiment, although it decreased to some extent afterward (Fig 3A).

The level of serum LDL in KDY rats increased from 45 days of age to the end of the experiment (Fig 3B). The level of HDL in KDY rats was similar to that in WT rats at 45 days of age, but it also increased significantly afterward (Fig 3C). Compared with WT rats, the level of TG in KDY rats decreased throughout their lifespan (Fig 3D). However, the level of TC in KDY rats was similar to that in WT rats at 45 days of age, but it significantly increased thereafter and was maintained (Fig 3E).

At 45 days of age, the level of serum TAA in KDY rats was similar to that in WT rats. Later, the level of TAA quickly increased until to the end of the experiment, although without significance (Fig 3F).

However, the levels of insulin (Fig 3G) and glucagon (Fig 3H) varied, and no increase or decrease in tendency was found.

### Renal function, intestinal barrier function and inflammatory indices in KDY rats

**Fig 4:**
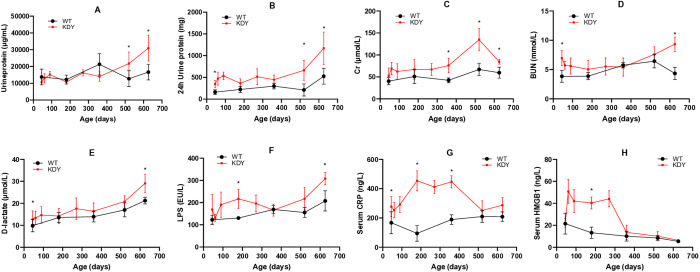
Indices of renal function, intestinal barrier function, and inflammation in KDY rats at 626 days of age (mean ± SD, n = 3–10). A, Level of urinary protein; B, Amount of protein in 24h urine; C, Serum creatine (Cr); D, Blood urea nitrogen (BUN); E, Serum D-lactate; F, Serum lipopolysaccharide (LPS); G, Serum C-reactive protein (CRP); H, Serum high mobility group box 1 (HMGB1). WT, wild-type rats (Sprague-Dawley rats); * P < 0.05 vs WT of the same age.

The indices, including urine volume, urinary protein, Cr, and BUN, are frequently used to evaluate renal function. As mentioned previously, the volume of urine in KDY rats increased (Fig 1E), which is usually an early sign of renal injury [21]. In addition, the urine protein concentration in KDY rats was similar to that in WT rats before they were 360 days old, and significantly increased when they were 549 days old or older (Fig 4A). When the total protein in the 24-h urine was calculated, KDY rats excreted more protein through urine (Fig 4B) because of the increased urine volume. The Cr level in KDY rats was similar to that in WT rats when they were 45 days old. However, the Cr level in KDY increased afterward, especially, when KDY rats were 360 days old or older (Fig 4C). Overall, the level of BUN was higher in KDY rats than in WT rats, although sometimes without significance (Fig 4D).

Serum D-lactate and LPS are the indices used to evaluate the intestinal barrier [22]. Overall, WT rats showed slight increase in serum D-lactate (Fig 4E) and LPS (Fig 4F), and the levels of serum D-lactate (Fig 4E) and LPS (Fig 4F) were higher in KDY rats than in WT rats, although sometimes without significance.

Serum CRP and HMGB1 are indices frequently used to evaluate inflammation [23]. The CRP level was higher in KDY rats than in WT rats before 360 days of age, before dropping slightly, although still remaining high (Fig 4G). However, the level of HMGB1 in KDY rats was similar to that in WT rats. The level quickly and significantly increased until the age of 360 days, and recovered (similar to that in WT rats) when they were older (Fig 4G-4H).

### Histological changes in KDY rats

Considering the high concentrations of uric acid are distributed in the kidney, liver, and intestines [24] and metabolic disorders attack the cardiovascular system [25], the histological changes in the kidney, liver, duodenum, colon and heart were next observed.

### Renal injuries in KDY rats

**Fig 5:**
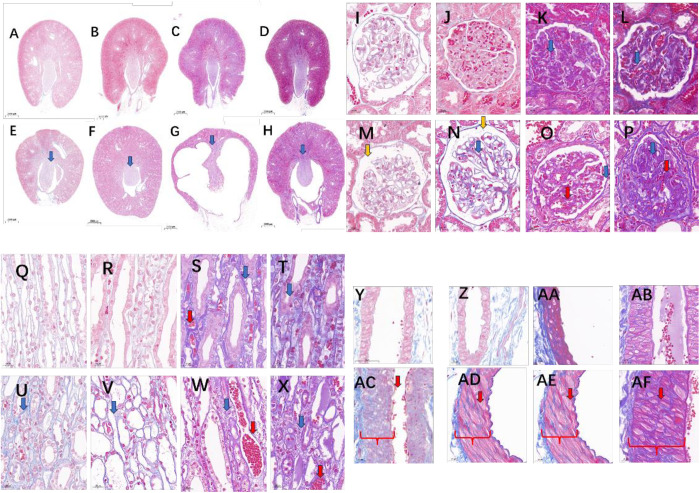
Renal injuries in uricase-deficient rats (KDY rats) (Masson’s staining). A-D, Whole kidney of WT rats at 185, 367, 549, and 626 days of age; E-H, Whole kidney of KDY rats at 185, 367, 549, and 626 days of age (blue arrows show the positive staining area with blue); I-L, Glomeruli of WT rats at 185, 367, 549, and 626 days of age (blue arrows show the fibrosis, and red arrows show the glomerular congestion); M-P, Glomeruli of KDY rats at 185, 367, 549, and 626 days of age (blue arrows showed the fibrosis, red arrows show the glomerular congestion, and yellow arrows showed the enlarged Bowman’s capsules); Q-T, Medullary collecting duct area of WT rats at 185, 367, 549, and 626 days of age; U-X, The medullary collecting duct area of KDY rats at 185, 367, 549, and 626 days of age (blue arrows show the fibrosis and red arrows show the glomerular congestion); Y-AB, Wall of the renal artery in WT rats at 45, 185, 367, and 626 days of age; AC-AF, Wall of the renal artery in KDY rats at 45, 185, 367, and 626 days of age (the red curly braces show the thickened artery wall, and the red arrows show the fibrosis).

The kidney outline in KDY rats became round with smooth edges (Fig 5E-5H), and the medulla of kidney had a tendency to become thinner with age, and was very thin when they were 549 days old or older leaving an obvious blank area in the medulla (Fig 5G-5H). Masson’s staining showed that, slowly progressive renal medullary fibrosis and cortical fibrosis developed in KDY rats compared with age-matched WT rats (Fig 5G-5H).

In KDY rats, glomerular fibrosis was not obvious at 185 days of age (Fig 5M), and the fibrosis slightly worsened at 367 days of age, and the glomerular capillaries and Bowman’s capsule expanded somewhat (Fig 5N). At 549 days of age (Fig 5O) or older (Fig 5P), glomerular fibrosis was much more obvious and the glomerular capillaries were congested in KDY rats.

Compared with age-matched WT rats (Fig 5Q-5T), medullary fibrosis was quite obvious in KDY rats at 185 days of age (Fig 5U), and widening of the renal tubules and collecting duct was also evident at 367 days of age, together with quite obvious fibrosis (Fig 5V). Later, medullary fibrosis and interstitial proliferation became more obvious, together with narrowed renal lumen and congestion (Fig 5U-5X).

In KDY rats, the thickened renal artery walls were worsened from 45 days of age to the end of life (Fig 5AC-5AF), with evidence of fibrous proliferation in the walls.

### Liver injuries in KDY rats

In WT rats, at 367 days of age or younger, glycogen granules could be observed around the nuclei of hepatocytes (S1A Fig-S1B), and glycogen loss occurred at 549 days of age or older (S1C Fig-S1D). Fat degeneration was observed in WT rats at 549 days of age (S1C Fig). In contrast, KDY rats showed the disappearance of glycogen and fat degeneration at 185 days of age and older, and the hepatocytes were much thronged.

### Intestinal injuries in KDY rats

Three parts of the intestine were observed: the start of the duodenum, the end of the ileum, and the starting of the colon.

Compared with WT rats, the injury of the duodenum was mild in KDY rats at 185 days of age (S1M Fig), but obvious mucosal exfoliation (S1N-S1P Fig) and even obvious inflammatory cell infiltration occurred in KDY rats at 367 days of age (S1Q Fig). Compared with WT rats, exfoliation of the ileum was obvious in KDY rats (S1U Fig-S1X).

Compared with WT rats, the injury to the colon was mild in KDY rats at 185 and 367 days of age (S1AC Fig–S1AD) but became obvious with inflammatory cell infiltration at 549 days of age or older (S1AE Fig–S1AF). Noted that the folds of the colon were also shorter in KDY rats (S1AE Fig–S1AF).

### Heart injuries in KDY rats

The inner surface of the heart chamber in KDY rats started bulging at 367 days of age or older (S2F Fig-S2G), though that in KDY rats at 185 days of age was similar to that in WT rats at the same age (S2E Fig). Especially, the walls of the left ventricle in KDY rats were thickened at 626 days of age (S2H Fig).

The cardiac muscle cells in KDY rats showed signs of hypertrophy, because the cardiac muscle cells were enlarged and the gaps between the cells were narrower than those in WT rats of the same age (S2M Fig-S2P). In addition, thrombosis was observed in the coronary artery of KDY rats at 626 days of age (S2P Fig).

When KDY rats were 549 days old or older, the intima of the coronary artery wall became rough (S2W Fig), and even vegetations grew at the intimal surface (S2X Fig).

### Genes expressed in organs of KDY rats showing a high correlation with age

**Fig 6:**
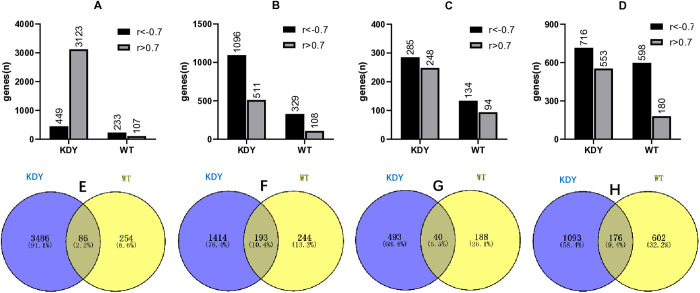
Genes that were highly correlated with age expressed in four investigated organs of KDY rats (n = 3). A-D, Number of highly correlated genes in the kidney (A), liver (B), duodenum (C), and ileum (D) in KDY and WT rats; E-H, Highly correlated genes in the kidney (E), liver (F), duodenum (G), and ileum (H) of both WT and KDY rats were analyzed using Venn’s diagrams.

As the kidney, liver, duodenum, and ileum are organs with higher uric acid distribution [15,24], gene expression in the four organs was next examined at the mRNA level at five time points (45, 185, 367, 549 and 626 days of age). The genes in the four organs that were highly correlated with age were screened out by an absolute correlation coefficient above 0.7 (|r| > 0.7). KDY rats had more highly positive and more negative correlation genes in the four organs than WT rats (Fig 6A-6D). In particular, the greatest number of positively correlated genes were observed in the kidneys of KDY rats (Fig 6A).

According to Venn’s diagrams (Fig 6E-6H), only a few genes highly correlated with age were present in the organs of both WT and KDY rats. In the kidney, only 2% of the highly correlated genes were present in both WT and KDY rats (Fig 6E).

### GO and KEGG pathways enriched in the organs of KDY rats

**Fig 7:**
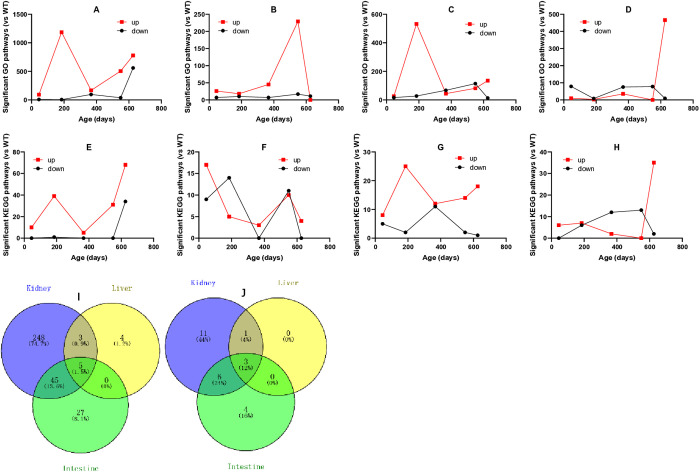
Number of significant GO and KEGG pathways with P and Q values below 0.05 enriched in uricase-deficient rats (KDY rats) vs age-matched wild-type (WT) rats. Number of significant GO pathways in the kidney (A), liver (B), duodenum (C), and ileum (D), respectively; Number of significant KEGG pathways in the kidney (E), liver (F), duodenum (G), and ileum (H), respectively; I, Venn’s diagram of GO pathways differentially expressed at three or more time points in KDY rats among the kidney, liver, and intestine (duodenum + ileum) [pathways coexisted in the kidney, liver and intestine are as follows: GO:0002474 (antigen processing and presentation of peptide antigen via MHC class I, biological process), GO:0019882 (antigen processing and presentation, biological process), GO:0048002 (antigen processing and presentation of peptide antigen, biological process), GO:0042605 (peptide antigen binding, molecular function), and GO:0042611 (MHC protein complex, cellular component)]; J, Venn’s diagram of KEGG pathways differentially expressed at three or more time points in KDY rats among the kidney, liver, and intestine (duodenum + ileum) [pathways coexisted in the kidney, liver and intestine are ko04145 (phagosome), ko04514 (cell adhesion molecules, CAMs), and ko04612 (antigen processing and presentation)].

Compared with age-matched WT rats, GO and KEGG signaling pathways were enriched in the kidney, liver, duodenum, and ileum of KDY rats based on the differential expressed genes. Pathways with P and Q values less than 0.05 were accepted as significant pathways. There were far more pathways upregulated than those downregulated in KDY rats. According to the results shown in Fig 7, most GO pathways significantly upregulated in the kidney were found in KDY rats at 185 days of age (Fig 7A), those in the duodenum were found at the same age (Fig 7C), those in the liver were found at 549 days of age (Fig 7B), and those in the ileum were found at 626 days of age (Fig 7D).

There were fewer significant KEGG pathways enriched than GO pathways (Fig 7E-7H). Nevertheless, except for the liver (Fig 7F), similar results of significant KEGG pathways were obtained in the kidney (Fig 7E), duodenum (Fig 7G), and ileum (Fig 7H).

The GO pathways that were differentially expressed in the four organs of KDY rats (vs WT rats), with P and Q values below 0.05 at five time points, are listed in Table 1, while the KEGG pathways that were differentially expressed at three or more time points are listed in Table 2. According to the results in Table 1, 31 GO pathways were found to be continuously differentially expressed during the lifespan of KDY rats (at five time points), and six and three pathways were in the duodenum and ileum, respectively. Unexpectedly, no liver pathways were differentially expressed throughout the lifespan of KDY rats (at five time points). According to the results in Table 2, a total of 21 KEGG pathways were differentially expressed at three or more time points in the kidney of KDY rats (vs WT rats), and four, eight, and ten pathways were differentially expressed in the liver, duodenum, and ileum, respectively. Notably, most enriched GO (Table 1) or KEGG pathways (Table 2) are associated with inflammation or immune responses.

**Table 1:** GO pathways continuously (at five time points) differentially expressed during the lifespan of KDY rats vs age-matched wild-type rats (n = 3).

GO.ID	Term	Ontology	45D	185D	367D	549D	626D	Count	organ
GO:0002252	immune effector process	biological process	6.10E-10	1.00E-21	1.10E-05	7.70E-25	2.60E-06	5	Kidney
GO:0002376	immune system process	biological process	1.10E-15	1.00E-30	1.70E-15	1.00E-30	7.50E-10	5	Kidney
GO:0002682	regulation of immune system process	biological process	3.80E-08	1.00E-30	2.40E-09	1.20E-30	4.20E-07	5	Kidney
GO:0002684	positive regulation of immune system process	biological process	1.90E-06	1.00E-30	5.20E-09	6.60E-23	4.50E-06	5	Kidney
GO:0005576	extracellular region	cellular component	2.50E-13	1.00E-30	7.00E-18	2.40E-26	1.50E-23	5	Kidney
GO:0005615	extracellular space	cellular component	4.00E-11	1.00E-30	1.50E-16	8.90E-28	2.90E-21	5	Kidney
GO:0006950	response to stress	biological process	6.60E-10	1.00E-30	2.10E-06	1.70E-22	1.90E-07	5	Kidney
GO:0006952	defense response	biological process	7.00E-12	2.40E-30	8.40E-12	1.00E-30	0.00039	5	Kidney
GO:0006955	immune response	biological process	8.70E-13	1.00E-30	4.20E-15	1.00E-30	7.50E-07	5	Kidney
GO:0007155	cell adhesion	biological process	5.60E-06	9.50E-24	5.20E-08	1.00E-06	7.30E-11	5	Kidney
GO:0007275	multicellular organism development	biological process	2.10E-05	3.20E-18	7.00E-05	2.00E-08	7.20E-20	5	Kidney
GO:0009605	response to external stimulus	biological process	6.30E-09	8.10E-28	3.50E-07	3.80E-15	1.90E-09	5	Kidney
GO:0009611	response to wounding	biological process	1.20E-08	3.50E-22	1.10E-05	7.70E-08	1.70E-10	5	Kidney
GO:0009986	cell surface	cellular component	0.00028	1.00E-30	1.20E-15	2.10E-17	8.40E-05	5	Kidney
GO:0022610	biological adhesion	biological process	7.20E-06	4.80E-24	3.50E-08	5.00E-07	1.70E-10	5	Kidney
GO:0031589	cell-substrate adhesion	biological process	2.40E-05	1.90E-07	4.70E-05	0.00071	8.80E-05	5	Kidney
GO:0032502	developmental process	biological process	7.60E-07	4.20E-21	1.70E-06	5.60E-08	7.60E-20	5	Kidney
GO:0042060	wound healing	biological process	1.70E-07	4.00E-16	1.40E-05	5.00E-07	6.10E-12	5	Kidney
GO:0043230	extracellular organelle	cellular component	1.00E-04	1.00E-30	5.20E-10	5.30E-15	3.90E-10	5	Kidney
GO:0044421	extracellular region part	cellular component	1.70E-12	1.00E-30	3.40E-19	1.40E-28	2.00E-19	5	Kidney
GO:0045087	innate immune response	biological process	2.30E-07	2.10E-18	8.20E-07	5.10E-20	0.00069	5	Kidney
GO:0048518	positive regulation of biological process	biological process	1.30E-06	1.00E-30	3.60E-08	7.40E-17	4.20E-25	5	Kidney
GO:0048522	positive regulation of cellular process	biological process	9.10E-05	4.60E-27	1.10E-06	1.30E-14	1.90E-25	5	Kidney
GO:0048583	regulation of response to stimulus	biological process	7.70E-05	1.00E-30	3.20E-05	2.00E-20	7.90E-21	5	Kidney
GO:0048584	positive regulation of response to stimulus	biological process	0.00016	1.00E-30	1.50E-06	3.40E-21	1.80E-14	5	Kidney
GO:0048731	system development	biological process	1.40E-05	4.30E-20	2.30E-05	4.60E-09	2.40E-20	5	Kidney
GO:0048856	anatomical structure development	biological process	5.00E-07	4.00E-20	1.10E-05	1.60E-08	5.20E-20	5	Kidney
GO:0050878	regulation of body fluid levels	biological process	2.80E-06	3.10E-09	0.00029	5.00E-05	3.90E-13	5	Kidney
GO:0070062	extracellular exosome	cellular component	0.00013	1.00E-30	5.80E-10	2.50E-14	2.50E-10	5	Kidney
GO:0072562	blood microparticle	cellular component	0.00012	0.00076	0.00055	7.10E-11	1.70E-21	5	Kidney
GO:1903561	extracellular vesicle	cellular component	9.40E-05	1.00E-30	8.70E-10	1.00E-14	3.50E-10	5	Kidney
GO:0002376	immune system process	biological process	5.30E-22	0.00045	8.20E-06	4.20E-21	2.70E-29	5	duodenum
GO:0003823	antigen binding	molecular function	5.30E-12	0.00029	3.30E-08	5.60E-12	4.10E-17	5	duodenum
GO:0005576	extracellular region	cellular component	1.70E-13	4.20E-05	4.20E-08	7.80E-18	7.30E-18	5	duodenum
GO:0005615	extracellular space	cellular component	3.40E-14	1.90E-08	1.10E-07	3.20E-16	3.20E-15	5	duodenum
GO:0006955	immune response	biological process	3.20E-23	0.00082	8.70E-05	5.50E-26	1.70E-30	5	duodenum
GO:0044421	extracellular region part	cellular component	2.90E-13	1.30E-07	2.60E-07	1.90E-16	1.30E-19	5	duodenum
GO:0005576	extracellular region	cellular component	2.20E-15	1.40E-06	3.40E-08	2.70E-10	1.00E-30	5	ileum
GO:0005615	extracellular space	cellular component	2.30E-16	1.10E-05	5.80E-06	4.20E-11	1.00E-30	5	ileum
GO:0044421	extracellular region part	cellular component	1.20E-15	1.10E-05	1.30E-07	4.10E-11	1.00E-30	5	ileum

Note: Red indicates upregulation vs. WT rats, and blue indicates downregulation.

**Table 2:** KEGG pathways differentially expressed at three or more time points in KDY rats vs. age-matched wild-type rats (n = 3).

id	Description	D45	D185	D367	D549	D626	Count	Organ
ko04610	Complement and coagulation cascades	4.54E-14	6.98E-05	2.00E-09	0.00132	1.31E-35	5	Kidney
ko04145	Phagosome	2.69E-06	4.56E-11	N/A	6.65E-22	7.87E-05	4	Kidney
ko04514	Cell adhesion molecules (CAMs)	9.28E-05	1.09E-13	N/A	1.35E-10	0.00753	4	Kidney
ko04612	Antigen processing and presentation	0.00331	3.10E-10	4.01E-05	3.30E-08	N/A	4	Kidney
ko04621	NOD-like receptor signaling pathway	N/A	0.00025	0.00095	1.53E-05	0.01653	4	Kidney
ko04650	Natural killer cell mediated cytotoxicity	0.00062	3.55E-10	N/A	1.37E-18	0.0016	4	Kidney
ko04668	TNF signaling pathway	N/A	3.58E-06	6.36E-05	0.00046	0.00031	4	Kidney
ko03010	Ribosome	N/A	2.31E-13	1.60E-29	1.23E-11	N/A	3	Kidney
ko04060	Cytokine-cytokine receptor interaction	0.00012	3.68E-08	N/A	3.79E-09	N/A	3	Kidney
ko04062	Chemokine signaling pathway	N/A	2.61E-08	N/A	1.79E-05	9.50E-05	3	Kidney
ko04064	NF-kappa B signaling pathway	N/A	2.60E-05	N/A	2.81E-13	5.06E-06	3	Kidney
ko04144	Endocytosis	N/A	2.65E-05	N/A	4.85E-05	0.00096	3	Kidney
ko04151	PI3K-Akt signaling pathway	N/A	0.00023	N/A	0.0002	5.33E-09	3	Kidney
ko04210	Apoptosis	N/A	0.00016	N/A	0.00049	0.00405	3	Kidney
ko04510	Focal adhesion	0.00251	3.27E-06	N/A	N/A	0.00078	3	Kidney
ko04512	ECM-receptor interaction	3.75E-07	1.15E-06	N/A	N/A	3.38E-06	3	Kidney
ko04611	Platelet activation	N/A	4.06E-06	N/A	0.00052	0.00026	3	Kidney
ko04640	Hematopoietic cell lineage	N/A	8.84E-09	N/A	5.34E-19	0.00038	3	Kidney
ko04662	B cell receptor signaling pathway	N/A	0.00017	N/A	1.49E-13	5.51E-06	3	Kidney
ko04664	Fc epsilon RI signaling pathway	N/A	0.00434	N/A	3.40E-10	0.00422	3	Kidney
ko04666	Fc gamma R-mediated phagocytosis	N/A	5.75E-05	N/A	5.17E-12	0.00055	3	Kidney
ko03010	Ribosome	2.21E-08	N/A	1.80E-20	1.91E-23	N/A	3	liver
ko04145	Phagosome	3.06E-07	3.54E-10	N/A	8.26E-06	N/A	3	liver
ko04514	Cell adhesion molecules (CAMs)	1.40E-07	2.11E-10	N/A	0.0003	N/A	3	liver
ko04612	Antigen processing and presentation	2.79E-10	6.18E-12	N/A	5.98E-09	N/A	3	liver
ko04145	Phagosome	5.78E-06	4.02E-06	0.00011	7.33E-06	1.07E-18	5	duodenum
ko04514	Cell adhesion molecules (CAMs)	0.00158	N/A	N/A	0.00107	1.54E-05	3	duodenum
ko04612	Antigen processing and presentation	0.00013	1.69E-12	0.00236	0.00068	1.29E-06	5	duodenum
ko04640	Hematopoietic cell lineage	N/A	N/A	7.29E-05	0.00028	2.76E-12	3	duodenum
ko04650	Natural killer cell mediated cytotoxicity	9.57E-07	N/A	0.00071	1.35E-06	2.03E-16	4	duodenum
ko04662	B cell receptor signaling pathway	N/A	N/A	0.0005	0.0001	1.93E-12	3	duodenum
ko04664	Fc epsilon RI signaling pathway	N/A	N/A	5.64E-05	0.00401	7.19E-08	3	duodenum
ko04672	Intestinal immune network for IgA production	N/A	N/A	5.33E-05	0.00095	9.48E-09	3	duodenum
ko04145	Phagosome	4.74E-06	5.67E-05	0.00102	6.15E-05	7.63E-25	5	ileum
ko04650	Natural killer cell mediated cytotoxicity	4.14E-05	0.00114	N/A	2.52E-05	1.83E-23	4	ileum
ko04151	PI3K-Akt signaling pathway	N/A	N/A	0.00446	0.00094	3.17E-22	3	ileum
ko04360	Axon guidance	N/A	N/A	0.00025	0.00373	0.00065	3	ileum
ko04514	Cell adhesion molecules (CAMs)	0.00266	N/A	0.00145	N/A	1.13E-08	3	ileum
ko04640	Hematopoietic cell lineage	N/A	N/A	0.00364	1.79E-05	3.35E-25	3	ileum
ko04666	Fc gamma R-mediated phagocytosis	N/A	N/A	0.00425	2.19E-05	5.09E-15	3	ileum
ko04672	Intestinal immune network for IgA production	N/A	N/A	0.0021	3.72E-08	1.80E-22	3	ileum
ko00601	Glycosphingolipid biosynthesis – lacto and neolacto series	N/A	0.0071	0.00388	N/A	0.00014	3	ileum
ko04020	Calcium signaling pathway	N/A	N/A	3.13E-05	1.89E-05	8.09E-19	3	ileum

Note: Red indicates upregulation vs. WT rats, and blue indicates downregulation; N/A indicates no significance.

If GO pathways with significant changes at three or more time points in organs were selected, and the pathways from the duodenum and ileum were pooled, five GO pathways that coexist in the kidney, liver, and intestine were identified using Venn’s diagram analysis (Fig 7I). The identified pathways included GO:0002474 (antigen processing and presentation of peptide antigen via MHC class I, biological process), GO:0019882 (antigen processing and presentation, biological process), GO:0048002 (antigen processing and presentation of peptide antigen, biological process), GO:0042605 (peptide antigen binding, molecular function), and GO:0042611 (MHC protein complex, cellular component). Among the five KO pathways, four are involved in immune responses, one is involved in the composition of immune molecular complexes, and all of them are associated with inflammation.

If the KEGG pathways from the duodenum and ileum were also pooled, three KEGG pathways that coexist in the kidney, liver, and intestine were identified using Venn’s diagram analysis (Fig 7J) based on the results in Table 2. The identified pathways included ko04145 (phagosome), ko04514 (cell adhesion molecules, CAMs), and ko04612 (antigen processing and presentation). All three pathways are involved in immune responses and are associated with inflammation.

## Discussion

Our previous studies have demonstrated that the SUA levels in KDY rats are stable at a high level, indicating that KDY rats are optimal animals for studying gout, hyperuricemia, and related disorders [15]. The present study systematically investigated the metabolic conditions throughout the lifespan of KDY rats, as well as the histological changes of the main organs and the mechanism at the transcriptional level. Our findings provide warning clues to the susceptibility of disorders in humans with uricase deficiency and provide a phenotypic basis for the application of KDY rats.

### Uricase deficiency is an important basis for hyperuricemia and abnormal glycolipid metabolism in KDY rats

In WT rats, spontaneous metabolic disorders and organ injury are relatively rare during the young-adult period (within 360 days of age) without dietary or drug intervention. However, due to uricase deficiency, KDY rats suffer metabolic disorders and organ injury in adulthood and even earlier.

In KDY rats, uricase deficiency can be regarded as a congenital event. The body weight did not differ significantly between KDY and WT animals from weaning (21 days of age) to adulthood (45 days of age). In adulthood and beyond, KDY rats showed a significant decrease in body weight (Fig 1A). Considering the normal reproductivity [14] and the rapid decline in survival of KDY rats after one year of age (Fig 1B), the abnormal serum indices and the tissue and organ injuries that occurred in older KDY rats are a result of the cumulative effect of uricase deficiency, in line with the characteristics of chronic diseases.

In line with a previous report [24], obvious polydipsia and polyuria also occurred much earlier in KDY rats (Fig 1D) and the water that was drunk mainly became urine (Fig 1E). Based on physiology theory, the behavior of water-drinking is usually triggered by an increase of the water requirement, which is often associated with an increase in blood osmotic pressure or a decrease in blood volume. Owing to the obvious renal medullary injury at the early stage in KDY rats (Fig 5U), the increase in water intake in KDY rats likely resulted from water loss via the kidneys.

In terms of blood metabolic indices, the elevated level of SUA occurred earlier and remained at a high state (Fig 2A), which is also the most important phenotype of KDY rats [14,15]. Following the elevated SUA levels in KDY rats, blood glucose (Fig 3A), TC (Fig 3E), and LDL (Fig 3B) also increased rapidly in the early stage (within 90 days of age), although TG (Fig 3D) decreased, indicating that these indices are more susceptible to the influence of the elevated SUA level. Serum TAA is an index reflecting the balance between protein degradation and synthesis. Because the weight loss in KDY rats was not obvious (Fig 3F) at 45 days of age or younger, the evidence that TAA decreased rather than increased at 45 days of age and subsequently increased in KDY rats supported the slowing of weight gain in KDY rats. Surprisingly, the levels of LDL (Fig 3B) and HDL (Fig 3C) both increased after 90 days of age in KDY rats. Considering that HDL increased slightly later than LDL, the increase of HDL may be a protective response of the body to the increased LDL [26].

Notably, no changes of the levels of serum insulin (Fig 3G) or glucagon (Fig 3H) were related to the level of blood glucose in KDY rats, although serum insulin and glucagon levels fluctuated to some extent. These phenomena are consistent with the characteristics of type 2 diabetes [27], indicating that KDY rats exhibit insulin resistance.

### Injuries in KDY rats

#### Renal injury in KDY rats.

Our previous studies have revealed that organs with higher tissue uric acid levels include the kidneys, liver, and small intestine (duodenum and ileum) [24]. Because the adrenal glands of KDY rats have a high level of uric acid, but lower than those of WT rats [15], the adrenal glands were excluded from this study. Due to the increased SUA level being closely related to cardiovascular diseases [28], the cardiovascular changes were also observed at the histological level in KDY rats.

As mentioned earlier, the increased SUA levels in KDY rats occurred very early and were maintained at a relatively high level throughout their lifespan. Compared with WT rats, the uric acid concentration in the urine of KDY rats showed a gradual increase to a peak and then decreased (Fig 2B), while the uric acid content in the kidneys remained at a relatively high level in early adulthood (Fig 2E). This suggests that the elevated level of SUA may be mainly deposited in the kidneys and is also the reason why gouty nephropathy is the first disorder found to be related to hyperuricemia.

It is necessary to discuss the pathogenesis of gouty nephropathy. Because the uric acid level is higher in the renal medulla, where uric acid or urate is prone to form microcrystals and stimulate inflammatory responses [29,30], is higher than in the renal cortex, it can be deduced that gouty nephropathy starts from the renal medulla, which is thronged with distal convoluted tubules and collecting ducts. Because the renal medulla mainly participates in water reabsorption rather than solute reclamation, KDY rats showed significant polyuria in the early stage (from 45 days of age) (Fig 1E), whereas survival could be maintained for one year or more (Fig 1B) by sufficient water intake (Fig 1D). In addition, fibrosis occurred in the medulla earlier than in the cortical glomeruli in KDY rats within 360 days of age (Fig 5U-5V), which is consistent with the view that the medulla is more prone to injury. However, long-term lesions of the medulla and distal convoluted tubules can lead to stenosis and occlusion of the tubules, followed by a drop in urine output (Fig 1E), and dilation of the proximal convoluted tubules and renal capsules (Fig 5N-5Q). Finally, when severe retrograde injury spreads to the proximal convoluted tubules and glomeruli, glomerular fibrosis becomes inevitable, leading to severe renal failure and a rapid drop in survival in KDY rats. Therefore, the early injury in KDY rats mainly starts in the renal medulla; then, 1 year later, the glomerulus (renal capsules) becomes injured (Fig 5Q), and renal failure soon occurs due to dramatic decrease in glomerular perfusion (Fig 4C).

Considering the increased volume of urine and the histological lesions of the kidney as early as 45 days of age in KDY rats [14,15], gouty nephropathy may start very early. Based on the abovementioned evidence and analysis, the accumulation of uric acid is key to kidney injury, although other factors such as abnormal glucose-lipid metabolism may also be involved.

#### Digestive system injury in KDY rats.

Similar to uricase-deficient mice [31], KDY rats also showed injuries in the digestive system [24]. In the livers of KDY rats, the main manifestations were hepatocyte enlargement, hepatic sinusoidal compression, and even hepatocyte steatosis (S1E Fig-S1H). The main manifestations in the small intestine of KDY rats were mucosal lesion (S1 Fig M-P, U-X, and AC-AF) and increased mucosal permeability (Fig 4E-4F). These changes are consistent with those observed in our earlier study on KDY rats [24]. However, in contrast to earlier study, a progressive injury was not observed in the digestive system of KDY rats as it occurred in the kidneys, because all of the injuries in the liver and intestine were similar to those in 45-day-old KDY rats [24]. Moreover, the levels of serum D-lactate and LPS, which are main indices to evaluate the permeability of the intestinal barrier, only slightly increased (Fig 4E-4F).

#### Cardiovascular system injury in KDY rats.

Surprisingly, the late stage KDY rats showed a significant increase in myocardial thickness (S2H Fig), a phenomenon that usually overcomes the increased afterload of myocardial contraction caused by elevated blood pressure, suggesting that KDY rats might eventually develop hypertension. The thickening of the renal artery wall (Fig 5AC-5AF) suggests that the kidneys may be the cause of the increased blood pressure in KDY rats. In addition, the atherosclerotic characteristics of the coronary artery intima were observed in KDY rats (S2 Fig W-X), which were related to the persistent elevation in TC (Fig 3E). Unfortunately, typical hyaline degeneration, which is often the most common pathological evidence of hypertension, was not found in the glomeruli or afferent arterioles of KDY rats, indicating that, by the time of death, KDY rats only experienced early-stage hypertension or a certain degree of elevated blood pressure, without typical pathological evidence.

### Immune and inflammatory responses in systemic tissue and organ injuries caused by metabolic disorders

The anti-injury response is an inevitable response of the body to injury, manifested as inflammatory or immune responses. Elevated serum CRP and HMGB1 levels are non-characteristic markers of inflammatory responses [23]. Among them, CRP is associated with acute inflammation [32], and HMGB1 with chronic inflammation [33]. Because the level of serum CRP in KDY rats began to increase as early as at 45 days of age, or even earlier (Fig 4G), and given that serum CRP and HMGB1 increased and were maintained from 60 to 360 days of age (Fig 4H), it is deduced that acute inflammation occurs in young KDY rats and subsequently leads to a continuous elevation of CRP and HMGB1 because of persistent and unremovable factor(s), including the elevated level of SUA.

The results of gene expression analysis (Fig 6) indicated that KDY rats had a unique gene expression pattern that was quite different from that of WT rats. Based on the results of GO and KEGG pathway enrichment (Fig 7), most age-specific and differentially expressed pathways were related to inflammatory (or immune) responses (Tables 1 and 2). Unsurprisingly, common GO or KEGG pathways involved in immune or inflammatory responses were found in the liver, kidney and intestine of KDY rats. Therefore, inflammatory (or immune) responses run through the entire lifespan of KDY rats and could be the direct cause of systemic tissue and organ injury.

The upregulation of NLRP3 pathway, which was verified in uricase-deficient mice [34] and uricase-deficient rats [35] (on background of Wistar rats), was not enriched in the present study. However, the present study found that NLRP3, playing a vital role in the pathway, was upregulated at mRNA level in the kidneys of KDY rats when the animals were 185 days old or older (S1 Table), supporting the upregulation of NLRP3 pathway.

### Outstanding issues to be addressed

This study systematically observed the abnormal metabolic patterns of uric acid and glycolipids in KDY rats, as well as the patterns of injury in the main organs. However, this study still has certain limitations. For example, the indication that KDY rats have hypertension (rather than elevated blood pressure) requires further functional evidence. Additionally, due to the different animal sources, it was impossible to execute absolute random grouping in this study to observe the differences between KDY and SD rats. Because the lifespan of KDY rats is approximately 450 days, which is much shorter than that of SD rats (>626 days), there may be a serious “survivor bias” in the observation indicators of KDY rats older than 450 days of age.

**Fig 8:**
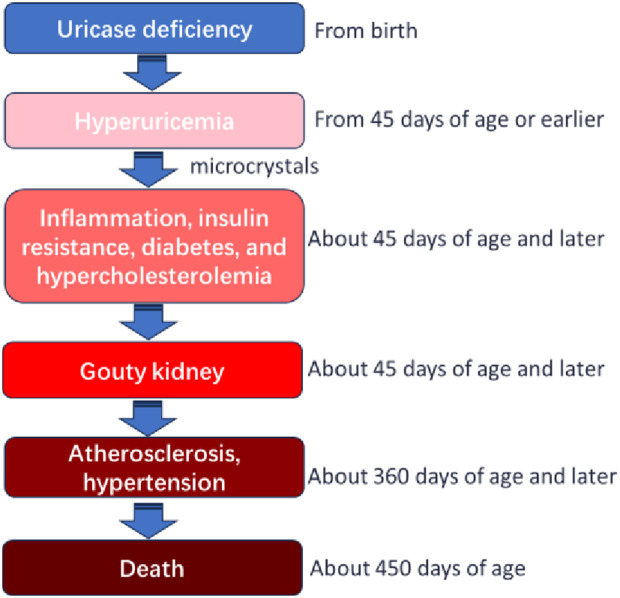
Life history of KDY rats.

In addition, it is worth noting that, based on the existing knowledge, the present study supports the pathogenic route of “uricase deficiency -> hyperuricemia -> inflammation -> glycolipid metabolism disorder (insulin resistance) -> multi-organ injury (including kidneys) -> death” (Fig 8). However, the causal relationship of this route requires further investigation.

## Conclusions

Here, male KDY rats were observed for nearly 2 years and found that, compared with SD rats, the animals are prone to developing hyperglycemia and hyperlipidemia and even show a tendency to develop hyperuricemia, gouty nephropathy, diabetes mellitus (hyperglycemia), atherosclerosis, and hypertension.

## Supporting information

S1 Fig Liver and intestine injuries in uricase-deficient rats (KDY rats) (HE staining). A-D, Liver of WT rats at 185, 367, 549, and 626 days of age; E-H, Liver of KDY rats 185, 367, 549, and 626 days of age (HE staining; red arrows show the fat degeneration); I-L, Duodenum of WT rats at 185, 367, 549, and 626 days of age; M-P, The duodenum of KDY rats at 185, 367, 549, and 626 days of age (blue arrows show the exfoliation of mucosa and the red sowed the infiltrated inflammatory cells); Q-T, Ileum of WT rats at 185, 367, 549, and 626 days of age; U-X, Ileum of KDY rats at 185, 367, 549, and 626 days of age (blue arrows show the exfoliation of mucosa); Y-AB, Colon of WT rats at 185, 367, 549, and 626 days of age; AC-AF, Colon of KDY rats 185, 367, 549, and 626 days of age (blue arrows show the exfoliation of the mucosa and red arrows show the infiltrated inflammatory cells).
(TIF)

S2 Fig Heart injuries in uricase-deficient rats (KDY rats) (HE staining). A-D, Whole heart of WT rats at 180, 360, 540, and 626 days of age; E-H, Whole heart of KDY rats at 185, 367, 549, and 626 days of age (red arrows show the bulges in the inner surface of chamber, and blue arrow shows the thickened wall of the left ventricle); I-L, Cardiac muscle tissue of WT rats at 180, 360, 540, and 626 days of age; M-P, Cardiac muscle tissue of KDY rats at 185, 367, 549, and 626 days of age (red arrows show the enlarged cardiac muscle cells, and blue arrow shows the thrombosis); Q-T, Coronary artery wall of WT rats at 185, 367, 549, and 626 days of age; U-X, Coronary artery wall of KDY rats at 185, 367, 549, and 626 days of age (red arrows show the rough intima of the coronary artery, and blue dotted area shows the vegetations in the intima).
(TIF)

S1 Table NLRP3 expressed at mRNA level in the kidney, liver, duodenum, and ileum. (XLSX)

S3 The supporting information are uploaded in the submission system, including dataset protocols (Protocols626. (ZIP)

S4 The supporting information are uploaded in the submission system, including dataset 626 Raw data. (ZIP)
