# Supplementary figures and images for: Uricase deficiency in rats results in a variety of metabolic disorders, addition to gouty nephropathy
Source: PLoS One. 2025 Aug 22;20(8):e0330344. doi: 10.1371/journal.pone.0330344 (PMC12373213; doi:10.1371/journal.pone.0330344)

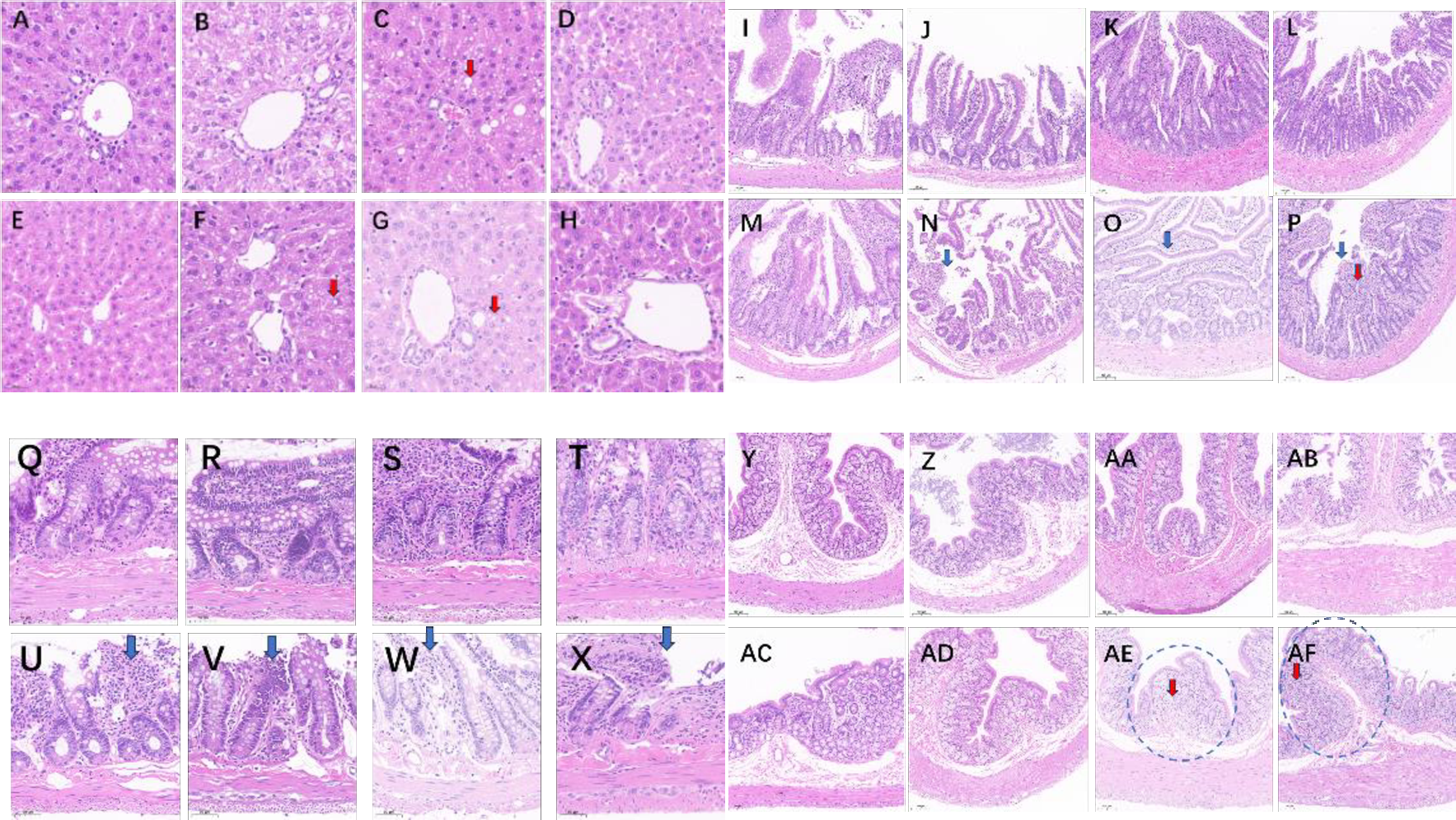

Supplement: S1 Fig — A-D, Liver of WT rats at 185, 367, 549, and 626 days of age; E-H, Liver of KDY rats 185, 367, 549, and 626 days of age (HE staining; red arrows show the fat degeneration); I-L, Duodenum of WT rats at 185, 367, 549, and 626 days of age; M-P, The duodenum of KDY rats at 185, 367, 549, and 626 days of age (blue arrows show the exfoliation of mucosa and the red sowed the infiltrated inflammatory cells); Q-T, Ileum of WT rats at 185, 367, 549, and 626 days of age; U-X, Ileum of KDY rats at 185, 367, 549, and 626 days of age (blue arrows show the exfoliation of mucosa); Y-AB, Colon of WT rats at 185, 367, 549, and 626 days of age; AC-AF, Colon of KDY rats 185, 367, 549, and 626 days of age (blue arrows show the exfoliation of the mucosa and red arrows show the infiltrated inflammatory cells). (TIF) [file pone.0330344.s001.tif]

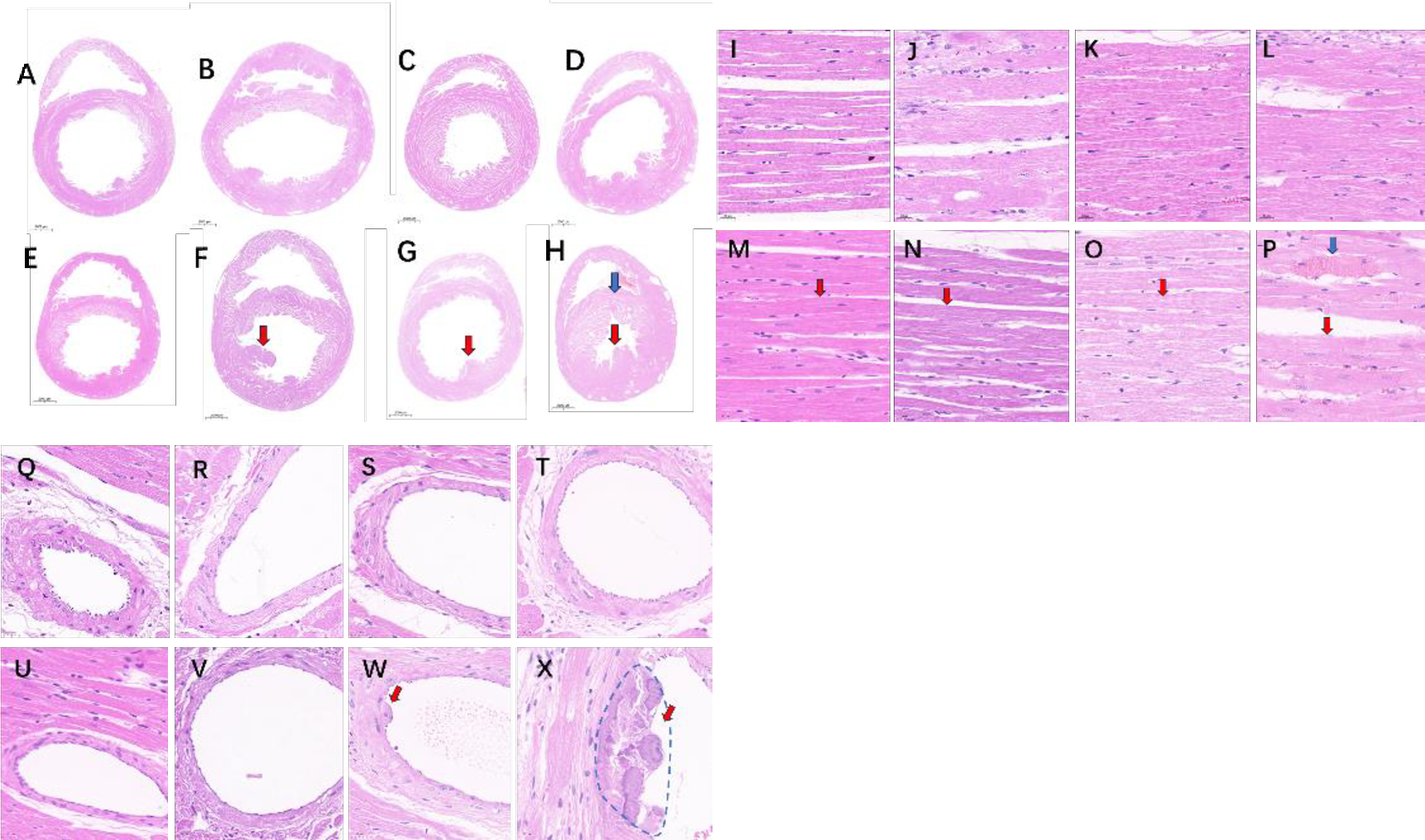

Supplement: S2 Fig — A-D, Whole heart of WT rats at 180, 360, 540, and 626 days of age; E-H, Whole heart of KDY rats at 185, 367, 549, and 626 days of age (red arrows show the bulges in the inner surface of chamber, and blue arrow shows the thickened wall of the left ventricle); I-L, Cardiac muscle tissue of WT rats at 180, 360, 540, and 626 days of age; M-P, Cardiac muscle tissue of KDY rats at 185, 367, 549, and 626 days of age (red arrows show the enlarged cardiac muscle cells, and blue arrow shows the thrombosis); Q-T, Coronary artery wall of WT rats at 185, 367, 549, and 626 days of age; U-X, Coronary artery wall of KDY rats at 185, 367, 549, and 626 days of age (red arrows show the rough intima of the coronary artery, and blue dotted area shows the vegetations in the intima). (TIF) [file pone.0330344.s002.tif]

## Slide 1
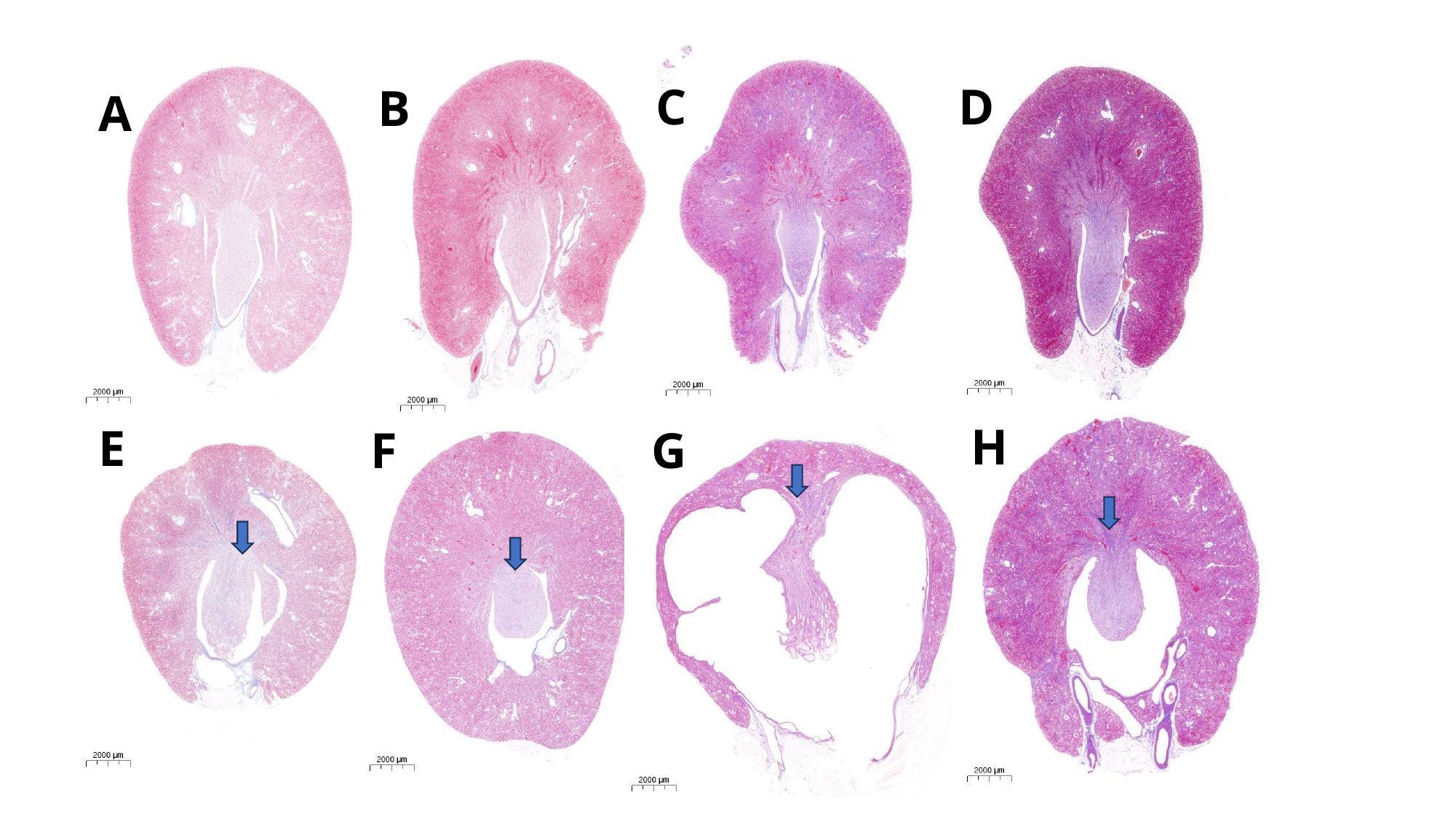

D
C
B
A
H
E
F
G

## Slide 2
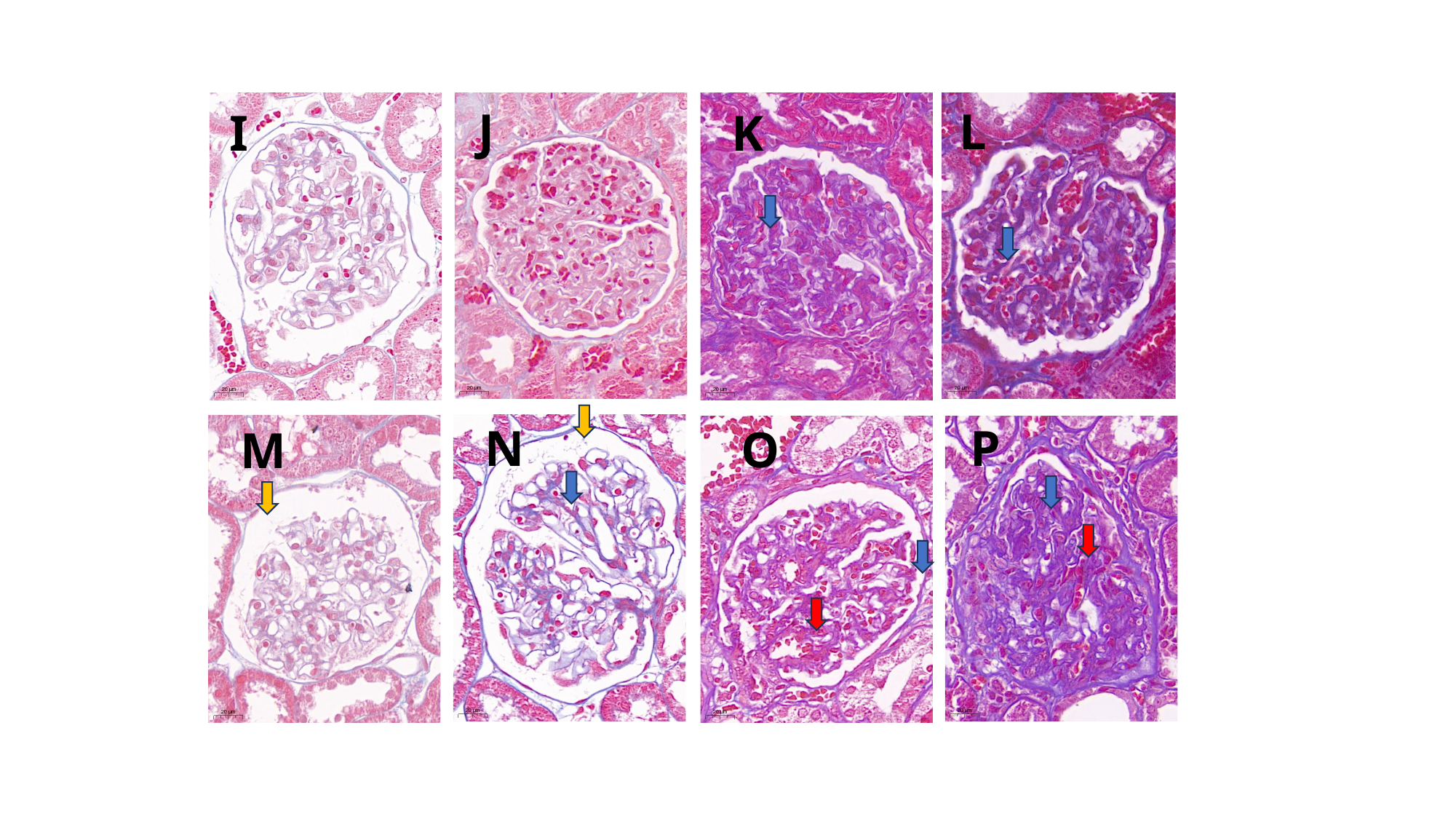

J
L
K
I
N
P
O
M

## Slide 3
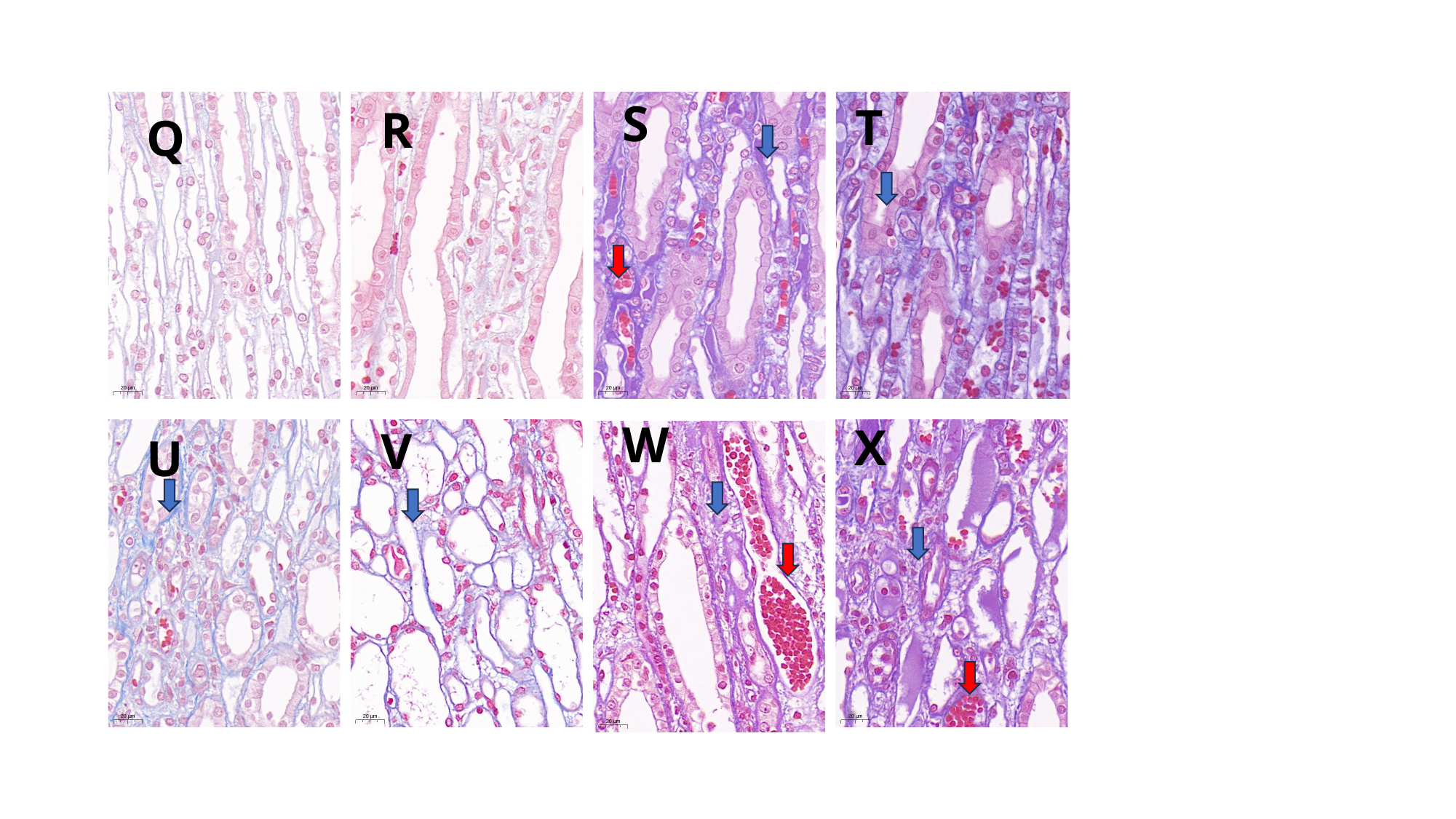

S
T
R
Q
W
X
V
U

## Slide 4
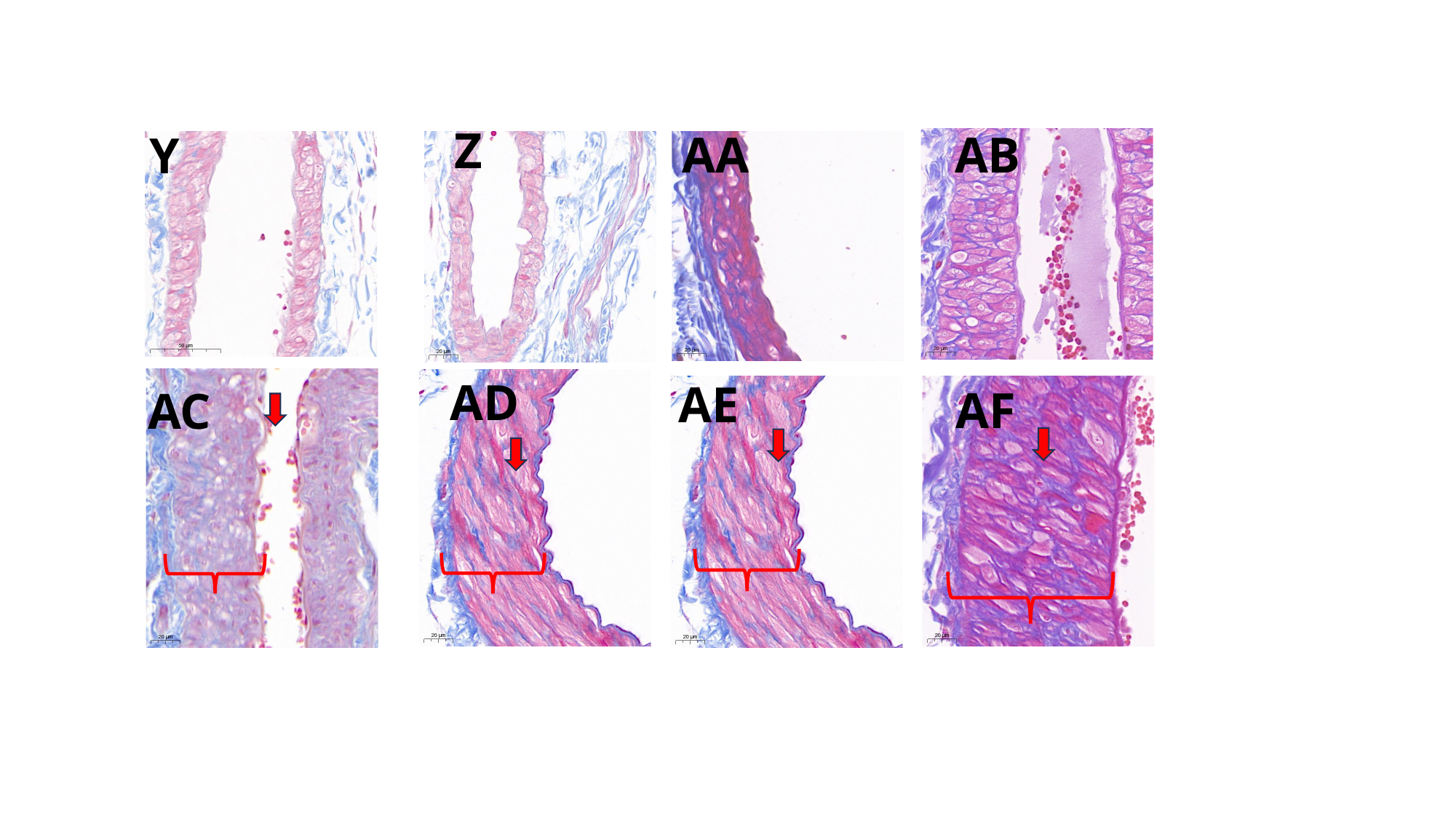

Z
AA
AB
Y
AD
AE
AF
AC

Supplement: S4 — (ZIP) [file pone.0330344.s005.zip › Figure5 photo.pptx]
