# Supplementary material for: Uricase deficiency in rats results in a variety of metabolic disorders, addition to gouty nephropathy
Source: PLoS One. 2025 Aug 22;20(8):e0330344. doi: 10.1371/journal.pone.0330344 (PMC12373213; doi:10.1371/journal.pone.0330344)
Supplement: S3 — (ZIP) [file pone.0330344.s004.zip › BUN.pdf]

血尿素氮(BUN)测试盒说明书(精简版)

（货号：C013-1-1　　二乙酰肟比色法　　100 管/96 样）

免责声明：测试前请仔细阅读说明书,预试后再进行批量实验,否则由此导致的后果用户自行承担!

一、测定原理：

在加热和强酸条件下，尿素氮与二乙酰肟缩合成红色的联吖嗪称之 Fearon 反应。根据色泽的深浅可以计算出尿素氮的含量。

二、标本：

草酸盐、肝素或 EDTA 抗凝的血浆。血浆中的尿素氮在室温下可稳定 24 小时，而在 4～6℃至少稳定 7 天。尿液用生理盐水作 1:10～1:50 稀释后与血浆操作相同。若超出线性范围，须再稀释。

三、所需仪器及试剂：

可调 520nm 波长的可见光分光光度计及 1cm 光径比色皿(或酶标仪及 96 孔板)，蒸馏水，涡旋混匀器，沸水浴锅。

三、试剂的组成：（试剂盒有效期一年）

试剂一：1g/L 的肟溶液 100mL×1 瓶，4℃保存。

试剂二：酸溶液 40mL×1 瓶，用时加双蒸水 80mL 配成酸应用液，4℃保存。

试剂三：10mmol/L 尿素氮标准品×1 瓶，4℃保存。

四、操作表：

	空白管	标准管	测定管
双蒸水（mL）	0.02		
10mmol/L 尿素氮标准品（mL）		0.02	
待测样本（mL）			0.02
试剂一（mL）	1	1	1
试剂二应用液（mL）	1	1	1
混匀，置沸水中准确水浴 15 分钟，取出用自来水冷却，混匀，于波长 520nm，测定各管吸光值 A			

五、计算公式：

$$\text{BUN含量}(\text{mmol/L}) = \frac{A_{测定} - A_{空白}}{A_{标准} - A_{空白}} \times C_{标准} \times N$$

$C_{标准}$:标准品浓度,10mmol/L(280.1mg/L);
N:样本测试前稀释倍数。

六、注意事项：

1、酸溶液与肟溶液可按等量混匀，用量为 2mL，但此混合液只能保存 7 天左右。

2、比色前若发现沉淀，则可 3500 转/分离心 10 分钟。

3、测定 OD 过高时(大于 0.8)，将样品作适当稀释，结果再乘以稀释倍数。

4、重度脂血标本要用除蛋白滤液测定。

5、试剂 4℃保存，有效期一年。

6、本法不但能用分光光度计读数，也可以在反应完后从反应液中吸取适量（200-300μL），加入到 96 孔板中（注意不要引入气泡），酶标仪 520nm 下读数，吸光值代入公式计算。

附录：尿素氮标准曲线制作

1、前处理：

取 20mmol/L 尿素氮标准品用双蒸水稀释成不同浓度：20mmol/L、15mmol/L、10mmol/L、8mmol/L、5mmol/L、4mmol/L、2mmol/L、1mmol/L、0.5mmol/L 用于制作标准曲线。

2、操作表：

	空白管	标准管
双蒸水（mL）	0.02	
不同浓度尿素氮标准品（mL）		0.02
试剂一（mL）	1	1
试剂二应用液（mL）	1	1
混匀，置沸水中准确水浴 15 分钟，立即用自来水冷却,于波长 520nm，1cm 光径，双蒸水调零，分光光度计测定各管吸光值 A		

3、所得结果绘图如下：

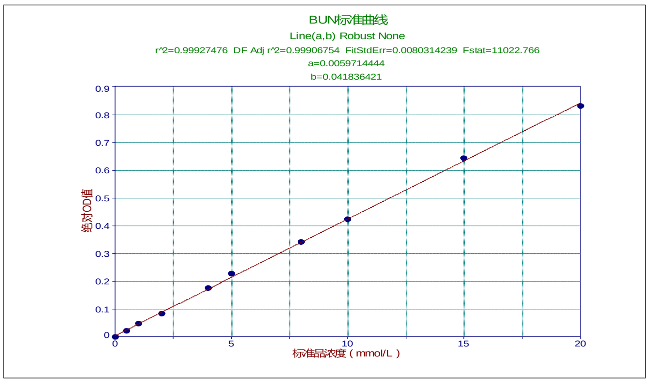

注:上述标准曲线用户可以不做,只需按前面的操作表测定,按公式计算即可,结果不受影响。
