# Supplementary material for: Uricase deficiency in rats results in a variety of metabolic disorders, addition to gouty nephropathy
Source: PLoS One. 2025 Aug 22;20(8):e0330344. doi: 10.1371/journal.pone.0330344 (PMC12373213; doi:10.1371/journal.pone.0330344)
Supplement: S3 — (ZIP) [file pone.0330344.s004.zip › Cr.pdf]

肌酐（CRE）测定试剂盒说明书(精简版)

（货号：C011-2-1　肌氨酸氧化酶法　　微板法　96T）

免责声明：测试前请仔细阅读说明书,预试后再进行批量实验,否则由此导致的后果用户自行承担!

【试剂组成】

试剂名称	规格装量	保存条件
试剂一（R1）：酶溶液 A	18mL	4℃避光
试剂二（R2）：酶溶液 B	6mL	4℃避光
试剂三：标准品（442μmol/L）	100μL	4℃
96 孔平底酶标板	一块	室温

【检验原理】

肌酐(Creatinine)在肌酐酰胺水解酶的催化下生成肌酸，肌酸在肌酸胺基水解酶的催化下水解成肌氨酸和尿素，肌氨酸再经肌氨酸氧化酶催化生成甘氨酸、甲醛和过氧化氢。过氧化氢与 2，4-（6-三碘-3-羟基苯甲酸）及 4-氨基安替比林在过氧化物酶的催化下反应生成紫红色化合物。可通过 546nm 波长比色测定。

$$\text{肌酐}+\text{水}\xrightarrow{\text{肌酐酰胺水解酶}}\text{肌酸}$$

$$\text{肌酸}+H_2O+O_2\xrightarrow{\text{肌酸胺基水解酶}}\text{肌氨酸}+\text{尿素}$$

$$\text{肌氨酸}+H_2O+O_2\xrightarrow{\text{肌氨酸氧化酶}}\text{甘氨酸}+HCHO+H_2O_2$$

$$2H_2O_2+4\text{-氨基安替比林}+2,4\text{-}DCP\xrightarrow{\text{过氧化物酶}}\text{醌亚胺}+4H_2O$$

【储存条件及有效期】

试剂盒 2～8℃保存，有效期 1 年。

【所需仪器及试剂】

可调 546nm 波长的酶标仪，37℃水浴锅或恒温箱，蒸馏水，生理盐水。

【操作步骤】

孔别 / 加入物	测定（T）	标准（S）	空白（B）
样本（μL）	6		
试剂三：标准品（μL）		6	
蒸馏水（μL）			6
试剂一：酶溶液 A（μL）	180	180	180
37℃孵育 5 分钟，546nm 波长测定吸光度值 A1			
试剂二：酶溶液 B（μL）	60	60	60
37℃孵育 5 分钟，546nm 波长测定吸光度值 A2,计算△A=A2－**K***A1			

注：测定前先将孔板在 546nm 处读出其空板 OD 值,后面实验结束后计算时，A1 和 A2 值均需减去对应孔的空板 OD 值后才能代入计算公式；K 为稀释因子,数值为:

$$K=\frac{\text{加样量}+\text{酶溶液A体积}}{\text{加样量}+\text{酶溶液A体积}+\text{酶溶液B体积}}=\frac{186}{246}$$

【计算公式】

$$\text{肌酐含量（μmol/L）}=\frac{\Delta A_{\text{测定}}-\Delta A_{\text{空白}}}{\Delta A_{\text{标准}}-\Delta A_{\text{空白}}}\times C_{\text{标准}}$$

$C_{\text{标准}}$:标准品浓度，442μmol/L。

【测定意义】

本试剂盒用于血清、血浆或尿中肌酐含量的测定。肌酐是由肌酸脱去一分子水缩合而成的一种环状结构。形成后的肌酐基本上通过肾脏排出体外，一般情况下血清或血浆肌酐浓度的测定是使用最广泛的肾功能试验。肌酐是在肌肉中从磷酸肌酸通过自发和不可逆转化而形成的，除非肌肉质量有大的变化，通常情况所形成的肌酐量是相当恒定的。游离肌酐的循环量完全依赖于它的排泄速度，从而测定血清或血浆中的肌酐量，可用于肾功能检查。肌酐含量的增高见于：慢性肾衰竭时排泄量的减少及肢端肥大症。可用于评价肾小球滤过率，以确定肾功能状态。

【注意事项】

① 在测定尿液样本之前，请用生理盐水将样本稀释 2～10 倍,结果乘以稀释倍数。
② 试剂二：酶溶液 B 中加入了防腐剂叠氮化钠，如该物质接触到了皮肤，请立即用水充分冲洗。
③ 样品与试剂比例可根据需要按比例调节。
④ 不同批次的试剂不推荐混合使用。
⑤ 仅用于科研，不用于体外诊断。
⑥ 检测范围:5-2000μmol/L。

公司地址：南京市玄武区中央路 258-27 号新立基大厦　邮政编码：210009　E-Mail：njjcbio@vip.163.com
联系电话：025-83360321、83360969、83551389　技术支持：025-83360272、19951670086
