# Supplementary material for: Uricase deficiency in rats results in a variety of metabolic disorders, addition to gouty nephropathy
Source: PLoS One. 2025 Aug 22;20(8):e0330344. doi: 10.1371/journal.pone.0330344 (PMC12373213; doi:10.1371/journal.pone.0330344)
Supplement: S3 — (ZIP) [file pone.0330344.s004.zip › D-Lac.pdf]

D-乳酸（D-Lac）测试盒说明书

（货号：A019-3-1　比色法　50 管/48 样）

免责声明：测试前请仔细阅读说明书,预试后再进行批量实验,否则由此导致的后果用户自行承担!

一、测定原理：

D-乳酸在 D-乳酸脱氢酶的作用下生成丙酮酸,并使 NAD^+还原生成 NADH（为使该反应顺利进行另添加酶进一步分解丙酮酸），生成的 NADH 与特异显色剂反应产生有色物质，该有色物质在 450nm 处有最大吸收峰，通过检测该有色物质的生成量，可计算出 D-L 乳酸含量。

二、试剂组成及配制：(试剂盒有效期 6 个月)

试剂名称	规格	保存条件
提取液	液体 60mL×1 瓶	4℃
试剂一	粉剂×1 支	4℃
	用前甩几下或离心使试剂落入底部，再加 2.1mL 试剂三溶解备用，用不完的 4℃保存。	
试剂二	液体 1.1mL×1 支	4℃避光
试剂三	液体 30mL×1 瓶	4℃
试剂四	液体 1mL×1 支	4℃
试剂五	浓缩液×2 支	-20℃
	用前甩几下或离心使试剂落入底部，每支加 0.55mL 蒸馏水稀释备用，用不完的-20℃保存（避免反复冻融）。	
标准品	标准母液×1 支	4℃

三、所需仪器及试剂：

可调 450nm 波长的分光光度计及 1ml（1cm 光径）比色皿（或酶标仪及 96 孔板），涡旋混匀器，37℃水浴锅或恒温箱，蒸馏水，蛋白测定试剂（组织或细胞用，本公司有售）。

四、操作步骤：（正式实验前请选取 2 例样本进行预试，摸索最佳样本浓度，并了解实验流程）

1、样本前处理：

① **血清（浆）样本：**可直接使用（样本尽量澄清）；

② **组织样本：**称重（约 0.05-0.1g），按重量（g）体积（mL）比为 1:10（含量低时可按 1:5 制备）的比例加入提取液，4℃研磨匀浆，8000-12000 转/分离心 10 分钟，取上清待测（上清需测定其蛋白浓度）；

③ **细菌/细胞样本：**收集细菌或细胞到离心管中（注意去除培养液），每 500 万细菌或细胞可加入 0.5mL 提取液，超声波碎（冰浴，功率 20%或 200W，运行 5 秒，间隔 15 秒，重复 5-10 次），8000-12000 转/分离心 10 分钟，取上清待测（上清需测定其蛋白浓度）；

④ **其它液体样本：a、近似中性的液体样本**可直接 8000-12000 转/分离心 10 分钟，取上清待测；b、**酸性液体样本**需先用 KOH（5mol/L）调溶液 pH 值至 8 左右（注意计算最终体积与初始体积的比，即样本被稀释的倍数），充分混匀，室温静置 30 分钟后 8000-12000 转/分离心 10 分钟，取上清待测。

2、操作表：（试剂一、二、三、四可预先按所加比例混合配制好后一次性加入反应。所有试剂取出恢复至室温再用）

	空白管	测定管	标准管
样本（μL）	-	60	-
不同浓度标准品（μL）	-	-	60
试剂一（μL）	40	40	40
试剂二（μL）	20	20	20
试剂三（μL）	600	540	540
试剂四（μL）	20	20	20
试剂五（μL）	20	20	20
混匀，37℃避光反应 30 分钟,调仪器波长 450nm，读取各管吸光值 A，$\triangle A=A_{测定}-A_{空白}$。			

【注】1、标准管用于制作标准曲线用，标准品稀释为：取标准品母液 3μL，加 1mL 蒸馏水稀释即为 30μmol/mL 标准液，再取 30μmol/mL 标准液用蒸馏水分别稀释至 0、0.06、0.12、0.18、0.24、0.3μmol/mL 几个浓度（此标曲各个浓度可根据需要调整，上限 0.5μmol/mL）按操作表标准管操作，所得吸光值统一减去 0 浓度管吸光值后（即为△A）对应标准品摩尔质量（标准品加入量×标准品浓度）作标准曲线。

2、若样本有很强的背景值（如颜色很深或含有还原性物质如抗坏血酸等），可以加设一个样本自身对照（即试剂五用蒸馏水代替，其它试剂保持不变，则$\triangle A=A_{测定}-A_{对照}$，且试剂盒能测的样本数也会减少）。

3、若△A 值较小（如小于 0.01），则可增加样本上样量 V_1（如 100μL，则试剂三量相应减少）；若△A 值较大（如大于 0.8 或超过标曲最高点），则需将样本稀释后测定（稀释倍数代入计算公式计算）。

五、计算：

1、标准曲线为：y=39.127x-0.0101；x 为标准品摩尔质量(μmol)，y 为△A。

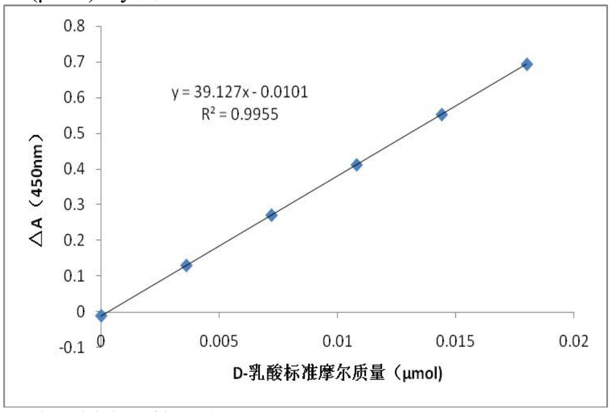

2、液体样本计算公式：

$$\text{D-乳酸含量}(\mu mol/mL)=\frac{\Delta A+0.0101}{39.127\times V_1}\times N$$

3、组织样本按样本质量计算公式：

$$\text{D-乳酸含量}(\mu mol/g\text{组织})=\frac{\Delta A+0.0101}{39.127\times V_1}\div\frac{W}{V}\times N$$

4、组织（或细胞、细菌）样本按蛋白浓度计算公式：

$$\text{D-乳酸含量}(\mu mol/mg\text{蛋白})=\frac{\Delta A+0.0101}{39.127\times V_1}\div Cpr$$

5、细菌/细胞按数量计算公式：

$$\text{D-乳酸含量}(\mu mol/10^4\text{个细胞})=\frac{\Delta A+0.0101}{39.127\times V_1}\div\frac{\text{细胞数}}{V}\times N$$

以上公式中，

V_1 为反应时加入的样本体积，0.06mL；

V 为样本提取时加入的提取液的总体积，mL；

N 为样本测试前稀释倍数，未稀释为 1；

W 为组织样本质量，g；

Cpr 为匀浆液蛋白浓度,mg/mL；

细胞数为细菌/细胞前处理时的数量，10^4 个。

六、注意事项：

1、样本置于冰箱-20℃冷冻，可保存 1 个月左右；-70℃冷冻可保存 2～3 个月。温度越低保存时间越长。解冻后的样本或组织匀浆必须当天测定。

2、严重溶血及黄疸会使得测定结果偏高。

3、若用酶标仪读数，则可每管取 200μL 加到 96 孔板中，450nm 波长读数即可，且标曲也需同时测定。

公司地址：南京市玄武区中央路 258-27 号新立基大厦　　邮政编码：210009　　E-Mail：njjcbio@vip.163.com
联系电话：025-83360321、83360969、83551389　　技术支持：025-83360272、19951670086
