# Supplementary material for: Uricase deficiency in rats results in a variety of metabolic disorders, addition to gouty nephropathy
Source: PLoS One. 2025 Aug 22;20(8):e0330344. doi: 10.1371/journal.pone.0330344 (PMC12373213; doi:10.1371/journal.pone.0330344)
Supplement: S3 — (ZIP) [file pone.0330344.s004.zip › Glu.pdf]

葡萄糖(GLU)测试盒说明书(精简版)

（货号:A154-1-1　葡萄糖氧化酶法　微板法　96T）

免责声明：测试前请仔细阅读说明书,预试后再进行批量实验,否则由此导致的后果用户自行承担!

一、试剂组成及配制(试剂盒有效期 6 个月)

试剂组成	规　格	组　份	浓　度	保存条件
工作液	25mL×1 瓶	磷酸盐缓冲液	100mmol/L	**2～8℃ 避光**
		DHBS	2.0mmol/L	
		4-氨基安替比林	1.0mmol/L	
		葡萄糖氧化酶	10kU/L	
		氯化镁	3.5mmol/L	
		过氧化物酶	8kU/L	
标准品	0.1mL×1 支	葡萄糖	浓度见标签	
**附送 96 孔平底酶标板一块**				**室温放置**

二、测定原理：

$$D-\text{葡萄糖}+H_2O+O_2 \xrightarrow{GOD} D-\text{葡萄糖酸}+H_2O_2$$

$$H_2O_2+4-\text{氨基安替比林}+DHBS \xrightarrow{POD} \text{红紫色色素}$$

在 505nm 处测试，其颜色与葡萄糖浓度成正比。

三、所需仪器及试剂：

可调 505nm 波长的酶标仪，37℃水浴锅或恒温箱，蒸馏水，生理盐水，蛋白测定试剂（组织或细胞样本用，本公司有售）。

四、操作过程：

1、样本处理：

①、血清(或肝素抗凝血浆)：直接测定，如超过线性范围用生理盐水稀释后测定。

②、培养液样本：吸取培养液，1000 转/分，离心 10 分钟，取上清测定。**[注]：一般建议细胞密度在 100 万个/mL 以上。**

③、组织样本：准确称取组织重量，按重量(g)：体积(mL)=1：9 的比例，加入 9 倍体积的**生理盐水（或 PBS）**，冰水浴条件下机械匀浆，2500 转/分，离心 10 分钟，取上清液待测。

④、细胞样本：(建议收集的细胞密度在 100 万个/mL 以上。破碎好的液体可显微镜观察细胞是否破碎完全)

A、细胞收集：将制备好的细胞悬液取出，1000 转/分，离心 10 分钟，弃上清液，留细胞沉淀；用等渗缓冲液（推荐 0.1mol/L、pH7～7.4 磷酸盐缓冲液）清洗 1～2 次，同样 1000 转/分，离心 10 分钟，弃上清液，留细胞沉淀；

B、细胞破碎：加入 0.2～0.3mL 的匀浆介质（**推荐 0.1mol/L、pH7～7.4 磷酸盐缓冲液或生理盐水**）进行匀浆，冰水浴条件下超声破碎(功率 300W，3～5 秒/次，间隔 30 秒,重复 3～5 次)或手动匀浆，制备好的匀浆液不离心直接测定。也可采用裂解液裂解(推荐 TritonX-100，1～2%，裂解 30～40 分钟)，裂解好的液体不离心直接测定。

2、操作表：

**96 孔板操作，酶标仪比色**			
	空白孔	标准孔	样本孔
蒸馏水（μL）	2.5		
标准品（μL）		2.5	
样本（μL）			2.5
工作液（μL）	250	250	250
轻轻振荡孔板，37℃孵育 10 分钟，波长 505nm，酶标仪测定各孔吸光度值 A			

五、计算公式:

1、血清等液体样本计算公式：

酶标仪比色:

$$\text{葡萄糖含量}(mmol/L)=\frac{A_{\text{测定}}-A_{\text{空白}}}{A_{\text{标准}}-A_{\text{空白}}}\times C_{\text{标准}}\times\text{样本稀释倍数}$$

C 标准:标准品浓度,5.55mmol/L(具体浓度以标签为准)

2、组织、细胞计算公式（组织、细胞不建议使用生化分析仪测）：

$$\text{葡萄糖含量}(mmol/g\text{蛋白})=\frac{A_{\text{测定}}-A_{\text{空白}}}{A_{\text{标准}}-A_{\text{空白}}}\times C_{\text{标准}}\div Cpr$$

C 标准:标准品浓度,5.55mmol/L(具体浓度以标签为准)

注：蛋白浓度(Cpr,单位 g/L)测定试剂盒本所有售。（A045-2 考马斯亮蓝法、A045-3/-4 BCA 法）

六、产品描述:

本试剂盒可用于不同样本的葡萄糖含量测定，液体样本（参照血清操作和计算方法），固体样本（参照组织、细胞的操作及计算方法），主要用于酶标仪测定葡萄糖含量，人血清样本参考范围为（3.89-6.4mmol/L）。

七、性能指标:

1、试剂空白孔吸光度≤0.200（光径 1cm）。

2、线性：2.2～15mmol/L 范围内，$r^2>0.995$。

3、精密度：CV≤3%，批间相对极差≤5%。

4、稳定性：原装试剂盒在 2℃～8℃避光保存，有效期为 12 个月。开启后 2℃～8℃避光保存，可稳定 1 个月；试剂不可冷冻。

八、注意事项:

1、本产品仅用于科研，不得用于临床诊断，切勿服用。

2、样品含量如超出检测范围上限(15mmol/L)时，可用生理盐水稀释样本后进行测定，测定结果乘以稀释倍数;样本葡萄糖含量较低(小于 2.2mmol/L)时可以增加样本取样量(工作液量不变,且最大增加到 50μL，此时空白孔蒸馏水也要增加到相应体积，标准孔标准品量不变，多余的体积加蒸馏水补足，计算时标准品浓度除以相应稀释倍数（$\frac{\text{蒸馏水体积（μL）}+2.5}{2.5}$）代入计算)后测定。

九、参考文献:

1、《现代临床生化检验学》，张秀明、李建斋，2001，P_{84}

2、《实用医学检验学》，朱忠勇，1992，P_{423}

公司地址：南京市玄武区中央路 258-27 号新立基大厦　邮政编码：210009　E-Mail：njjcbio@vip.163.com
联系电话：025-83360321、83360969、83551389　技术支持：025-83360272、19951670086
