# Supplementary material for: Uricase deficiency in rats results in a variety of metabolic disorders, addition to gouty nephropathy
Source: PLoS One. 2025 Aug 22;20(8):e0330344. doi: 10.1371/journal.pone.0330344 (PMC12373213; doi:10.1371/journal.pone.0330344)
Supplement: S3 — (ZIP) [file pone.0330344.s004.zip › LDL-C and HDL-C测试盒说明书.pdf]

低密度脂蛋白胆固醇(LDL-C)测试盒说明书

（货号:A113-2-1 分光光度计法）

免责声明：测试前请仔细阅读说明书,预试后再进行批量实验,否则由此导致的后果用户自行承担!

一、试剂组成及配制:

试剂组成	规 格	保存条件
试剂一	75mL×1 瓶	2～8℃<br>避光保存
试剂二	25mL×1 瓶	
校准品	1 支(浓度及配置方法见标签)	

二、测定步骤:

1、样本处理:

①、血清(浆)：直接测定，如超过线性范围用生理盐水稀释后测定。

②、**培养液样本**：吸取培养液，1000 转/分钟，离心 10 分钟，取上清测定。

[注]：一般建议细胞密度在 100 万个/mL 以上。

③、**组织样本**：准确称取组织重量,按重量(g)：体积(mL)=1：9 的比例，加入 9 倍体积的匀浆介质，冰水浴条件下机械匀浆，2500 转/分，离心 10 分钟，取上清液待测。

[注]:1、如组织样本为非高脂样本，匀浆介质统一用磷酸盐缓冲液(0.1mol/L pH 7.4)或生理盐水进行提取。

2、如组织样本为高脂样本或部分为高脂样本，匀浆介质可统一用无水乙醇进行提取。

④、细胞样本:

A、细胞收集:将制备好的细胞悬液取出，1000 转/分，离心 10 分钟，弃上清液，留细胞沉淀；用等渗缓冲液（推荐 0.1mol/L 、pH7～7.4 磷酸盐缓冲液）清洗 1～2 次，同样 1000 转/分，离心 10 分钟，弃上清液，留细胞沉淀；

B、细胞破碎:加入 0.2～0.3mL 的匀浆介质（**推荐 0.1mol/L、pH7～7.4 磷酸盐缓冲液或生理盐水**）进行匀浆,冰水浴条件下超声破碎(功率:300W，3～5 秒/次，间隔 30 秒，重复 3～5 次)或手动匀浆，制备好的匀浆液不离心直接测定。也可采用裂解液裂解(推荐 TritonX-100，1～2%，裂解 30～40 分钟)，裂解好的液体不离心直接测定。

[注]：一般建议细胞密度在 100 万个/mL 以上。破碎好的液体可显微镜观察细胞是否破碎完全

2、操作表:

普通试管操作，分光光度计比色			
	空白管	校准管	样本管
蒸馏水（μL）	10		
校准品（μL）		10	
样本（μL）			10
试剂一（μL）	750	750	750
混匀，37℃孵育 5 分钟，波长 550nm，测定各管吸光度值 A1			
试剂二（μL）	250	250	250
混匀，37℃孵育 10 分钟，波长 550nm,测定各管吸光度值 A2，计算△A=A2-A1。			

三、计算公式及举例:

1、血清等液体样本计算公式:

酶标仪操作:

$$\text{LDLC含量}(\text{mmol/L})=\frac{\Delta A_{样本}-\Delta A_{空白}}{\Delta A_{标准}-\Delta A_{空白}}\times C_{校准}$$

$C_{标准}$:标准品浓度,mmol/L。

a、取正常人血浆10μL，按操作表操作，得空白管A_1为0.005，空白管A_2为0.012，校准管A_1为0.039，校准管A_2为0.271，样本管A_1为0.018，样本管A_2为0.092，则计算如下：

$$\text{LDLC含量}(\text{mmol/L})=\frac{0.074-0.007}{0.232-0.007}\times 4.3=1.2804\ \text{mmol/L}$$

b、取大鼠血清10μL，按操作表操作，得空白管A_1为0.005，空白管A_2为0.012，校准管A_1为0.039，校准管A_2为0.271，样本管A_1为0.010，样本管A_2为0.040，则计算如下：

$$\text{LDLC含量}(\text{mmol/L})=\frac{0.030-0.007}{0.232-0.007}\times 4.3=0.4396\ \text{mmol/L}$$

2、组织、细胞样本计算公式:（组织样本不建议使用生化仪测定）

①、用 PBS 或生理盐水作匀浆介质提取样本计算方法（此方法需要另外测定匀浆液蛋白浓度）:

酶标仪比色:

$$\text{LDLC含量}(\text{mmol/gprot})=\frac{\Delta A_{样本}-\Delta A_{空白}}{\Delta A_{标准}-\Delta A_{空白}}\times C_{校准品}\div Cpr$$

Cpr:匀浆液蛋白浓度,gprot/L(prot 指蛋白)。

②、用无水乙醇作匀浆介质提取样本计算方法（此方法不需要另外测定匀浆液蛋白浓度）:

酶标仪操作:

$$\text{LDLC含量}(\text{mmol/g组织})=\frac{\Delta A_{样本}-\Delta A_{空白}}{\Delta A_{标准}-\Delta A_{空白}}\times C_{校准品}\div\frac{W}{V_{样总}}$$

W: 样本质量（g）；**$V_{样总}$:** 匀浆液总体积（L）。

注：细胞样本测定时可将上式中的 $\frac{W}{V_{样总}}$ 替换为细胞前处理时的细胞密度。

a、取10%小鼠肝匀浆10μL，按操作表操作，得空白管A_1为0.005，空白管A_2为0.012，校准管A_1为0.039，校准管A_2为0.271，样本管A_1为0.042，样本管A_2为0.066，同时测得10%小鼠肝匀浆蛋白浓度为12.0121gprot/L，计算如下：

$$\text{LDLC含量}(\text{mmol/gprot})=\frac{0.024-0.007}{0.232-0.007}\times 4.3\div 12.0121=0.027\ \text{mmol/gprot}$$

四、性能指标:

1、试剂空白管△A≤0.050（光径 1cm）。

2、**灵敏度**：测试 3.3mmol/L 被测物时，吸光度差值△A 为 0.19～0.290

3、**线性范围**：0.2～18.0mmol/L，r^2＞0.995

4、**精密度**：变异系数≤8%，批间差：相对偏差≤10%

5、**稳定性**：原包装试剂盒在 2℃～8℃避光保存，有效期为 12 个月。开启后 2℃～8℃避光保存，可稳定一个月。

五、测定原理:

$$\text{LDL-C}+H_2O\xrightarrow{\text{CHER 化学修饰}}\text{胆固醇}+\text{游离脂肪酸}$$

$$\text{胆固醇}+O_2\xrightarrow{\text{CHOD 化学修饰}}\text{胆甾烯酮}+H_2O_2$$

公司地址：南京市玄武区中央路 258-27 号新立基大厦 邮政编码：210009 E-Mail：njjcbio@vip.163.com
联系电话：025-83360321、83360969、83551389 技术支持：025-83360272 传真号码：025-83227943

$$2H_2O_2 + 4-AA + HSDA + H_3^+O \xrightarrow{\text{过氧化物酶}} \text{苯醌色素} + 5H_2O$$

六、注意事项：

1、本产品仅用于科研，不得用于临床诊断，切勿服用。

2、样品含量如超出检测范围上限时，可用生理盐水稀释样本后进行测定，测定结果乘以稀释倍数；如样品含量较低也可以适当加大进样量（如 5 或 10μL），同时要将标准品稀释相应的倍数后和样本保持相同的加样量,试剂一、二量不变。

3、试剂防止葡萄糖、胆固醇等试剂的污染。

4、试剂与样本量可按照全自动生化分析仪的要求，按比例增减。

5、举例采用标准品为不同批次，浓度不同。

七、参考文献：

1、H. Wieland and D. Seidel, J.Lipid Res. 24, 904(1983).
2、G. Assmann, Internist 20,559(1979).

八、参考值：

大鼠血浆：1.76±0.46mmol/L
小鼠血浆：1.08±0.84 mmol/L
小鼠肝（生理盐水）匀浆：24.56±5.4μmol/gprot
绵羊血清：0.91±0.36 mmol/L
鱼血浆：1.26±0.42 mmol/L
鱼肝（生理盐水）匀浆：22.75±6.18μmol/gprot
虾血清：0.11±0.03 mmol/L
蟹血清：0.31±0.13 mmol/L

注：以上值仅供参考，并无临床学意义。

高密度脂蛋白胆固醇(HDL-C)测试盒说明书

（货号:A112-2-1　分光光度计法）

免责声明：测试前请仔细阅读说明书,预试后再进行批量实验,否则由此导致的后果用户自行承担!

一、试剂组成及配制：

试剂组成	规 格	保存条件
试剂一	75mL×1 瓶	2～8℃ 避光保存
试剂二	25mL×1 瓶	
校准品	1 支(浓度见标签)	

二、测定原理：

HDL- C+ H_2O ——CHER 化学修饰——→ 胆固醇 ＋ 游离脂肪酸

胆固醇 ＋ O_2 ——CHOD 化学修饰——→ 胆甾烯酮 ＋ H_2O_2

$2H_2O_2$ ＋ 4 － AA ＋ HSDA ＋ H_3^+O ——过氧化物酶——→ 苯醌色素 ＋ $5H_2O$

三、操作过程：

1、样本处理：

①、血清(浆)：直接测定，如超过线性范围用生理盐水稀释后测定。

②、培养液样本：吸取培养液，1000 转/分，离心 10 分钟，取上清测定。**[注]：一般建议细胞密度在 100 万个/mL 以上。**

③、组织样本：准确称取组织重量,按重量(g)：体积(mL)=1：9 的比例，加入 9 倍体积的匀浆介质，冰水浴条件下机械匀浆，2500 转/分，离心 10 分钟，取上清液待测。**[注]:如组织样本为非高脂样本，匀浆介质统一用磷酸盐缓冲液(0.1mol/L pH 7.4)或生理盐水进行提取;如组织样本为高脂样本或部分为高脂样本，匀浆介质可统一用无水乙醇进行提取。**

④、细胞样本：

A、细胞收集:将制备好的细胞悬液取出，1000 转/分，离心 10 分钟，弃上清液，留细胞沉淀；用等渗缓冲液（推荐 0.1mol/L 、pH7～7.4 磷酸盐缓冲液）清洗 1～2 次，同样 1000 转/分，离心 10 分钟，弃上清液，留细胞沉淀；

B、细胞破碎:加入 0.2～0.3mL 的匀浆介质（**推荐 0.1mol/L、pH7～7.4 磷酸盐缓冲液或生理盐水**）进行匀浆，冰水浴条件下超声破碎(功率：300W,3～5 秒/次，间隔 30 秒，重复 3～5 次)或手动匀浆，制备好的匀浆液不离心待测。也可采用裂解液裂解(推荐 TritonX-100，1～2%，裂解 30～40 分钟)，裂解好的液体不离心直接测定。**[注]：建议细胞密度在 100 万个/mL 以上。破碎好的液体可显微镜观察细胞是否破碎完全**

2、操作表：

普通试管操作，分光光度计比色			
	空白管	校准管	样本管
蒸馏水（μL）	10		
校准品（μL）		10	
样本（μL）			10
试剂一（μL）	750	750	750
混匀，37℃孵育 5 分钟，波长 550nm，测定各管吸光度值 A1			
试剂二（μL）	250	250	250
混匀，37℃孵育 10 分钟，波长 550nm，测定各管吸光度值 A2，计算△A=A2-A1。			

全自动生化分析仪上机操作			
样本/校准品/水	Sample Volume	μL	2.5
R1	Reagent	μL	180
37℃孵育 5 分钟，波长 600nm，测定光吸收值 A1			
R2	Reagent	μL	60
37℃孵育 5-10 分钟，波长 600nm，测定光吸收值 A2			
主波长	Main wavelength	nm	550
反应类型	Reaction type		终点法
反应方向	Reaction direction		升反应(+)

四、计算公式及举例：

1、血清等液体样本计算公式：

$$\text{HDLC含量}(\text{mmol/L}) = \frac{\Delta A_{样本} - \Delta A_{空白}}{\Delta A_{标准} - \Delta A_{空白}} \times C_{校准}$$

$C_{标准}$:标准品浓度,mmol/L。

例 1：取正常人血浆 10μL，按操作表操作，得空白管 A_1 为 0.000，空白管 A_2 为 0.010，$\triangle A_{空白}$为 0.010；校准管 A_1 为 0.048，校准管 A_2 为 0.139，则$\triangle A_{校准}$为 0.091；样本管 A_1 为 0.042，样本管 A_2 为 0.088，则$\triangle A_{样本}$为 0.046；则计算如下：

$$\text{HDLC含量}(\text{mmol/L}) = \frac{0.046 - 0.010}{0.091 - 0.010} \times 1.8 = 0.80 \quad \text{mmol/L}$$

例2：取大鼠血清10μL，按操作表操作，得空白管A_1为0.000，空白管A_2为0.010，$\triangle A_{空白}$为0.010；校准管A_1为0.048，校准管A_2为0.139，则$\triangle A_{校准}$为0.091；样本管A_1为0.010，样本管A_2为0.048，则$\triangle A_{样本}$为0.038；则计算如下：

$$\text{HDLC含量}(\text{mmol/L}) = \frac{0.048 - 0.010}{0.091 - 0.010} \times 1.8 = 0.2667 \quad \text{mmol/L}$$

2、组织、细胞样本计算公式：

①、用 PBS 或生理盐水作匀浆介质提取样本计算方法（**此方法需要另外测定匀浆液蛋白浓度，蛋白测定试剂盒本所有售，货号为 A045-2 或者 A045-4**）：

$$\text{HDLC含量}(\text{mmol/gprot}) = \frac{\Delta A_{样本} - \Delta A_{空白}}{\Delta A_{标准} - \Delta A_{空白}} \times C_{标准} \div \text{Cpr}$$

注:Cpr 为匀浆液蛋白浓度，gprot/L（prot 指蛋白）

②、用无水乙醇作匀浆介质提取样本计算方法（**此方法不需要另外测定匀浆液蛋白浓度**）：

$$\text{HDLC含量}(\text{mmol/g组织}) = \frac{\Delta A_{样本} - \Delta A_{空白}}{\Delta A_{标准} - \Delta A_{空白}} \times C_{标准} \div \frac{W}{V_{乙醇}}$$

W：组织样本质量，g；

$V_{乙醇}$：样本提取时加入的乙醇的总体积，L 。

注：细胞样本测定时可将上式中的 $\frac{W}{V_{乙醇}}$ 替换为细胞前处理时的细胞密度（10^4 个/L）。

例1：取10%小鼠肝匀浆10μL，按操作表操作，得空白管A_1为0.000，空白管A_2为0.010，$\triangle A_{空白}$为0.010；校准管A_1为0.048，校准管A_2为0.139，则$\triangle A_{校准}$为0.091；样本管

公司地址：南京市玄武区中央路 258-27 号新立基大厦　　邮政编码：210009　　E-Mail：njjcbio@vip.163.com
联系电话：025-83360321、83360969、83551389　　技术支持：025-83360272　　传真号码：025-83227943

A_1为0.040，样本管A_2为0.064，则$\triangle A_{样本}$为0.024；同时测得10%小鼠肝匀浆蛋白浓度为18.1133gprot/L，计算如下：

$$\text{HDLC含量(mmol/gprot)} = \frac{0.024-0.010}{0.091-0.010} \times 1.64 \div 18.1133$$

$$= 0.015.6 \text{ mmol/gprot} = 15.6\mu\text{mol/gprot}$$

五、性能指标：

1、试剂空白管吸光度△A≤0.02。

2、线性范围：0.09～2.50mmol/L，r^2＞0.990。

3、灵敏度：测试 1.00mmol/L 被测物时，吸光度值△A 大于 0.04。

4、准确度：相对偏差≤10%。

5、精密度：CV≤3%，批间相对极差≤5%。

6、稳定性：原包装试剂盒在 2℃～8℃避光保存，有效期为 12 个月。开启后 2℃～8℃避光保存，可稳定一个月。

六、注意事项：

1、本产品仅用于科研，不得用于临床诊断，切勿服用。

2、样品含量如超出检测范围上限时，可用生理盐水稀释样本后进行测定，测定结果乘以稀释倍数。

3、试剂防止葡萄糖、胆固醇等试剂的污染。

4、试剂与样本量可按照仪器要求，按比例增减。

5、样本中 HDL-C 含量较低时，可以加大样本取样量（如取 50μL 或 100μL，同时标准品需要稀释相应的倍数后和样本取样量一致,试剂一、二量不变）后测定。

6、标准品粉剂为冻干粉，溶解时间较长，配置时可提前半小时配制。

七、参考文献：

1、National Institutes of Health Consensus Development Confrernce Statement:Triglyceride ,High Density Lipoprotein and Coronary Heart Disence. Washington D.C. Feb 26-28, 1992.

2、Sugiuchi, H. ,Uji,Y., Okabe, H. , Irie T. , Uekama, K. ,Kayahara, N; Clin Chem 1995; 1/5:717-723.

八、参考值：

大鼠血浆：0.45±0.16mmol/L

小鼠血浆：0.92±0.11 mmol/L

鸡血浆：0.36±0.12（某些种类的能达到 2.33±0.38）mmol/L

鱼血浆：0.99±0.22 mmol/L

小鼠肝脏：12.4±3.9 μmol/gprot

鱼肝脏：39.36±12.37μmol/gprot

注：以上值仅供参考，并无临床学意义。

公司地址：南京市玄武区中央路 258-27 号新立基大厦　邮政编码：210009　E-Mail：njjcbio@vip.163.com
联系电话：025-83360321、83360969、83551389　技术支持：025-83360272　传真号码：025-83227943
