# Supplementary material for: Uricase deficiency in rats results in a variety of metabolic disorders, addition to gouty nephropathy
Source: PLoS One. 2025 Aug 22;20(8):e0330344. doi: 10.1371/journal.pone.0330344 (PMC12373213; doi:10.1371/journal.pone.0330344)
Supplement: S3 — (ZIP) [file pone.0330344.s004.zip › RX302991R(CRP ELISA).pdf]

(仅供科研使用，不得用于临床诊断!)

大鼠C-反应蛋白（CRP）试剂盒（ELISA）
使用说明书

货号： RX302991R

规格： 48T/96T

检测范围： 75→2400 ng/mL

特异性： 结构类似物无交叉

灵敏度： <10 ng/mL

重复性： 板内变异系数均<10%，板间变异系数均<15%。

保存、有效期： 2-8℃保存6个月

用途： 定量检测血清、血浆、细胞培养上清液等样本中大鼠C-反应蛋白（CRP）的浓度。

使用试剂盒前，必须仔细阅读本说明书。如有任何问题，请通过以下方式联系我们：

免费电话：400-8332-227

官方热线：0595-2284-5743

技术电话：15260335612

公司网址：www.ruixinbio.com

联系时请提供产品货号、生产日期（见盒签），以便我们更高效为您服务。

实验原理

本试剂盒采用双抗体夹心酶联免疫吸附试验（ELISA）。在预包被抗大鼠 C-反应蛋白（CRP）抗体（固相抗体）的微孔酶标板中，加入大鼠 C-反应蛋白（CRP）校准品和待测样本，再加入另一株 HRP 标记的抗人大鼠 C-反应蛋白(CRP）抗体（酶标抗体），经过温育与充分洗涤，去除未结合的组分，在微孔板固相表面形成固相抗体-抗原-酶标抗体的夹心复合物。加底物 A 和 B，底物在 HRP 催化下，产生蓝色产物，在终止液（2M 硫酸）作用下，最终转化为黄色，在酶标仪 450nm 波长上测定吸光度（OD 值），吸光度（OD 值）与待测样品中大鼠 C-反应蛋白（CRP）的浓度正相关。拟合校准品曲线，可以计算出样本中大鼠 C-反应蛋白（CRP）的浓度。

试剂盒限制性

1、供科研使用，不得用于临床诊断。

2、在试剂盒标示的有效期内使用，过期产品不得使用。

3、跟其他厂家的试剂盒或者组分不能混用。

4、使用试剂盒配套的样品稀释液。

5、如果样本值高于最高标准品浓度值，请将样本适当稀释后，再重新测定。

6、待测样本中存在的人抗鼠等异嗜抗体会干扰检测结果，检测前，请排除该因素。

7、通过其他方法得到的检测结果，与本试剂盒测定结果不具有直接的可比性。

技术提示

1、混合蛋白溶液时，避免起泡。

2、加校准品与样本时，每个校准品浓度和样本都要更换移液枪头，公共组分应该悬臂加样，避免交叉污染。

3、合适的温育时间，和充分的洗涤步骤，是保证实验结果准确性的必要条件。

4、使用自动洗板机时，加入一个 30 秒浸泡的步骤，可以提高检测精度。

5、底物溶液为无色液体，保存过程中变为蓝色，代表底物溶液已经失效，不得使用。

6、终止液加样顺序与底物溶液加样顺序一致，加入终止液后，蓝色底物产物，会瞬间变为黄色。

7、实验中，用剩的板条，应立即放回自封袋中，密封（低温干燥）保存。

8、所有液体组分，使用前充分摇匀，严格按照说明书标明的时间、加样量及加样顺序进行温育操作。

未提供仪器用品

1、酶标仪（450nm）

2、精密移液器及一次性吸头

3、蒸馏水

4、洗瓶或者自动洗板机

5、37℃水浴锅或恒温箱

6、500ml 量筒

试剂盒组分与保存

未开封的试剂盒保存在 2-8 度，不得使用过期试剂盒。

组分	数量	主要成分	开封后储存
校准品	0.3ml/管	--	2-8℃14 天
包被微孔板	96T/48T	预包被固相抗体	2-8℃14 天
HRP 标记抗体	10mL	HRP 标记的检测抗体	2-8℃180 天
样本稀释液	6mL	--	2-8℃180 天
底物液 A	6mL	0.01%过氧化氢	2-8℃180 天
底物液 B	6mL	0.1%TMB	2-8℃180 天
终止液	6mL	2mol/L 稀硫酸	2-8℃180 天
20×浓缩洗涤液	25mL	0.05%Tween20	2-8℃180 天
说明书	1 份	--	--
自封袋	1 个	--	--
不干胶	2 片	--	--

校准品浓度依次为：2400、 1200、 600、300、 150、 75 ng/mL。

注意：

1：如果试剂盒的组份需要再次使用，请确保上一次使用之后没有被污染。

2：酶标板单次未使用完，要谨记密封放到 2-8℃保存。

生物安全

1、检测必须符合实验室管理规范的规定，严格防止交叉污染，所有样品、洗弃液和各种废弃物都应按照传染物进行处置。

2、试剂盒的液体组分中，含有 proclin-300 防腐剂，可能引起皮肤过敏反应，避免吸入烟雾。

3、避免与皮肤接触。

4、底物液对皮肤、眼睛和上呼吸道有刺激作用，避免吸入烟雾。

5、戴上防护手套，实验完成后彻底洗手。

样品的采集和储存

以下只是列出样品采集和保存的一般指南。所有样本采集保存过程中，不得使用叠氮钠做为防腐剂。

1、细胞培养上清：4000rpm 条件下离心 20min，去除细胞颗粒和聚合物，上清液保存在- 20℃以下，避免反复冻融。

2、血清：使用不含热原和内毒素的试管，操作过程中避免任何细胞刺激，4000rpm 条件下离心 20min，小心地分离出血清，保存在-20℃以下，避免反复冻融。

3、血浆：肝素，EDTA，或柠檬酸钠作为抗凝剂。在 4000rpm 条件下，离心 20 分钟取上清，血浆保存在-20℃以下，避免反复冻融。

样本收集后，无法一次检测完毕，请按一次用量分装冻存，避免反复冻融，使用时在室温下解冻，确保样品均匀充分解冻。

4、组织匀浆：用预冷的 PBS(0.01M,pH=7.4)冲洗组织，去除残留血液，称重后将组织剪碎。将剪碎的组织与对应体积的 PBS(一般按 1：9 的重量体积比，比如 1g 的组织样品对应 9 mL 的 PBS，具体体积可根据实验需要适当调整，并做好记

录。推荐在 PBS 中加入蛋白酶抑制剂)加入玻璃匀浆器中，在冰上充分研磨。为了进一步裂解组织细胞，可以对匀浆液进行超声破碎或反复冻融。最后将匀浆液 5000×g 离心 5-10 分钟，取上清检测。

5、细胞提取液:贴壁细胞用冷的 PBS 轻轻清洗，然后用胰蛋白酶消化，1000×g 离心 5 分钟后收集细胞；悬浮细 胞可直接离心收集。收集的细胞用冷的 PBS 洗涤 3 次。每 1×106 个细胞中加入 150-200μLPBS 重悬并通过反复冻融使细胞破碎(若含量很低可减少 PBS 的体积)。将提取液于 1500×g 离心 10 分钟，取上清检测。

6、其他生物体液：1000×g 离心 20 分钟，除去杂质及细胞碎片。取上清检测。

试剂准备

1、使用前，样本和试剂盒所有的组分都要至少复温 120min，确保充分复温到室温。

2、浓缩洗涤液：从冰箱取出的浓缩洗涤液，会有结晶产生，这属于正常现象，水浴加热使结晶完全溶解。浓缩洗涤液与蒸馏水，按 1:20 稀释，即 1 份的浓缩洗涤液，添加 19 份的蒸馏水。

3、底物:底物液 A 和 B，在使用前，按 1:1 体积充分混合，混合后 15 分钟内使用。

操作程序

所有试剂和组分都先恢复到室温，标准品、质控品和样品，建议做复孔。

1、按前面说明书描述的方法，配制好试剂盒各种组分的工作液。

2、从铝箔袋中取出所需板条，剩余的板条用自封袋密封放回冰箱。

3、设置标准品孔、0 值孔、空白孔和样本孔，标准品孔各加不同浓度的标准

品 50μL，0 值孔加样本稀释液 50μL，空白孔不加，样本孔加待测样本 50μL。

4、除空白孔外，标准品孔、0 值孔和样本孔，加入辣根过氧化物酶（HRP）标记的检测抗体 100μL。

5、用封板膜盖住反应板，37℃水浴锅或恒温箱避光孵育 60min。

6、揭开封板膜，弃去液体，吸水纸上拍干，每孔加满洗涤液，静置 20S，甩去洗涤液，吸水纸上拍干，如此重复 5 次。若使用自动洗板机，请按洗板机操作程序进行洗板，添加浸泡 30s 的程序，可以提高检测的精度。洗板结束，加底物前，要在干净不掉屑的纸上，充分拍干反应板。

7、将底物 A 和 B 按 1:1 体积充分混合，所有孔中加入底物混合液 100μL。用封板膜盖住反应板，37℃水浴锅或恒温箱避光孵育 15min。

8、所有孔加入终止液 50μL，在酶标仪上读取各孔吸光度（OD 值）。

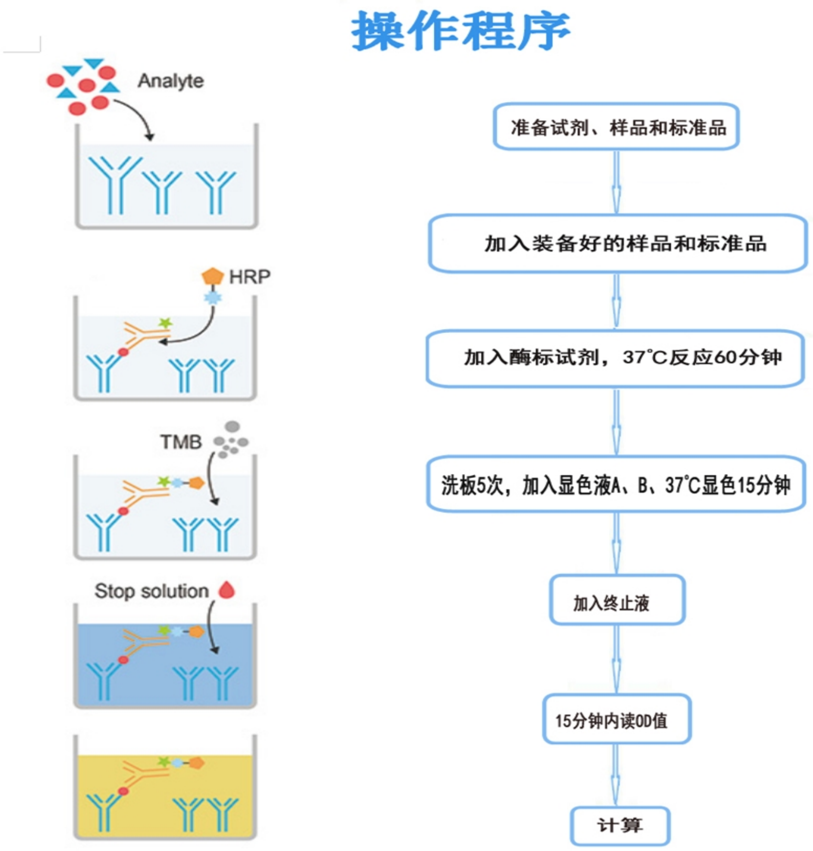

结果计算

1、以标准品浓度做为横坐标（6 个标准品孔，加 1 个 0 值孔，共 7 个浓度点），对应的吸光度（OD 值）作为纵坐标，利用计算机软件，采用四参数 Logistic 曲线拟合（4-pl），创建标准曲线方程，通过样本的吸光度（OD 值），利用方程计算样品的浓度值。

2、如果样品被稀释，通过上述方法测的浓度值，要乘以稀释倍数，才是样品的最终浓度。

典型数据

以下数据和曲线仅供参考，实验者需根据自己的实验建立标准曲线。

浓 度 ：(ng/mL)	2400	1200	600	300	150	75
OD 值	1.848	0.972	0.557	0.308	0.136	0.061

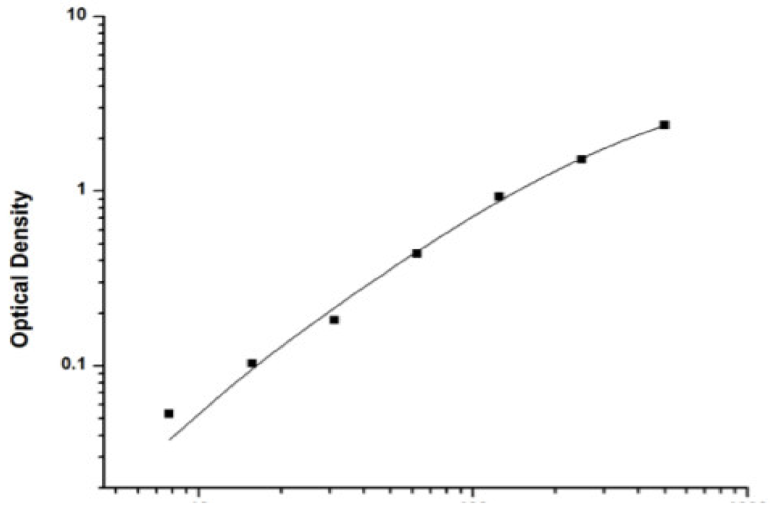

（示意图，仅供参考）

试剂盒性能指标

1、物理性能

试剂盒的各液体组分应澄清透明、无沉淀或者絮状物。微孔板铝箔袋应真空包装，无破损漏气。

2、剂量反应曲线线性

校准品剂量反应曲线相关系数 r 值，大于等于 0.9900。

3、精密度

批内精密度：三组已知的高、中、低浓度样品，进行二十次在同一个板块内精度评估。

批间精密度：三组已知的高、中、低浓度样品，进行二十次在不同板块内精度评估。

	批内变异系数			批间变异系数		
样本	1	2	3	1	2	3
数量	20	20	20	20	20	20
平均值(ng/mL)	255.9	629.3	1444.5	228.6	652	1476.6
标准差	10.49	36.5	59.22	21.95	50.2	116.65
变异系数 (%)	4.1	5.8	4.1	9.6	7.7	7.9

4、灵敏度

最低检出剂量小于 0.1 mIU/L(6 次独立实验的平均值)。

10 个零标准品浓度 OD 的平均值加上两倍 SD，计算最低可检测浓度。

5、回收率

分别往不同样本中添加已知的高、中、低浓度目的蛋白样品，进行五次在同一个板块内回收率评估，回收率在 85%-115%之间。

样本类型	回收率范围（%）	平均回收率（%）
血清(n=5)	89-107	102
血浆(EDTA)(n=5)	80-90	86
细胞培养基(n=5)	94-115	111

6、特异性

本试剂盒识别天然和重组大鼠 C-反应蛋白（CRP），与结构类似物无交叉。

7、稀释线性

将添加有高浓度大鼠 C-反应蛋白（CRP）的样本分别稀释2倍，4倍，8倍，16 倍做回收实验，得出回收率范围及平均回收率。

		血清	血浆(EDTA)(n=5)	细胞培养基(n=5)
1:2	回收率范围(%)	87-98	83-93	96-105
1:4	回收率范围(%)	88-97	92-103	86-103
1:8	回收率范围(%)	91-105	90-102	94-106
1:16	回收率范围(%)	91-179	84-172	81-177

[说明]

1、由于现有条件及科学技术水平尚不能对供货商提供的所有所有原料进行全面的鉴定与分析，本产品可能存在一定的质量技术风险。

2、最终的实验结果与试剂的有效性、实验者的相关操作以及实验环境密切相关，请务必准备充足的标本备份。

3、不同批次的同一产品可能会有少许差别，网站电子版说明书仅作参考。

4、本试剂盒配套试剂必须配套使用，不能混用其他制造商的产品。只有严格遵守本试剂盒的实验说明才会得到最佳的检测结果。

5、用于制备试剂盒中抗体的免疫原通常为重组蛋白，但由于备重组蛋白所选取的片段、表达系统、纯化方式等各有不同，所以我们无法保证该试剂盒可用于其他公司重组蛋白的检测，通常我们也不建议使用试剂盒检测重组蛋白。

6、该试剂盒仅供研究使用，如将其用于临床诊断或任何其他用途，我公司将不对因此产生的问题负责，亦不承担任何法律责任。

[**警告**]

本试剂盒中使用了稀硫酸作为终止液，其具有轻微腐蚀性，使用时应避免接触衣物或眼、手等皮肤暴露部位。

[**问题解答**]

问题	可能原因	解决方案
**标准曲线差**	试剂盒平衡时间不够	不低于 2 个小时的室温平衡
	吸取及洗涤不充分	充分的吸取及洗涤
	移液不精确	检查和校正移液器
**精密度低**	洗涤不充分	按说明书要求充分洗涤和浸泡
	混匀不充分和吸取试剂不足	充分混匀和吸取试剂
	重复利用吸头、容器和覆膜	使用加样器要更换新的吸头、使用新的封板模
	加样不精确	检查和校正移液器
**O.D 值低**	每孔加的试剂量不精确	校正移液器，精确加入试剂
	温育时间不正确	保证充足的温育时间
	温育温度不正确	试剂要平衡至室温并保证准确的温育温度
	酶标记物或底物失效	通过混合酶标记物和底物，颜色迅速显现来检查
	没有加入终止液	按照说明书实验操作步骤加入终止液
	超出读数时间读数	在说明书推荐的读数时间内读数
**样本值**	不正确的样本储存方式	正确储存样本，使用新鲜样本进行实验
	不正确的样本收集和处理方法	采取正确的样本收集和处理方法
	待测物质在样本中含量低	使用新鲜样本，重复实验
