# Supplementary material for: Uricase deficiency in rats results in a variety of metabolic disorders, addition to gouty nephropathy
Source: PLoS One. 2025 Aug 22;20(8):e0330344. doi: 10.1371/journal.pone.0330344 (PMC12373213; doi:10.1371/journal.pone.0330344)
Supplement: S3 — (ZIP) [file pone.0330344.s004.zip › TAA.pdf]

总氨基酸测定试剂盒说明书(精简版)

(货号：A026-1-1　80 管/78 样　比色法)

免责声明：测试前请仔细阅读说明书,预试后再进行批量实验，
否则由此导致的后果用户自行承担!

一、试剂的组成与配制：（试剂盒有效期 6 个月）

试剂一：粉剂×1 支，4℃保存。

试剂二：液体 5mL×1 瓶，4℃保存。

氨基酸反应液的配制：将试剂一加双蒸水 160mL，充分混匀成蓝色混悬液，再缓慢滴加试剂二，边滴边搅至混悬液全部转换成淡蓝色透明溶液为止，4℃保存（粉剂较难溶解，试剂二滴加完后还需室温搅拌混匀半小时以上才能溶解完全）。

试剂三：粉剂×1 支，4℃保存。

氨基酸显色剂配制：将试剂三加水 80mL 充分混匀。（注意：有腐蚀性，配制时勿碰皮肤。）

试剂四：甘氨酸标准品 75.07mg/支×6 支，4℃保存。

50mmol/L 甘氨酸标准溶液的配制：将一支甘氨酸标准品溶于 20mL 双蒸水中，充分混匀，现用现配。

试剂五：液体 100mL×1 瓶，4℃保存。

二、所需仪器耗材及试剂：

含 650nm 波长的分光光度计及 1cm 光径比色皿（或酶标仪及 96 孔板）、台式低速离心机（3500rpm）、各种规格移液器、双蒸水、生理盐水（0.9%）或 PBS（0.1M）、涡旋混匀器、试管或离心管、蛋白测定试剂（组织及细胞样本用，本公司有售）。

三、操作步骤：

（一）、尿液的测定：

1、操作表：

	空白管	标准管	测定管
双蒸水（mL）	1		
50μmol/mL 氨基酸标准液（mL）		1	
尿液（mL）			1
氨基酸反应液（mL）	2	2	2
旋涡混匀			
氨基酸显色剂（mL）	1	1	1
混匀，3500 转/分离心 10 分钟，取上清液于 650nm 处，1 ㎝光径，双蒸水调零比色。			

2、计算公式：

$$\text{尿液中总氨基酸含量}(\mu mol/mL) = \frac{A_{测定} - A_{空白}}{A_{标准} - A_{空白}} \times C_{标准} \times N$$

C 标准:标准液浓度, 50μmol/mL;

N:样本测试前稀释倍数。

（二）、血清（浆）的测定：

1、操作表：

	空白管	标准管	测定管
双蒸水（mL）	0.3		
50μmol/mL 氨基酸标准液（mL）		0.3	
血清（浆）（mL）			0.3
试剂五（mL）	1.2	1.2	1.2
充分混匀，3500 转/分离心 10 分钟取 1mL **上清液**待测。			
**上清液**（mL）	1	1	1
氨基酸反应液（mL）	2	2	2
旋涡混匀			
氨基酸显色剂（mL）	1	1	1
混匀，3500 转/分离心 10 分钟，取上清液于 650nm 处，1 ㎝光径，双蒸水调零比色。			

2、计算公式：

$$\text{血清中总氨基酸含量}(\mu mol/mL) = \frac{A_{测定} - A_{空白}}{A_{标准} - A_{空白}} \times C_{标准} \times N$$

C 标准:标准液浓度, 50μmol/mL;

N：样本测试前稀释倍数。

（三）、组织的测定：

1、样本前处理：准确称取组织重量，按重量体积比加入 9 倍生理盐水，制成 10%匀浆，3500 转/分离心 10 分钟，取上清待测。同时取 10%匀浆上清进行蛋白测定。

2、操作表：

	空白管	标准管	测定管
双蒸水（mL）	0.3		
50μmol/mL 氨基酸标准液（mL）		0.3	
匀浆（mL）			0.3
试剂五（mL）	1.2	1.2	1.2
充分混匀，3500 转/分离心 10 分钟取 1mL 上清待测。			
上清（mL）	1	1	1
氨基酸反应液（mL）	2	2	2
旋涡混匀			
氨基酸显色剂（mL）	1	1	1
混匀，3500 转/分离心 10 分钟，取上清液于 650nm 处，1 ㎝光径，双蒸水调零比色。			

3、计算公式：

$$\text{组织中总氨基酸含量}(\mu mol/mg\text{蛋白}) = \frac{A_{测定} - A_{空白}}{A_{标准} - A_{空白}} \times C_{标准} \div Cpr$$

C 标准:标准液浓度, 50μmol/mL;

Cpr:组织样本蛋白浓度，mg/mL。

4、计算举例：

取 10%小鼠肝组织匀浆 0.3mL 按操作表进行检测，测得空白管吸光度为 0.051，标准管吸光度为 0.123，测定管吸光度为 0.067，同时测得 10%匀浆上清蛋白浓度为 8.251mgprot/mL,则计算结果为:

$$\text{组织中总氨基酸含量}(\mu mol/mg\text{蛋白}) = \frac{0.067 - 0.051}{0.123 - 0.051} \times 50 \div 8.251$$
$$= 1.347\ \mu mol/mg\text{蛋白}$$

四、测定原理：

铜离子（Cu^{2+}）能与各种氨基酸络合产生蓝绿色络合物，在一定波长下颜色的深浅与总氨基酸的含量成正比，故可以用可见光分光光度计测其吸光度，通过换算得到总氨基酸含量。

五、注意点：

1、在滴加试剂二时，一定要缓慢，且尽量要使混悬物完全溶解。使氨基酸反应液成为浅蓝色透明溶液。

2、试剂三在配制时要注意，尽量避免碰到皮肤，该试剂有一定腐蚀性。

3、做本试验后的试管要用热水加肥皂粉煮，自来水冲洗干净，否则用这种试管测量其它酶反应时会影响酶的活力。推荐使用一次性实验用塑料试管操作（本所有售）。

六、优点：

1、简单、便宜。目前国内外检测氨基酸的方法太复杂，且费用相当昂贵。

2、快速。本反应只要 15 分钟即可结束检查。

3、稳定性好，该法经多次重复试验，其结果相当稳定。

4、本法测定的是游离态的氨基酸。

公司地址：南京市玄武区中央路 258-27 号新立基大厦　邮政编码：210009　E-Mail：njjcbio@vip.163.com
联系电话：025-83360321、83360969、83551389　技术支持：025-83360272　传真号码：025-83227943

附录Ⅰ:标准曲线的制备

1、操作步骤：

①、**50mmol/L 甘氨酸标准溶液的配制：**将一支甘氨酸标准品溶于 20mL 双蒸水中，充分混匀，现用现配。

②、**操作表：**

	0	1	2	3	4	5
50mmol/L 氨基酸标准品(mL)	0	0.2	0.4	0.6	0.8	1.0
双蒸水(mL)	1.0	0.8	0.6	0.4	0.2	0
氨基酸反应液(mL)	2	2	2	2	2	2
旋涡混匀						
氨基酸显色液(mL)	1	1	1	1	1	1
混匀，3500 转/分离心 10 分钟，取上清于 650nm 处，1 ㎝光径，双蒸水调零比色。						

2、测定结果：

	0	1	2	3	4	5
氨基酸标准品浓度（mmol/L）	0	10	20	30	40	50
吸光度 OD 值	0.012	0.083	0.150	0.220	0.284	0.351
绝对吸光度 OD 值	0	0.071	0.138	0.208	0.272	0.339

3、绘图如下:

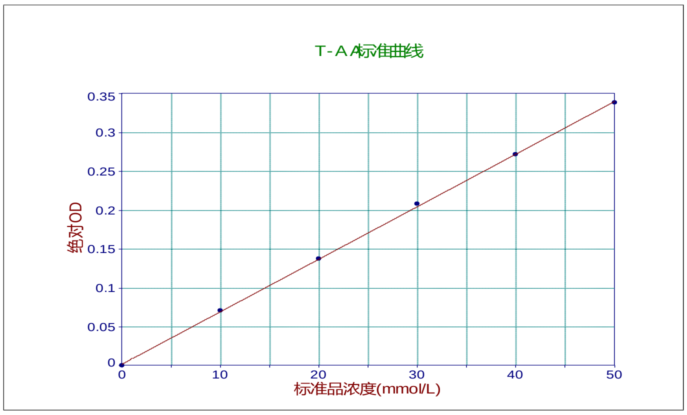

标准曲线用户可以不做,只需按前面给的计算公式计算即可。

公司地址：南京市玄武区中央路 258-27 号新立基大厦 邮政编码：210009 E-Mail：njjcbio@vip.163.com
联系电话：025-83360321、83360969、83551389 技术支持：025-83360272 传真号码：025-83227943
