# Supplementary material for: Uricase deficiency in rats results in a variety of metabolic disorders, addition to gouty nephropathy
Source: PLoS One. 2025 Aug 22;20(8):e0330344. doi: 10.1371/journal.pone.0330344 (PMC12373213; doi:10.1371/journal.pone.0330344)
Supplement: S3 — (ZIP) [file pone.0330344.s004.zip › TC.pdf]

总胆固醇(T-CHO)测试盒说明书(精简版)

（货号:A111-1-1　COD-PAP 法　微板法）

一、试剂组成及配制(96T):

试剂组成	规　格	组　份	浓　度	保存条件
工作液（酶剂）	25mL×1 瓶	Good's 缓冲液	50mmol/L，pH6.7	2～8℃避光
		苯酚	5mmol/L	
		4-AAP	0.3mmol/L	
		胆固醇酯酶	≥50KU/L	
		胆固醇氧化酶	≥25KU/L	
		过氧化物酶	≥1.3KU/L	
		牛血清白蛋白	1g/L	
		叠氮钠	1g/L	
校准品	1 支	胆固醇	见标签	
附送 96 孔平底酶标板一块				室温放置

二、测定原理:

胆固醇酯 $\xrightarrow{CE}$ 胆固醇＋脂肪酸

胆固醇 ＋ O_2 $\xrightarrow{CO}$ Δ^4 − 胆甾烯酮 ＋ H_2O_2

$H_2O_2 + 4-AAP$ + 苯酚 $\xrightarrow{POD}$ 红色醌化物 H_2O

生成的醌类化合物颜色的深浅与胆固醇的含量成正比，分别测定校标准管和样本管的吸光度值，计算胆固醇的含量。

三、所需仪器耗材及试剂:

含 500nm 波长的酶标仪及 96 孔板，37℃水浴锅或恒温箱，台式低速离心机，各种规格移液器，蒸馏水，涡旋混匀器，试管或离心管。

四、操作过程:

1、样本处理:

①、血清(浆)：直接测定，如超过线性范围用生理盐水稀释后测定。

②、培养液样本：吸取培养液，1000 转/分，离心 10 分钟，取上清测定。**[注]：一般建议细胞密度在 100 万个/mL 以上。**

③、组织样本：准确称取组织重量，按重量(g):体积(mL)=1:9 的比例，加入 9 倍体积的匀浆介质，冰水浴条件下机械匀浆，2500 转/分，离心 10 分钟，取上清液待测。**[注]:如组织样本均为非高脂样本，匀浆介质统一用磷酸盐缓冲液(0.1mol/L pH 7.4)或生理盐水进行匀浆提取；如组织样本均为高脂样本或部分为高脂样本，匀浆介质可统一用无水乙醇进行匀浆提取。**

④、细胞样本：

A、细胞收集:将制备好的细胞悬液取出，1000 转/分，离心 10 分钟，弃上清液，留细胞沉淀；用等渗缓冲液（推荐 0.1mol/L、pH7～7.4 磷酸盐缓冲液）清洗 1～2 次，同样 1000 转/分，离心 10 分钟，弃上清液，留细胞沉淀；

B、细胞破碎:加入 0.2～0.3mL 的匀浆介质（推荐 0.1mol/L、pH7～7.4 磷酸盐缓冲液或生理盐水）进行匀浆，冰水浴条件下超声破碎(功率 300W，3～5 秒/次，间隔 30 秒,重复 3～5 次)或手动匀浆，制备好的匀浆液不离心直接测定。也可采用裂解液裂解(推荐 TritonX-100,1～2%,裂解 30～40 分钟)，裂解好的液体不离心直接测定。

[注]：建议收集的细胞密度在 100 万个/mL 以上。破碎好的液体可显微镜观察细胞是否破碎完全。

2、操作表:

	空白孔	校准孔	样本孔
蒸馏水（μL）	2.5		
校准品（μL）		2.5	
样本（μL）			2.5
工作液（μL）	250	250	250
混匀，37℃孵育 10 分钟，波长 500nm，酶标仪测定各孔吸光度值			

五、计算公式:

1、血清等液体样本计算公式:

$$\text{胆固醇含量(mmol/L)} = \frac{A_{样本} - A_{空白}}{A_{标准} - A_{空白}} \times C_{标准}$$

C 标准:标准品浓度,mmol/L(具体浓度见标签)。

2、组织、细胞计算公式:

①、用 PBS 或生理盐水作匀浆介质提取样本计算方法（此方法需要另外测定匀浆液蛋白浓度）：

$$\text{胆固醇含量(mmol/gprot)} = \frac{A_{样本} - A_{空白}}{A_{标准} - A_{空白}} \times C_{标准} \div Cpr$$

C 标准:标准品浓度,mmol/L(具体浓度见标签)。

Cpr：匀浆液蛋白浓度，gprot/L（prot 指蛋白）。

注:本所有售蛋白测定试剂盒(货号为 A045-2 或 A045-3/-4)。

②、用无水乙醇作匀浆介质提取样本计算方法（此方法不需要另外测定匀浆液蛋白浓度）：

$$\text{胆固醇含量(mmol/g组织)} = \frac{A_{样本} - A_{空白}}{A_{校准} - A_{空白}} \times C_{标准} \div \frac{W}{V_{乙醇}}$$

W：样本质量，g；V 乙醇：加入的乙醇的总体积，L。

注：如样本中含有高脂样本，建议用乙醇来提取。细胞样本测定时可将上式中 $\frac{W}{V_{乙醇}}$ 替换为细胞前处理时的细胞密度。

六、性能指标:

1、试剂空白管吸光度≤0.100（光径 0.5cm）。

2、线性：0～19.39mmol/L 范围内，r^2＞0.995。

3、精密度：CV≤3%，批间相对极差≤5%。

4、稳定性：原装试剂盒在 2℃～8℃避光保存，有效期为 12 个月。开启后 2℃～8℃避光保存，可稳定 3 个月。

七、注意事项:

1、本产品仅用于科研，不得用于临床诊断，切勿服用。

2、样品含量如超出检测范围上限时，可用生理盐水稀释样本后进行测定，测定结果乘以稀释倍数。

3、试剂防止葡萄糖、胆固醇等试剂的污染。

4、试剂与样本量可按照全自动生化分析仪的要求，按照 1：100 的比例增减。

5、标准品为醇溶性试剂，打开后易挥发，96 孔板操作时尽量在加完样本后加标准品，且标准孔优先加入工作液以降低标准品的挥发，从而降低偏差。

八、参考文献:

1、Searay R .L.Diagonstic Biochemistry. Mc Graw-Hill . New York. NY.1969

2、Richmand W.Clin.Chem.1973;19:1350
