# Supplementary material for: Uricase deficiency in rats results in a variety of metabolic disorders, addition to gouty nephropathy
Source: PLoS One. 2025 Aug 22;20(8):e0330344. doi: 10.1371/journal.pone.0330344 (PMC12373213; doi:10.1371/journal.pone.0330344)
Supplement: S3 — (ZIP) [file pone.0330344.s004.zip › TG.pdf]

甘油三酯(TG)测试盒说明书

（货号:A110-1-1 GPO-PAP 酶法 单试剂型 微板法）

免责声明：测试前请仔细阅读说明书,预试后再进行批量实验,否则由此导致的后果用户自行承担!

一、试剂组成及配制(96T):

试剂组成	规 格	组 份	浓 度	保 存
工作液（酶剂）	25mL×1 瓶	Tris-HCL 缓冲液	100mmol/L	2-8℃避光
		脂肪酶	≥3000U/L	
		ATP	0.5mmol/L	
		甘油激酶	≥1000U/L	
		3-磷酸甘油氧化酶	≥5000U/L	
		过氧化物酶	≥1000U/L	
		4-氨基安替比林	1.4mmol/L	
		对氯酚	3mmol/L	
校准品	0.1mL×1 支	-	见标签	
附送 96 孔平底酶标板一块				室温

二、测定原理：(GPO-PAP 法)

$$甘油三酯 + H_2O \xrightarrow{脂肪酶} 甘油 + 脂肪酸$$

$$甘油 + ATP \xrightarrow{甘油激酶} 甘油-3-磷酸 + ADP$$

$$甘油-3-磷酸 + O_2 \xrightarrow{3-磷酸甘油氧化酶} 磷酸羟基丙酮 + H_2O_2$$

$$H_2O_2 + 4-AAP + 对氯酚 \xrightarrow{过氧化物酶} 红色醌化物$$

生成的醌类化合物颜色的深浅与甘油三酯的含量成正比，分别测定标准管和样本管的吸光度值，可计算样本中甘油三酯的含量。

三、操作过程:

1、样本处理:

①、血清(浆): 直接测定，如超过线性范围用生理盐水稀释后测定。

②、培养液样本: 吸取培养液，1000 转/分，离心 10 分钟，取上清测定。**[注]：一般建议细胞密度在 100 万个/mL 以上。**

③、组织样本: 准确称取组织重量,按重量(g)：体积(mL)=1：9 的比例，加入 9 倍体积的匀浆介质，冰水浴条件下机械匀浆,2500 转/分,离心 10 分钟，取上清液待测。**[注]:如组织样本为非高脂样本，匀浆介质用磷酸盐缓冲液(0.1mol/L pH 7.4)或生理盐水（0.9%）进行提取；如组织样本为高脂样本或部分为高脂样本，匀浆介质可统一用无水乙醇进行提取。**

④、细胞样本:

A、细胞收集: 将制备好的细胞悬液取出，1000 转/分，离心 10 分钟，弃上清液，留细胞沉淀；用等渗缓冲液（推荐 0.1mol/L 、pH7～7.4 磷酸盐缓冲液）清洗 1～2 次，同样 1000 转/分，离心 10 分钟，弃上清液，留细胞沉淀;

B、细胞破碎: 加入 0.2～0.3mL 的匀浆介质（**推荐 0.1mol/L pH7～7.4 磷酸盐缓冲液或生理盐水**）进行匀浆，冰水浴条件下超声破碎(功率:300W,3～5 秒/次,间隔 30 秒，重复 3～5 次)或手动匀浆，制备好的匀浆液不离心直接测定。也可采用裂解液裂解(推荐 TritonX-100，1～2%,裂解 30～40 分钟),裂解好的液体不离心直接测定。**[注]：建议细胞密度在 100 万个/mL 以上。破碎好的液体可显微镜观察细胞是否破碎完全**

2、操作表:

	空白孔	标准孔	样本孔
蒸馏水（μL）	2.5		
校准品（μL）		2.5	
样本（μL）			2.5
工作液（μL）	250	250	250
震荡孔板混匀，37℃孵育 10 分钟，波长 500nm，酶标仪测定各孔吸光度值。			

四、计算公式及举例:

1、血清等液体样本计算公式:

$$\frac{甘油三酯含量}{(mmol/L)} = \frac{A_{样本孔} - A_{空白孔}}{A_{标准孔} - A_{空白孔}} \times C_{标准}$$

$C_{标准}$:标准品浓度，mmol/L。

2、组织、细胞计算公式:

①、用 PBS 或生理盐水作匀浆介质提取样本计算方法（此方法需要另外测定匀浆液蛋白浓度）:

$$\frac{甘油三酯含量}{(mmol/gprot)} = \frac{A_{样本孔} - A_{空白孔}}{A_{标准孔} - A_{空白孔}} \times C_{标准} \div Cpr$$

Cpr：待测组织样本匀浆蛋白浓度,gprot/L。(prot 指蛋白）测定蛋白浓度的试剂盒本所有售 （A045-2/A045-3/-4）

②、用无水乙醇作匀浆介质提取样本计算方法（此方法不需要另外测定匀浆液蛋白浓度）:

$$\frac{甘油三酯含量}{(mmol/g鲜重)} = \frac{A_{样本孔} - A_{空白孔}}{A_{标准孔} - A_{空白孔}} \times C_{标准} \div \frac{W}{V_{提取液}}$$

W：组织重量（g）;$V_{提取液}$：加入的提取液(乙醇)的总体积，L。

注: 细胞样本测定时可将上式中的 $\frac{W}{V_{提取液}}$ 替换为细胞前处理时的细胞密度。

五、产品描述:

本试剂盒采用 GPO-PAP 法配制，用于体外测定甘油三酯含量。适用于各型酶标仪。

六、性能指标:

1、**试剂空白管吸光度≤0.200**（光径 0.5cm）。
2、**线性:** 0.3～11.4mmol/L 范围内，r^2>0.995。
3、**准确度:** 相对偏差≤10%。
4、**灵敏度:** 测试 2.7mmol/L 被测物时，吸光度值△A 在 0.2000～0.4000 之间。
5、**重复性:** 测量精密度≤5.0%，批间差≤8.0%。
6、**稳定性:** 原包装试剂盒在 2℃～8℃避光保存，有效期为 12 个月。开启后 2℃～8℃避光可稳定一个月。

七、注意事项:

1、本产品仅用于科研，不得用于临床诊断，切勿服用。
2、样品含量如超出检测范围上限时，可用生理盐水稀释样本后进行测定，测定结果乘以稀释倍数。
3、试剂防止葡萄糖、胆固醇等试剂的污染。
4、试剂与样本量可按照全自动生化分析仪的要求，按照 1：100 的比例增减。
5、测定样本时若测定 OD 值大于 0.8，请将样本稀释后测定。

八、参考文献:

1、叶应妩、王毓三 全国临床检验操作规程 第三版，东南大学出版社，2006，P479。

公司地址：南京市玄武区中央路 258-27 号新立基大厦 邮政编码：210009 E-Mail：njjcbio@vip.163.com
联系电话：025-83360321、83360969、83551389 技术支持：025-83360272 传真号码：025-83227943
