# Supplementary material for: Uricase deficiency in rats results in a variety of metabolic disorders, addition to gouty nephropathy
Source: PLoS One. 2025 Aug 22;20(8):e0330344. doi: 10.1371/journal.pone.0330344 (PMC12373213; doi:10.1371/journal.pone.0330344)
Supplement: S3 — (ZIP) [file pone.0330344.s004.zip › UA.pdf]

尿酸（UA）测试盒说明书(精简版)

（货号：C012-1-1　比色法　50 管/48 样）

一、测定原理：

无蛋白滤液中的尿酸在碱性状态下还原磷钨酸生成钨蓝、尿囊素和二氧化碳，蓝色深浅与尿酸浓度成正比例。

二、试剂组成：（试剂盒有效期 3 个月）

试剂一： 1g/L 尿酸标准贮备液，0.5mL×1 支，4℃保存 3 个月；临用前按 1g/L 标准贮备液∶蒸馏水=1∶19 的比例混合,配成 **50mg/L 尿酸标准应用液**，现用现配；

试剂二： 钨酸蛋白沉淀剂，60mL×2 瓶，4℃保存 6 个月；

试剂三： CUT 试剂，30mL×1 瓶，4℃保存 3 个月；

试剂四： 磷钨酸试剂，30mL×1 瓶，4℃保存 3 个月。

三、所需仪器及试剂：

可调 690nm 波长的可见光分光光度计及 1cm 光径比色皿(或酶标仪及 96 孔板)，蒸馏水，涡旋混匀器。

四、操作表：

	测定管	标准管	空白管
血清（浆）（mL）	0.2		
50mg/L 尿酸标准应用液（mL）		0.2	
蒸馏水（mL）			0.2
钨酸蛋白沉淀剂（mL）	2.0	2.0	2.0
混匀，10 分钟后，3000 转/分，离心 5 分钟，取上清			
上清液（mL）	1.6	1.6	1.6
CUT 试剂（mL）	0.5	0.5	0.5
磷钨酸试剂（mL）	0.5	0.5	0.5
混匀，室温静置 10 分钟，波长 690nm、1cm 光径、蒸馏水调零，测定各管吸光度值 A。			

尿液尿酸测定： 记取尿量，混匀尿液，尿酸溶解度低，易结晶沉淀，因此要加温到 50℃，立即用水稀释 10 倍，操作同血清，结果乘以 10。

[注]： 尿酸的呈色稳定性较差，显色后最好 20 分钟内完成比色，所以一批实验最好不要超过 20 份标本。

五、计算公式：

$$尿酸含量 = \frac{A_{测定} - A_{空白}}{A_{标准} - A_{空白}} \times C_{标准}$$

C 标准:标准品浓度,50mg/L(即 297.4μmol/L)。

公司地址：南京市玄武区中央路 258-27 号新立基大厦　邮政编码：210009　E-Mail：njjcbio@vip.163.com
联系电话：025-83360321、83360969、83551389　技术支持：025-83360272、19951670086
