# Supplementary material for: Uricase deficiency in rats results in a variety of metabolic disorders, addition to gouty nephropathy
Source: PLoS One. 2025 Aug 22;20(8):e0330344. doi: 10.1371/journal.pone.0330344 (PMC12373213; doi:10.1371/journal.pone.0330344)
Supplement: S3 — (ZIP) [file pone.0330344.s004.zip › protocols_team.docx]

Protocols from Duans team

Approved by: Weigang Duan

Tel: 15925146965

Email：deardwg@126.com

Contents

[1. Rat housing 3](#_Toc202900991)

[2. Collection of rats’ blood samples 4](#_Toc202900992)

[3. Collection of rats’ fresh organs 5](#_Toc202900993)

[4. RNA sequencing 6](#_Toc202900994)

# Rat housing

(1) Rats should be kept in SPF environment, simulated natural light, temperature 22±1℃, humidity 45-55%.

(2) Clean the R5 rat cage, add an appropriate amount (about 250g shavings mat), knead it and spread it out evenly;

(3) Rats were placed in the cages, with a maximum of 5 rats in each cage, and 2-4 rats were recommended to be kept. Rats were marked with ear punches;

(4) Buckle the lid of the rat cage to prevent the rats from escaping. At the same time, mark the rat cage with a marker pen, and mark the content should include the name of the experiment, the person in charge and the starting date;

(5) Fill the cage feed bucket with enough feed, or according to the plan, and fill the water bottle with about 4/5 of the capacity of clean tap water;

(6) Observe animals at least twice a day, once in the morning and once in the evening, or once at work and at work. Pay attention to feed and water supplements for the rats. If found, it should be recorded and handled in time, and if abnormal, it should be reported to the project leader.

(7) If the pad is wet or obviously dirty, the rat should be vacated into another clean cage, and the new pad should be added after cleaning the cage, and then the rat should be put back.

# Collection of rats’ blood samples

(1) The rat was fixed to a rat fixator and its tail was exposed;

(2) Place the fixator in a small environment of about 30℃ to fully manifest the tail vessels;

(3) Cut off a small section of tail (less than 5 mm) from the distal end, the blood is dripping from the tail, hang the tail as far as possible, and collect the appropriate amount of blood with test tubes; A needle can also be inserted into the exposed rat tail vein to collect blood as needed. Blood may be treated with or without anticoagulation as required.

(4) After blood collection, apply sterile cotton swabs or cotton balls to stop bleeding. The rats were returned to the cage after no obvious bleeding.

(5) Serum was prepared by centrifugation at 3,000g for 5min after blood coagulation; If the sample is important, centrifugation at 4℃ is recommended. Erythrocyte and leukocyte components can also be obtained if anticoagulant blood is obtained, such as plasma prepared by method.

(6) The obtained serum or plasma should be used as soon as possible. It is expected to be used within the same day and can be stored at 4℃. If used to detect routine biochemical indicators, it can be stored at -20℃ for 1 month and -80℃ for 6 months, and long-term storage should be placed in liquid nitrogen. However, red blood cells and other blood components should be used within 24 hours (midway can be placed at 4℃).

# Collection of rats’ fresh organs

(1) Rats were weighed.

(2) The thumb and index finger of the left hand grabbed the skin behind the ear of the rats, and the other three fingers grabbed the skin on the back of the rats, and fixed the rats in the palm of the left hand;

(3) According to body weight, the rats were anesthetized by intraperitoneal injection of urethane (1.0 g/kg).

(4) After the animal was anesthetized (about 5min, the sign of anesthesia was that the righting reflex of the rat disappeared), the abdominal skin was cut open with scissors, and then the abdominal muscles were cut open to expose the abdominal cavity. Do not injure the diaphragm and chest at this time.

(5) According to the anatomical location, the abdominal aorta can be first found, and the blood of the rat can be collected by collecting vessels (anticoagulant or non-anticoagulant according to the need).

(6) Collect abdominal organs as required, such as liver, spleen, pancreas, kidney, stomach, small intestine, colon, bladder, etc.

(7) If necessary, cut the sternum and diaphragm, expose the chest cavity, and remove the heart and lungs.

(8) If the weight of the organs is to be recorded, the capsule or peritoneum should be removed and the blood should be wiped. If you want to record the weight of the gastrointestinal tract, you should also remove its contents.

(9) If the brain specimens of rats are to be collected, the head should be cut off and the skull slowly removed from the foramen magnum with tissue forceps to obtain brain tissue.

# RNA sequencing

(1) Rats were weighed and anesthetized by intraperitoneal injection of uratan anesthesia solution (1.0 g/Kg). Tissues of 0.1-0.2 g were taken and immediately put into dry ice, and delivered to Shanghai Sangong Bioengineering Co., Ltd. for second-generation high-throughput genome sequencing detection.

(2) Total RNA extraction:

Tissue cells were lysed by RNA lysate, and the lysed sample was placed at room temperature for 10 min to separate the nucleoprotein and nucleic acid completely. 0.2ml chloroform was added and shaken vigorously for 15 s, then placed at room temperature for 3 min and centrifuged at 4℃ for 10 min (12000 r/min). The supernatant was taken into the centrifuge tube and isopropyl alcohol was added with equal volume. Mixed, placed at room temperature for 20 min, centrifuged at 4℃ for 10 min (12000 r/min), washed with 1 mL 75% ethanol, centrifuged at 4℃ for 3 min (12000 r/min), discarded supernatant, dried at room temperature for 10 min. 50 μ L Rnase-free ddH2O was added, and the concentration and integrity of RNA were detected after the RNA was fully dissolved, and the RNA contamination was observed.

(3) mRNA library construction

Take mRNA Capture Beads out of the refrigerator at 4℃, balance them at room temperature for 30 min, mix well by whirlpool shaking, dissolve 1 μg total RNA into 50 μ L Nuclease free H2O, add 50 μ L mRNA Capture Beads. The samples were placed on a magnetic rack for 5 min. The mRNA and total RNA were separated. Discard the supernatant, add 200 μ L Beads Wash Buffer, and beat 6 times to mix. Put the samples on a magnetic rack for 5 min, remove the supernatant, add 50 μ L Tris Buffer, blow and mix for 6 times, place the samples in PCR apparatus, elute the mRNA (80℃ for 2 min, keep at 25℃), add 50 μ L Beads Binding Buffer, blow and mix for 6 times. Place the samples on a magnetic frame at room temperature for 5 min, remove the supernatant for 5 min, and obtain the separated mRNA. Add 200 μ L Beads Wash Buffer, beat them 6 times and mix well. Let the magnetic frame stand for 5 min, and use 10 μ L pipette to remove all the supernatant. 19.5 μ L Frag/Prime Buffer was added to re-suspend magnetic beads for 6 times of mixing. The samples were placed in the PCR machine at 94℃ for 5 min and held at 4℃. The samples were removed and placed on the magnetic rack for 5 min. First chain synthesis.

(4) Synthesis of double-stranded cDNA

Defrost the 1st Strand Buffer from the refrigerator at -20℃, mix well, and add it into each group according to the following operation table to prepare the first-strand cDNA synthesis reaction solution:

| solution | Volume(μl) |
| --- | --- |
| Fragmented mRNA | 17 |
| 1st Strand Buffer | 6 |
| 1st Strand Enzyme Mix | 2 |
| total | 25 |

The second chain synthesis reaction was carried out by taking the mixture at 25℃ for 10 min, 42℃ for 15 min and 70℃ for 15 min, respectively.

Defrost the 2nd Strand Buffer from the refrigerator at -20℃, mix well, and add into each group according to the following instructions to prepare the second Strand cDNA synthesis reaction solution:

| solution | Volume (μl) |
| --- | --- |
| 1st Strand cDNA | 25 |
| 2nd Strand Buffer | 20 |
| 2nd Strand Enzyme Mix | 5 |
| total | 50 |

The reaction system was placed at 16℃ for 60 min, 90 μ L (1.8x) DNA Clean Beads were added to purify double-stranded cDNA, 62.5 μ L nuclease free was added to dissolve magnetic Beads, and 60 μ L supernatant was absorbed into nuclease free centrifuge tubes.

(5) Terminal repair

Take End Prep Mix out of the -20℃ refrigerator, defrost it, Mix well, and add it into each group according to the following operation table to prepare the End repair reaction solution:

| solution | Volume (μl) |
| --- | --- |
| ds cDNA | 60 |
| End Prep Mix | 40 |
| total | 100 |

The reaction system was placed at 30℃ for 30 min, 160 μ L (1.6x) DNA Clean Beads were added to purify the end repair products, 20 μ L nuclease free dissolved magnetic Beads were added, and 17.5 μ L supernatant was absorbed into nuclease free centrifuge tubes.

(6) The end of the dA - Tailing

Take out the retailing Buffer from the refrigerator at -20℃, defrost it, mix it well, and add it to each group to prepare the Tailing reaction liquid according to the following operation table:

| solution | Volume (μl) |
| --- | --- |
| Purified products of terminal repair | 17.5 |
| dA-Tailing Buffer Mix | 10 |
| dA-Tailing Enzyme Mix | 2.5 |
| total | 30 |

The reaction system was placed in 37℃ warm bath for 30 min and 70℃ warm bath for 5 min, and the street connection reaction was carried out immediately.

(7) Adaptor connection reaction

Take the RNA Adapter out of the refrigerator at -20℃, thaw it, mix well, and add it into each group according to the following operation table to prepare the connecting reaction liquid:

| solution | Volume (μl) |
| --- | --- |
| Product of dA-Tailing | 30 |
| Ligation Mix | 2.5 |
| RNA Adapter（with barcode；1 μM） | 2.5 |
| total | 35 |

The system was placed at 30℃ for 10 min, and 5 μ L Stop Ligation Mix was added to terminate the reaction.

(8) Purification and fragment size sorting of linker products

Add 40 μ L (1 X) DNA Clean Beads to purify the joining products, add 102.5 μ L nuclease free water to dissolve the magnetic Beads, and take 100 μ L supernatant nuclease free tubes. 70 μ L (0.7x) DNA Clean Beads were added for the first round of fragment size separation. 155 μ L of supernatant was taken into nuclease free centrifugation tubes. 10 μ L (0.1x) DNA Clean Beads were added to purify the join products for the second round of fragment size sorting. 20 μ L supernatant was taken into nuclease free centrifuge tubes.

(9) Library amplification

PCR Primer Mix, Amplification Mix 1 was taken out of the refrigerator at -20℃, thawed, mixed evenly, and then added into each group according to the following operation table to prepare PCR reaction solution:

| solution | Volume (μl) |
| --- | --- |
| Purified product with adaptor | 20 |
| PCR Primer Mix | 5 |
| Amplification Mix 1 | 25 |
| total | 50 |

PCR reaction conditions:

| 98℃，30 s |  |
| --- | --- |
| 98℃，10 s  60℃，30 s  72℃，30 s | 15cycles |
| 72℃，5 min  4℃，∞ |  |

50 μ L (1 X) DNA Clean Beads were added into the reaction system to purify the joining products, 25 μ L nuclease free water was added to dissolve the magnetic Beads, and 22.5 μ L supernatant was taken into nuclease free centrifuge tubes.

(1) Quantitative mixing

Qubit 2.0 DNA detection kit was used for quantitative detection of DNA, and the DNA was mixed in a 1:1 ratio and sequenced. The sequencing procedure was completed by Shanghai Sangong Bioengineering Co., LTD.
