# Supplementary material for: Uricase deficiency in rats results in a variety of metabolic disorders, addition to gouty nephropathy
Source: PLoS One. 2025 Aug 22;20(8):e0330344. doi: 10.1371/journal.pone.0330344 (PMC12373213; doi:10.1371/journal.pone.0330344)
Supplement: S4 — (ZIP) [file pone.0330344.s005.zip › Figure S1.pptx]

## Slide 1
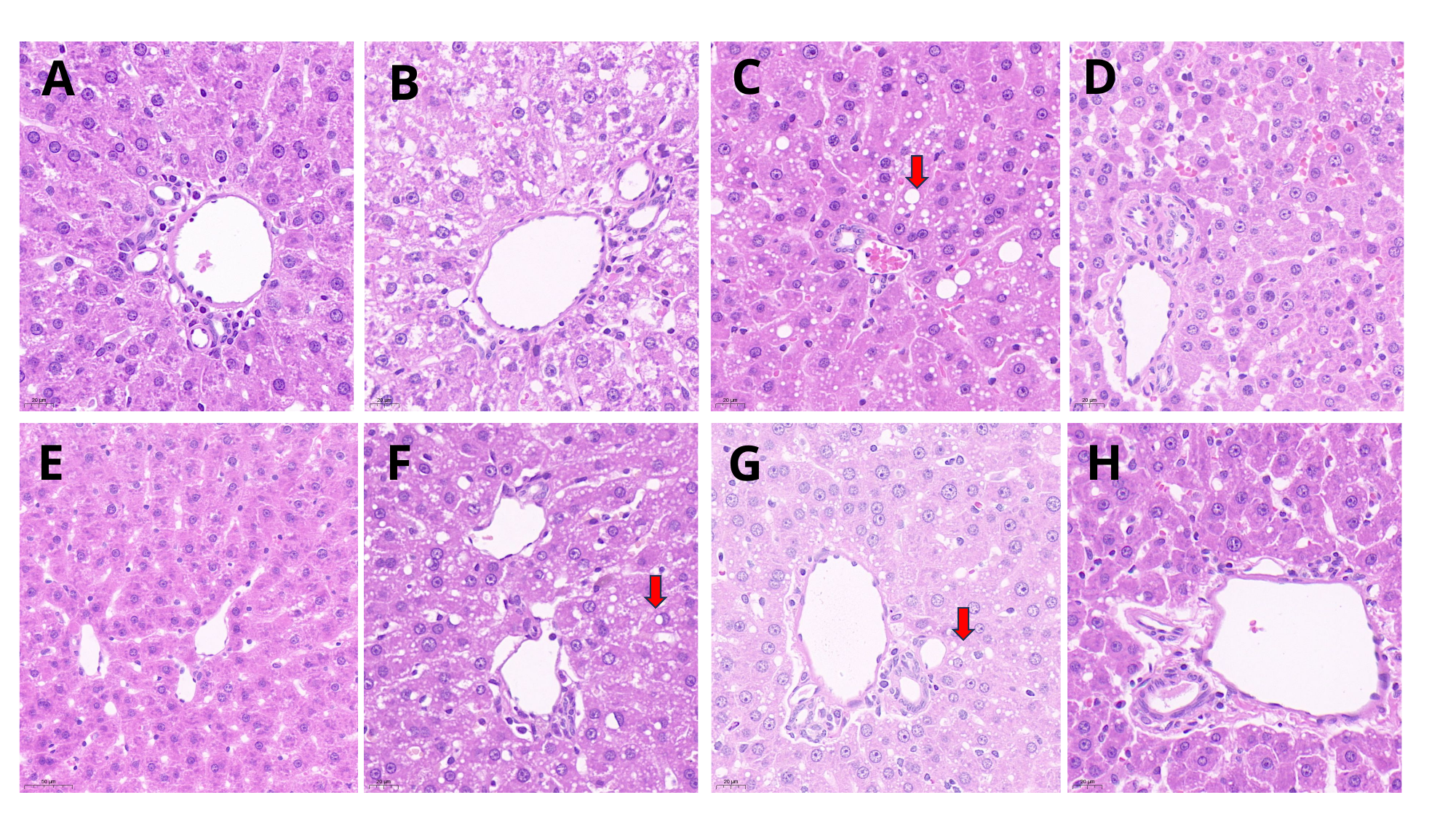

C
D
A
B
H
F
E
G

## Slide 2
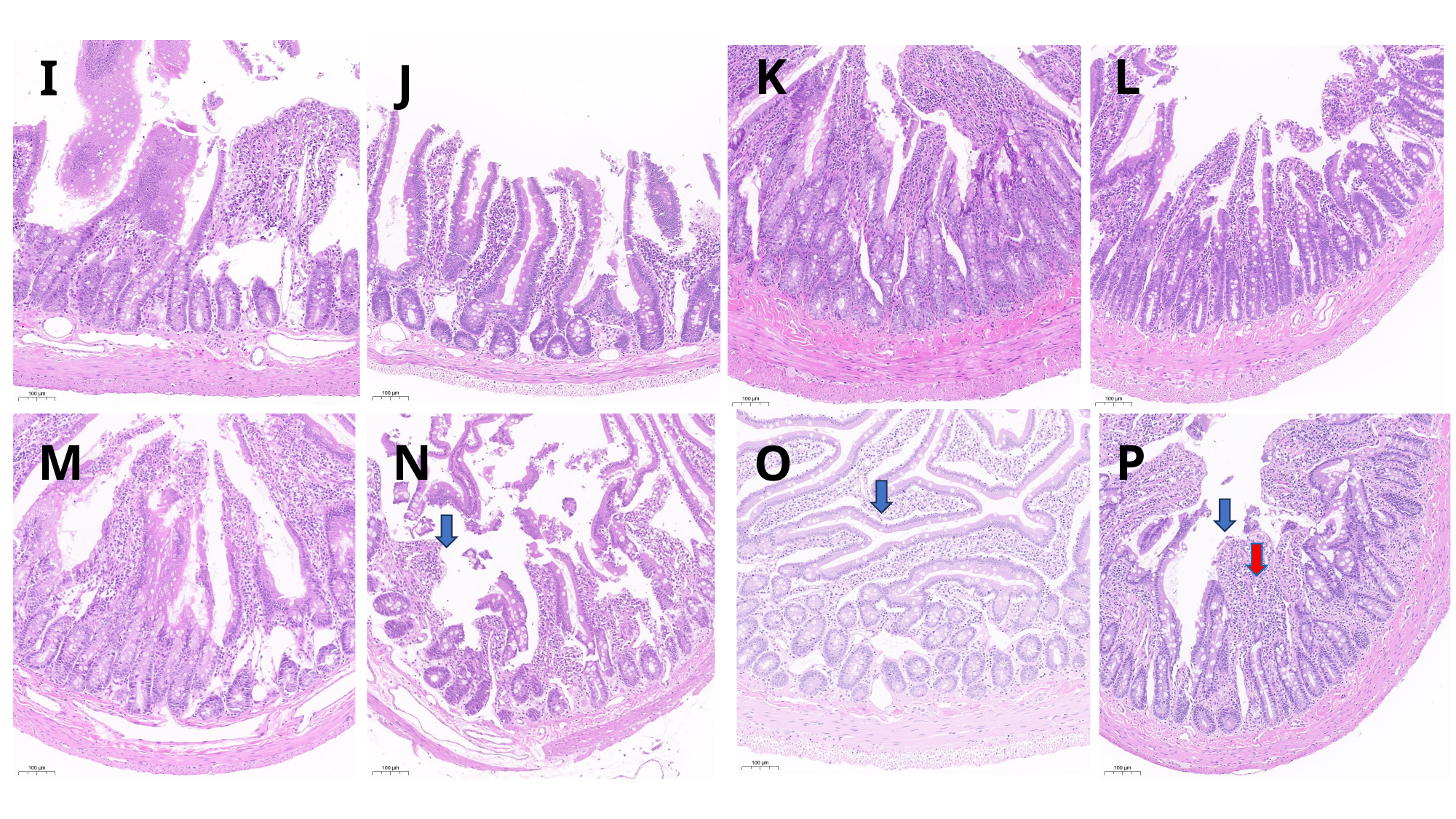

K
L
I
J
P
N
M
O

## Slide 3
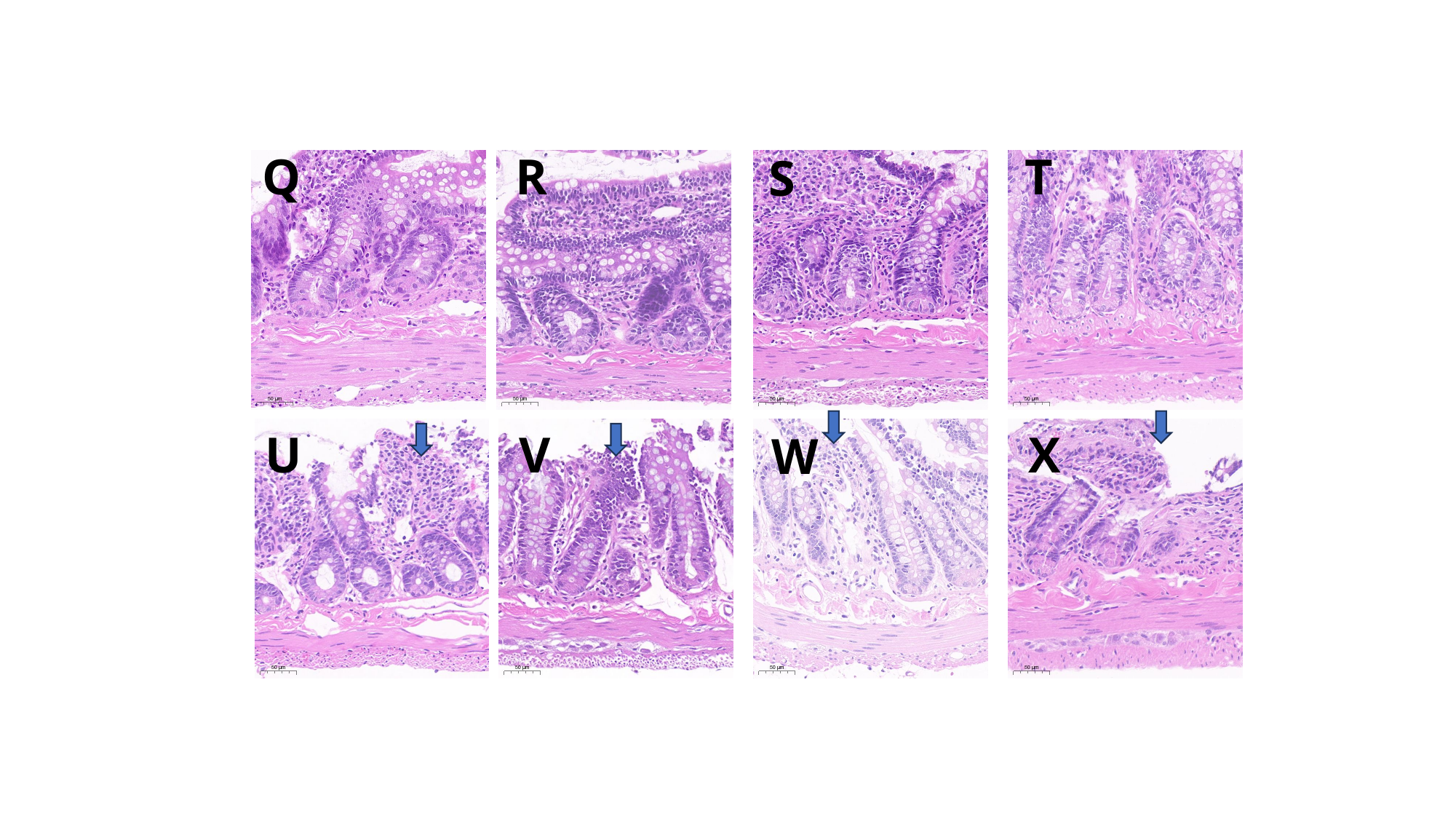

T
Q
R
S
X
U
V
W

## Slide 4
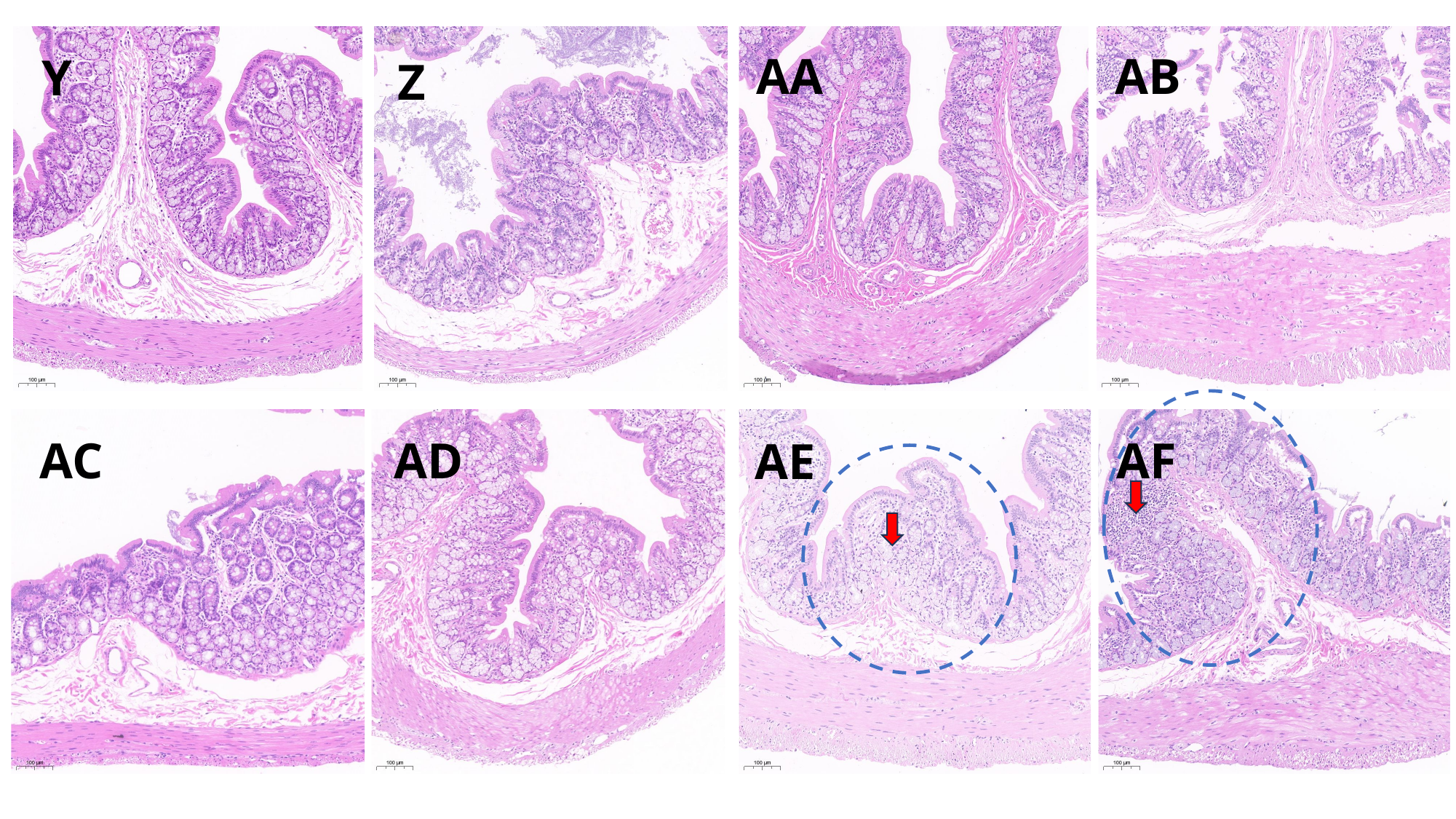

AA
AB
Y
Z
AF
AD
AC
AE

## Slide 5
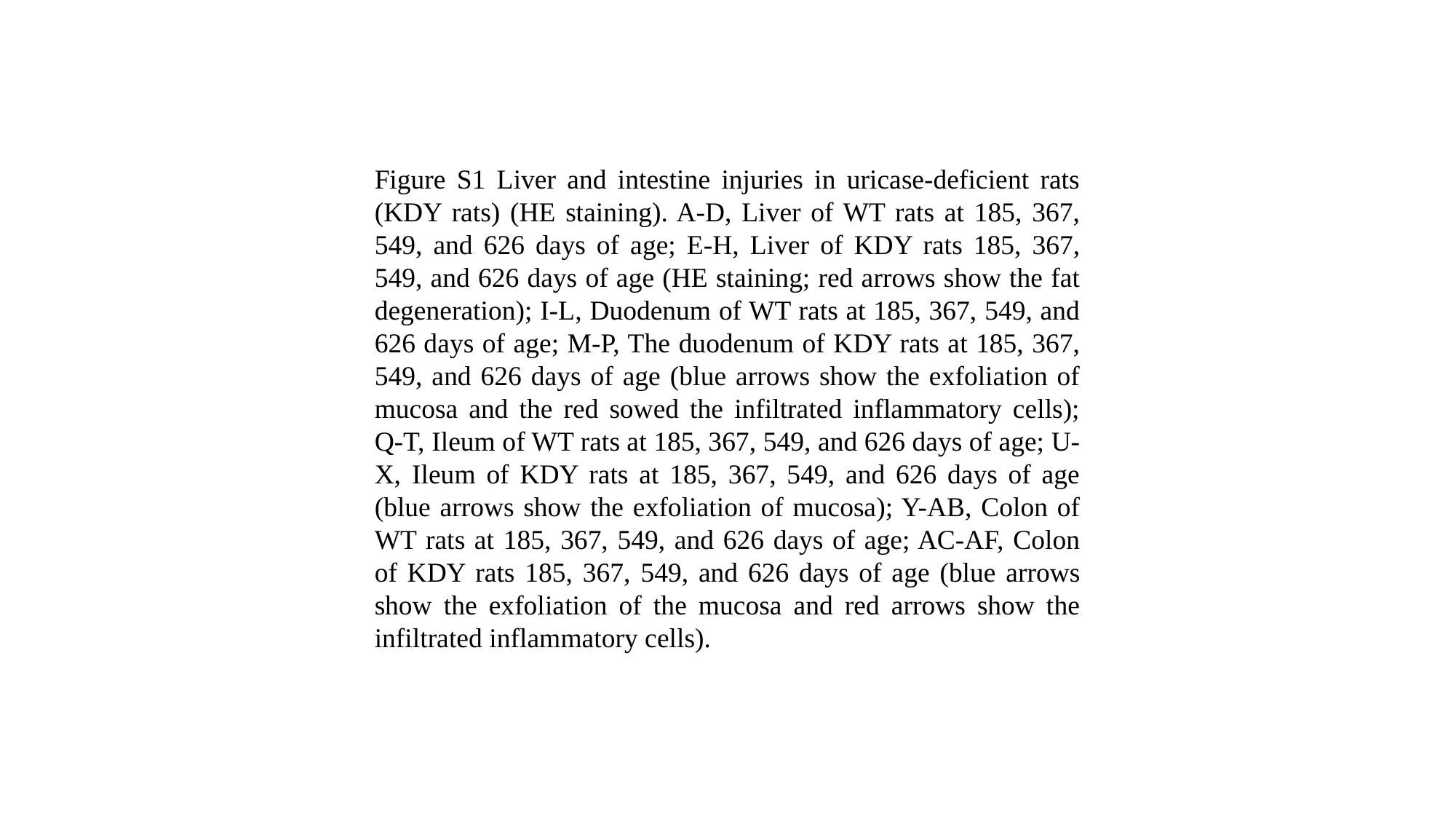

Figure S1 Liver and intestine injuries in uricase-deficient rats (KDY rats) (HE staining). A-D, Liver of WT rats at 185, 367, 549, and 626 days of age; E-H, Liver of KDY rats 185, 367, 549, and 626 days of age (HE staining; red arrows show the fat degeneration); I-L, Duodenum of WT rats at 185, 367, 549, and 626 days of age; M-P, The duodenum of KDY rats at 185, 367, 549, and 626 days of age (blue arrows show the exfoliation of mucosa and the red sowed the infiltrated inflammatory cells); Q-T, Ileum of WT rats at 185, 367, 549, and 626 days of age; U-X, Ileum of KDY rats at 185, 367, 549, and 626 days of age (blue arrows show the exfoliation of mucosa); Y-AB, Colon of WT rats at 185, 367, 549, and 626 days of age; AC-AF, Colon of KDY rats 185, 367, 549, and 626 days of age (blue arrows show the exfoliation of the mucosa and red arrows show the infiltrated inflammatory cells).
