# Supplementary material for: Uricase deficiency in rats results in a variety of metabolic disorders, addition to gouty nephropathy
Source: PLoS One. 2025 Aug 22;20(8):e0330344. doi: 10.1371/journal.pone.0330344 (PMC12373213; doi:10.1371/journal.pone.0330344)
Supplement: S4 — (ZIP) [file pone.0330344.s005.zip › Figure S2.pptx]

## Slide 1
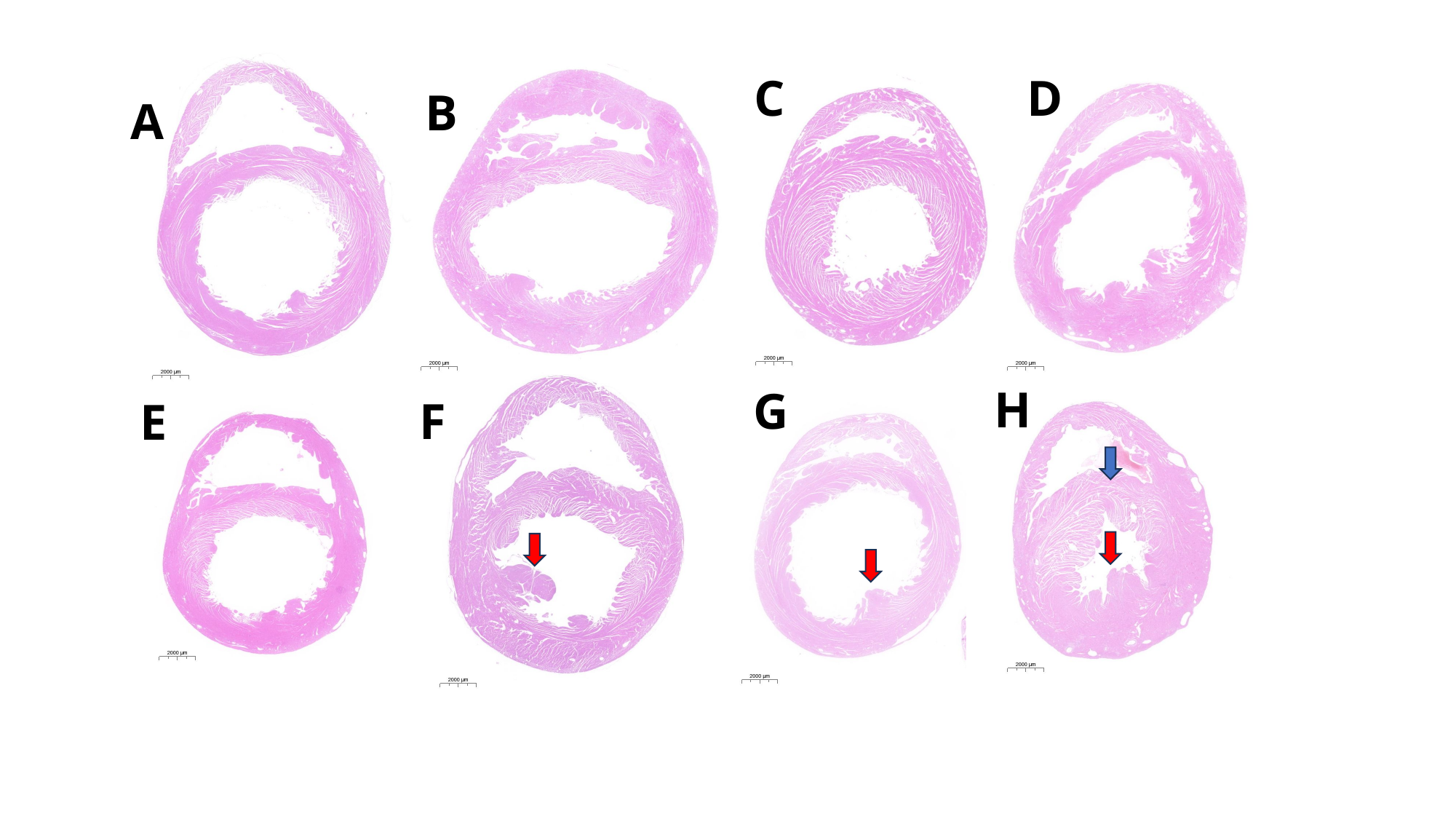

C
D
B
A
H
G
F
E

## Slide 2
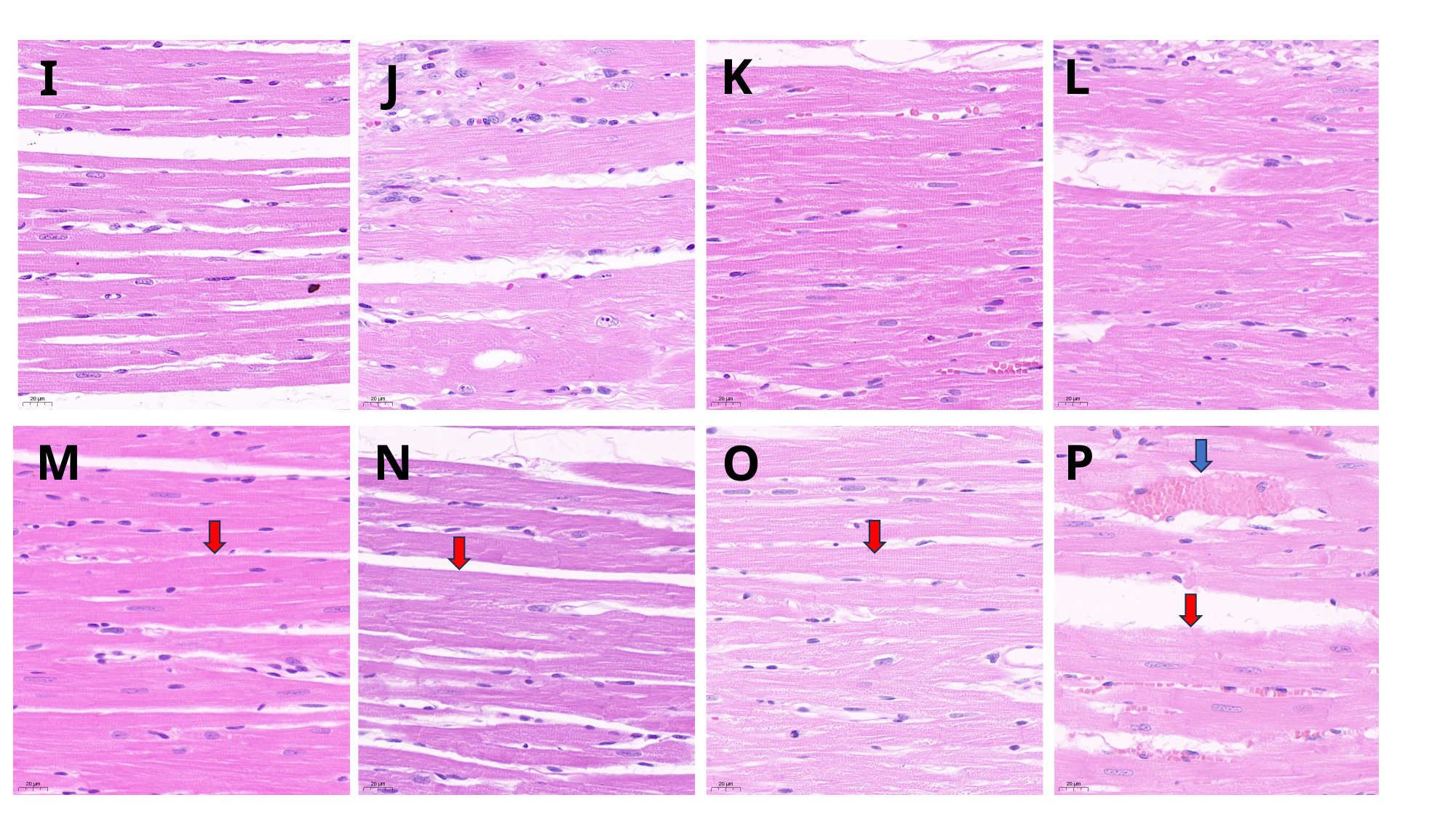

K
L
I
J
P
N
M
O

## Slide 3
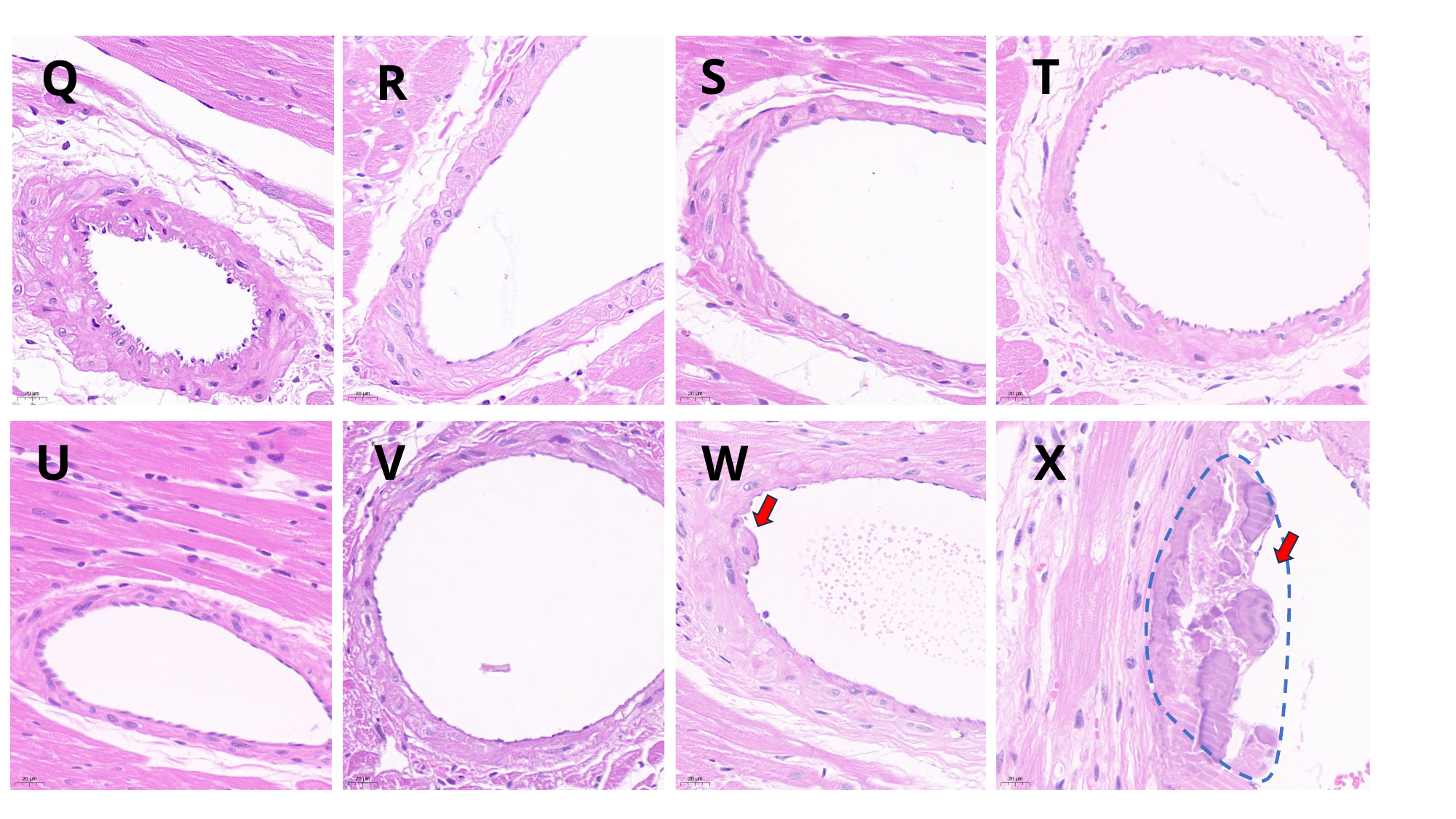

S
T
Q
R
X
V
U
W

## Slide 4
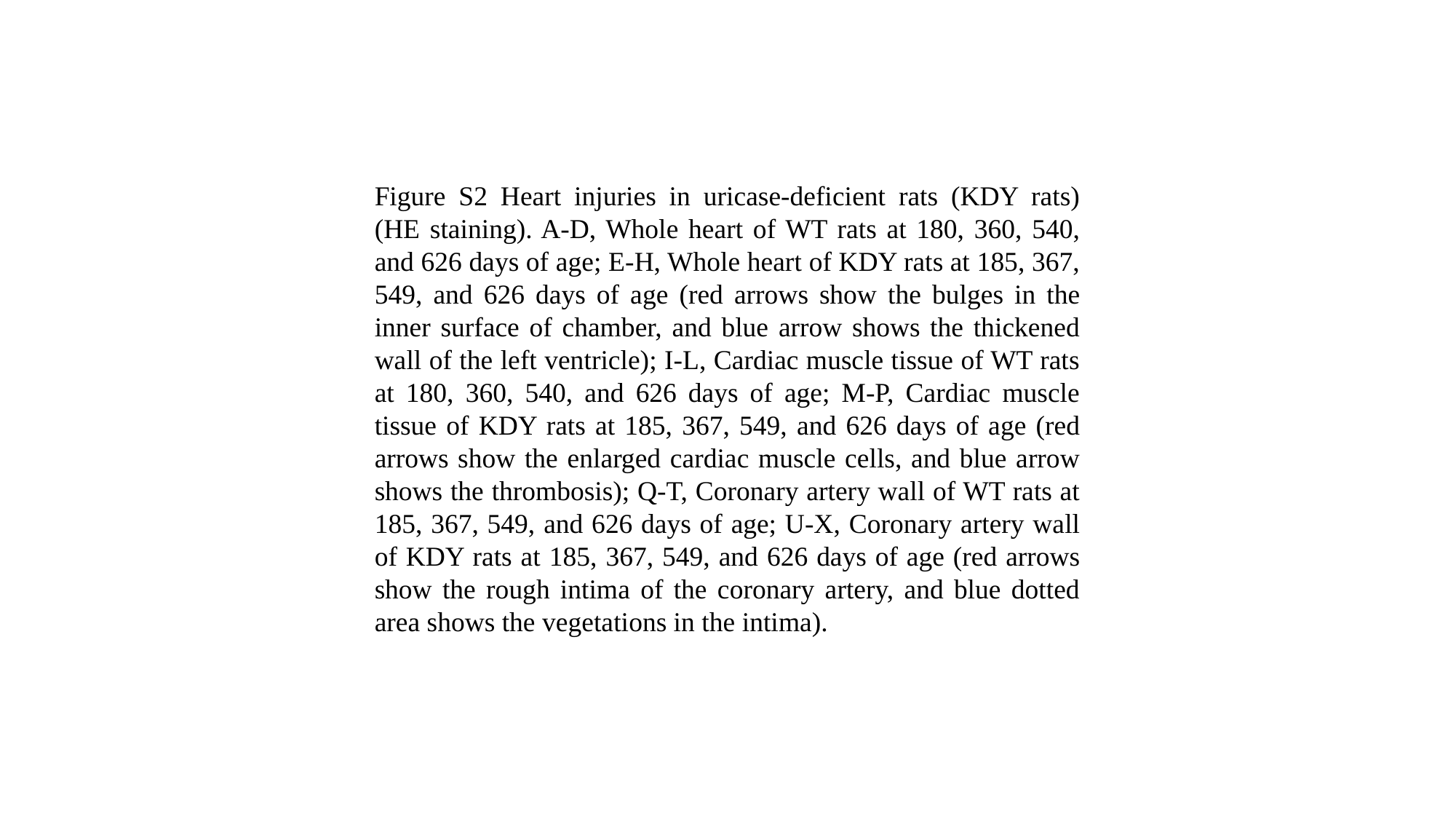

Figure S2 Heart injuries in uricase-deficient rats (KDY rats) (HE staining). A-D, Whole heart of WT rats at 180, 360, 540, and 626 days of age; E-H, Whole heart of KDY rats at 185, 367, 549, and 626 days of age (red arrows show the bulges in the inner surface of chamber, and blue arrow shows the thickened wall of the left ventricle); I-L, Cardiac muscle tissue of WT rats at 180, 360, 540, and 626 days of age; M-P, Cardiac muscle tissue of KDY rats at 185, 367, 549, and 626 days of age (red arrows show the enlarged cardiac muscle cells, and blue arrow shows the thrombosis); Q-T, Coronary artery wall of WT rats at 185, 367, 549, and 626 days of age; U-X, Coronary artery wall of KDY rats at 185, 367, 549, and 626 days of age (red arrows show the rough intima of the coronary artery, and blue dotted area shows the vegetations in the intima).
