# Supplementary material for: Uricase deficiency in rats results in a variety of metabolic disorders, addition to gouty nephropathy
Source: PLoS One. 2025 Aug 22;20(8):e0330344. doi: 10.1371/journal.pone.0330344 (PMC12373213; doi:10.1371/journal.pone.0330344)
Supplement: S4 — (ZIP) [file pone.0330344.s005.zip › Figure8.pptx]

## Slide 1
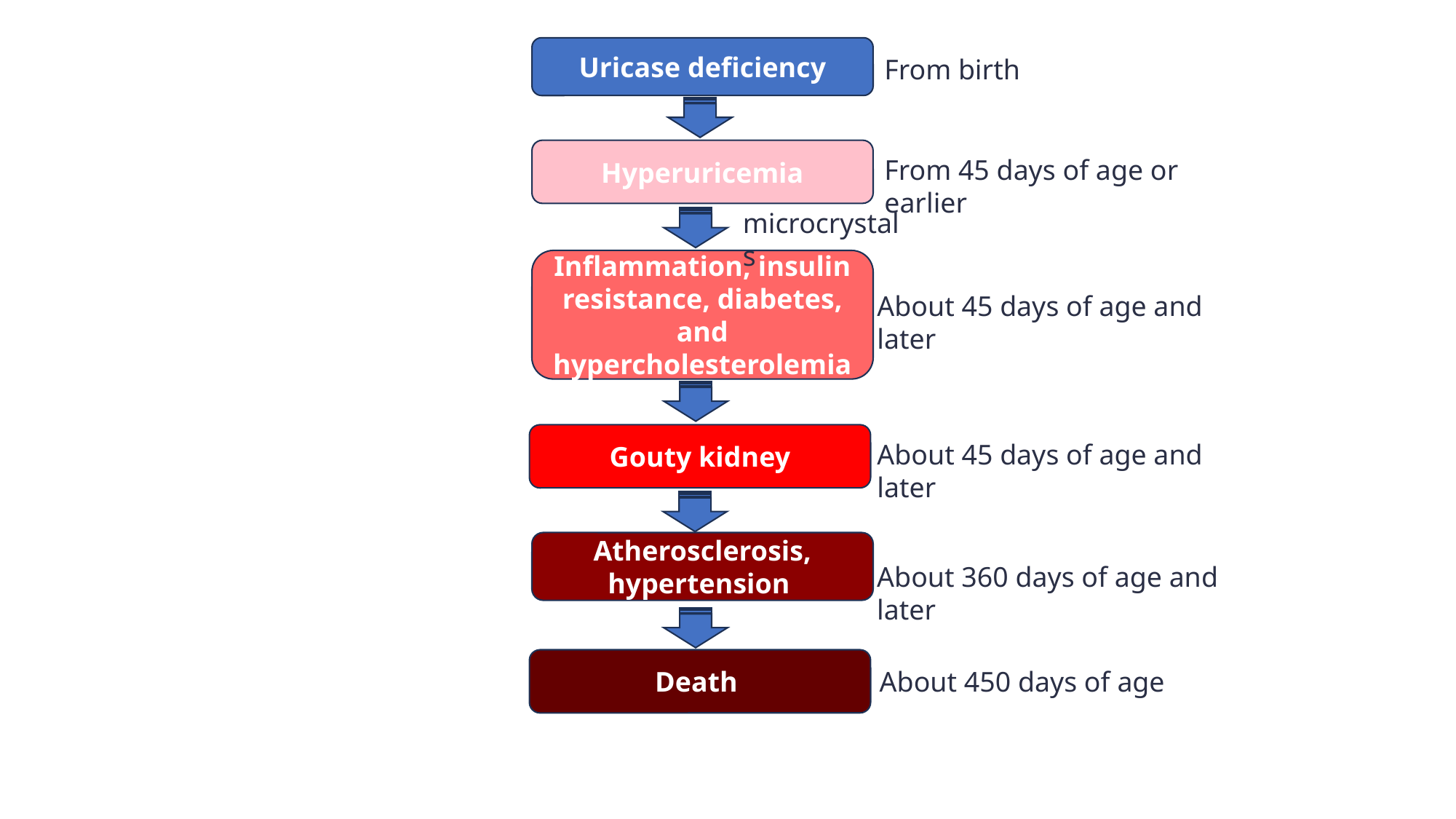

Uricase deficiency
From birth
Hyperuricemia
From 45 days of age or earlier
microcrystals
Inflammation, insulin resistance, diabetes, and hypercholesterolemia
About 45 days of age and later
Gouty kidney
About 45 days of age and later
Atherosclerosis, hypertension
About 360 days of age and later
Death
About 450 days of age
